# Unraveling the HGF/MET axis in Mallory-Denk body pathogenesis associated with liver fibrosis through single-cell transcriptomics

**DOI:** 10.1038/s41392-026-02722-4

**Published:** 2026-06-11

**Authors:** Xiaoping Tang, Yi Shi, Jia Pan, Yi Zhao, Maoping Huang, Huanhou Su, Wanmei Zhou, Xingguang Luo, Xiao Luo, Jinge Zhang, Jiuying Zhao, Jiangtao Jia, Jinru Li, Bei Zhong, Lan Tang, Zhuo Chen, Yongchang Ouyang, Zhichun Dang, Samuel W. French, Yihui Huang, Xinghua Pan, Hui Liu

**Affiliations:** 1 Department of Clinical Laboratory, the Fifth Affiliated Hospital of Guangzhou Medical University, Center for Liver Diseases of Guangzhou Eighth People’s Hospital, Guangzhou Medical University, Guangzhou, China; 2 The State Key Laboratory of Respiratory Disease and National Clinical Research Center for Respiratory Disease, Guangzhou Medical University, Guangzhou, China; 3 The Affiliated Qingyuan Hospital of Guangzhou Medical University, Qingyuan People’s hospital, Qingyuan, China; 4 Department of Biochemistry and Molecular Biology, School of Basic Medical Sciences, Southern Medical University, Guangdong Provincial Key Laboratory of Single Cell Technology and Application, Guangzhou, China; 5 Center of Hepato-Pancreato-Biliary Surgery, The First Affiliated Hospital of Sun Yat-Sen University, Guangzhou, China; 6 Beijing Huilongguan Hospital, Peking University Huilongguan Clinical Medical School, Beijing, China; 7 Department of Pathology, Harbor UCLA Medical Center, University of California, Torrance, CA USA; 8 Precision Regenerative Medicine Research Centre, Medical Science Division, Macau University of Science and Technology, Macao, China

**Keywords:** Inflammation, Gastrointestinal diseases

## Abstract

Mallory-Denk bodies (MDBs) are protein aggregates commonly observed in chronic liver diseases, including liver fibrosis. However, the intrahepatic crosstalk driving MDB pathogenesis and fibrosis progression remains poorly understood. Using single-nucleus RNA sequencing (snRNA-seq), we identified significant cellular heterogeneity and a distinct hepatocyte subpopulation, termed MDB-associated hepatocytes (MAHs). MAHs were strongly correlated with hepatocellular carcinoma progression. Four hepatic stellate cell (HSC) subpopulations were defined, among which activated HSCs (aHSCs) represent a unique MDB-associated subtype. Moreover, we revealed a tightly connected axis involving MAHs, aHSCs, and Kupffer cells (KCs), which demonstrated that aberrant hepatocyte growth factor (HGF)/mesenchymal‒epithelial transition factor (MET) signaling contributes to MDB pathogenesis. Mechanistically, HGF secreted by aHSCs or KCs interacts with MET on ballooned MAHs and stimulates the HGF/MET downstream PI3K/AKT/NF-κB and STAT3 pathways via protein phosphorylation. The activated HGF/MET pathway promotes ubiquitin D (UbD) upregulation and the release of the proinflammatory cytokine TNFα which further promotes HGF transcription, establishing a positive feedback loop and contributing to MDB formation. Furthermore, aHSCs promote MDB pathogenesis by regulating STAT3 via the HGF/MET axis and increase HSC activation by stimulating TGFβ1 secretion, thereby accelerating fibrosis in 3D MDB organoid cultures. Notably, UbD deficiency (in UbD⁻/⁻ mice) suppressed HGF/MET signaling and MDB formation, leading to reduced liver fibrosis. Consistently, HGF/MET signaling was markedly elevated in human liver biopsies containing MDBs. Together, these findings provide unprecedented single-cell insights into liver cell reprogramming and intrahepatic crosstalk during MDB pathogenesis, and highlight the HGF/MET/UbD axis as a potential therapeutic target for chronic liver disease.

## Introduction

Hepatocytes, the principal parenchymal cells (PCs) of the liver, function alongside diverse nonparenchymal cells (NPCs) in a highly organized yet heterogeneous tissue microenvironment. In the context of liver injury, hepatocytes release cytokines and inflammatory extracellular vesicles that propagate hepatic inflammation.^1^ Following injury, hepatocytes are the first to proliferate, followed sequentially by liver sinusoidal endothelial cells (LSECs), Kupffer cells (KCs), and hepatic stellate cells (HSCs). HSCs represent the main source of collagen-producing myofibroblasts in the injured liver.^2^ Upon injury, quiescent HSCs undergo activation, a proliferative and fibrogenic process considered the central driver of liver fibrosis.^3–5^ Within the inflammatory milieu, transforming growth factor-β (TGFβ) potently stimulates HSC activation, leading to excessive deposition of extracellular matrix (ECM) components.^6,7^ In parallel, hepatocellular stress and death promote the recruitment of macrophage, which secrete hepatokines such as hepatocyte growth factor (HGF) and TGFβ.^6,8^ This intrahepatic crosstalk among hepatocytes, macrophages, and HSCs orchestrates both hepatocyte regeneration and fibrogenesis, positioning the hepatocyte–macrophage–HSC axis as central to metabolic dysfunction-associated steatohepatitis (MASH) and other injury responses.^6,9^ Deciphering the molecular mediators of this network may reveal new therapeutic targets for fibrotic liver disease.

Liver fibrosis is the pathological outcome of chronic liver injury and inflammation. However, regardless of the cause, the final manifestation of liver fibrosis involves excessive deposition of collagen fibers, accumulation of ECM, scar formation, and subsequent insufficient liver blood supply and morphological changes. Therefore, studying the pathogenic mechanisms and treatment options for liver fibrosis holds significant importance in clinical practice and medical research. HSCs are a resident non-parenchymal population of liver cells and the primary fibroblast type responsible for responding to liver injury. In normal liver tissue, HSCs remain quiescent and non-proliferative. Upon liver injury, HSCs become activated into activated HSCs (aHSCs), transdifferentiating from lipid-storing cells into myofibroblast-like cells. These activated cells exhibit proliferative, contractile, and pro-inflammatory properties, with the most critical hallmark being excessive ECM accumulation.^10^ Although extracellular molecular signals such as TGFβ, Galectin-3, CCL2, and CCL5 can activate intracellular signaling pathways, including oxidative stress, autophagy, and metabolic reprogramming, and promote HSCs activation, the molecular mechanisms regulating HSCs activation remain unclear.^11^ The importance of the macrophage–HSC axis has been fully demonstrated in multiple studies and may play a key role in reversing liver fibrosis. In addition, HSCs can also secrete tissue inhibitors of metalloproteinases (TIMPs), which inhibit the degradation of ECM through matrix metalloproteinases (MMPs), thereby making tissue fibers permanent and prolonging liver fibrosis.^12^ The relationship between macrophages and HSCs is an important biomarker for the progression of liver fibrosis. Various cytokines and extracellular matrix components such as collagen further promote fibrosis through HSCs.^13^ Therefore, HSCs has been commonly regarded as a target for anti-fibrotic therapy.

Mallory–Denk bodies (MDBs) are cytoplasmic inclusions within hepatocytes that are observed in diverse liver pathologies, including alcohol-associated steatohepatitis (ASH), liver fibrosis, and hepatocellular carcinoma (HCC).^14,15^ MDB-like inclusions are also present in certain neurodegenerative disorders, such as Alzheimer’s disease and Parkinson’s disease.^16^ MDB formation reflects dysfunction of the 26S proteasome, leading to the accumulation of keratins 8 and 18 (K8/K18) together with undigested proteins such as heat shock proteins (HSPs), ubiquitin (Ub), and Sqstm1/p62 in ballooned hepatocytes.^17–19^ In mice, feeding 3,5-diethoxycarbonyl-1,4-dihydrocollidine (DDC) for ten weeks induces MDB formation in clusters of hepatocytes.^20^ These inclusions largely resolve within one month after DDC withdrawal but rapidly reappear within seven days of DDC refeeding.^17^ MDB-forming hepatocytes exhibit a growth advantage and acquire progenitor cell-like features.^21,22^ Consistently, in human HCC tissues and drug-induced hepatitis models show that MDB-forming hepatocytes express progenitor markers such as ubiquitin D (UbD) and CD49f.^22^ The proinflammatory cytokines TNFα and IFNγ upregulate the expression of UbD, which is essential for MDB biogenesis.^23,24^ Additional pathways, including interferon-stimulated response element (ISRE), NF-κB, and STAT3, also promote UbD expression.^25^ Despite these insights,^19,22^ the influence of liver cell heterogeneity and the potential reprogramming of specific MDB-forming hepatocyte subsets in driving disease progression remain poorly defined.

Recent advances in single-cell and single-nucleus RNA sequencing (scRNA-seq and snRNA-seq) have illuminated the complexity of liver cellular heterogeneity and ligand–receptor interactions in both normal physiology and advanced fibrosis.^8,26–28^ In our prior work, we analyzed global gene expression and signaling pathways in liver biopsies from ASH patients with MDBs and in DDC-induced mouse livers via bulk RNA sequencing and microarrays.^29,30^ Here, we employed snRNA-seq to characterize the transcriptomic architecture of MDB pathogenesis at single-cell resolution and to identify novel intrahepatic crosstalk. Furthermore, we established, for the first time, a 3D organoid model of MDB formation in mouse hepatocytes, which revealed a causal role for HSCs in promoting MDB development. Mechanistically, we identified the HGF/MET/UbD axis as a critical driver of MDB formation that concurrently reduced liver fibrosis, an effect that was markedly attenuated in the UbD⁻/⁻ mice. Collectively, these findings provide new mechanistic insights into how MDBs contribute to hepatic inflammation and fibrosis, and suggest potential therapeutic avenues for chronic liver disease.

## Results

### MDB formation is a heterogeneous pathological process associated with liver fibrosis and the development of cholestatic liver injury

**Fig. 1:**
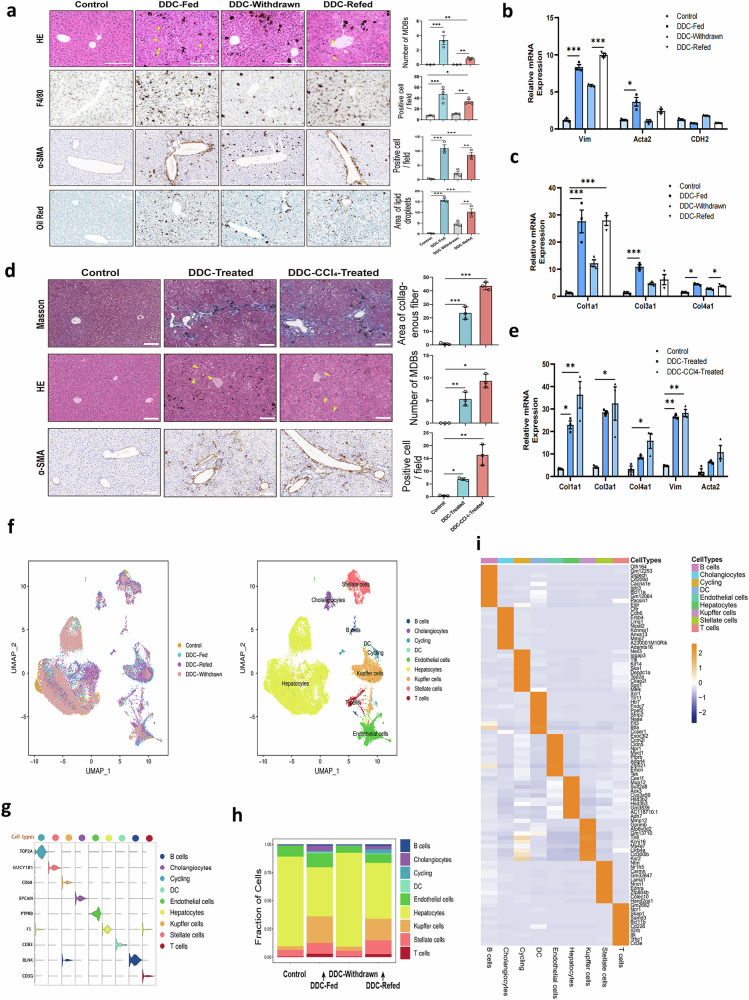
MDB pathogenesis is associated with liver fibrosis and cellular heterogeneity. **a** Liver fibrosis, lipid deposition, and macrophage activation (F4/80) were assessed in the four groups via H&E, Masson, Oil Red O, and α-SMA IHC staining. Brown pigment deposition (black arrow) and MDBs (yellow arrow) were evident in MDB-forming livers. Scale bar: 100 μm. Images were captured at ×20 magnification. Three mice per group were quantified, with 3 fields analyzed per mouse. **b**, **c** RT‒qPCR analysis of fibrosis markers (Acta2, Cdh2, and Vim) and collagen genes (Col1a1, Col3a1, and Col4a1) in liver homogenates. **d** Representative Masson, α-SMA and H&E staining showing fibrosis and MDB formation (yellow arrows). Scale bar: 100 μm; ×20 magnification. Quantification was performed as in (**a**). **e** RT‒qPCR analysis of fibrosis-related genes. **f** UMAP plots of the single-cell atlas from four groups showing nine major cell types categorized by sample origin (top) and cell type identity (bottom). **g** Violin plots showing marker gene expression for the nine cell types. **h** Bar plots of cell type proportions across samples. **i** Heatmap of the top DEGs (Wilcoxon test) for each cell type. The data are presented as the means ± SEMs; n = 3 mice per group. *p < 0.05; **p < 0.01; ***p < 0.001

We established a stable animal model of MDB formation via DDC feeding, following our previously reported protocol.^20,31^ The pathological features of MDB formation were assessed in DDC-treated mouse livers (Supplementary Table 1). DDC-treated mice exhibited marked liver enlargement and elevated serum alanine aminotransferase (ALT), alkaline phosphatase (ALP), and aspartate aminotransferase (AST) levels. Compared with the control conditions, histological analysis revealed prominent ductular reactions, hepatic fibrosis, hepatocyte ballooning, steatosis, and inflammatory infiltration (Supplementary Table 1). Since DDC is a porphyrinogenic drug, we evaluated whether DDC induction causes cholestatic injury. As expected, DDC-treated mice presented prominent pigment deposition (Fig. 1a, Supplementary Table 1), which was consistent with porphyrin accumulation. Immunofluorescence staining revealed the colocalization of K8 and p62 in DDC-treated livers (Supplementary Fig. 1a, b). RT‒qPCR analysis revealed significant upregulation of MDB-related marker expression in DDC-Fed and DDC-Refed livers (Supplementary Fig. 1c).

Immunostaining also confirmed more severe hepatic fibrosis, inflammatory cell infiltration, and lipid accumulation in the DDC-Fed and DDC-Refed groups than in the control groups (Fig. 1a). Notably, F4/80-positive macrophage infiltration, α-SMA positive staining, myofibroblast accumulation, steatosis, and lipid-like deposits were frequently observed around MDB-forming hepatocytes (Fig. 1a). RT‒qPCR analysis of whole-liver homogenates revealed significantly elevated α-SMA (Acta2) expression in MDB mice (Fig. 1b). Consistent with epithelial-to-mesenchymal transition (EMT) associated fibrosis, N-cadherin (CDH2) expression decreased, whereas Vimentin (Vim) expression increased (Fig. 1b). Collagen deposition was also markedly enhanced, with upregulated type I (Col1a1), type III (Col3a1), and type IV (Col4a1) collagen transcripts (Fig. 1c). Interestingly, compared with DDC alone, DDC plus CCl4 treatment further exacerbated MDB formation and hepatic fibrosis, with significantly greater expression of fibrosis-related genes (Fig. 1d, e).

SnRNA-seq generated 35,588 high-quality nuclear transcriptomes across the four groups (6077 Control; 9089 DDC-Fed; 11,791 DDC-Withdrawn; 8631 DDC-Refed), consistent with coverage in recent liver cell-specific transcriptional profiling studies.^32^ After normalization, we performed principal component analysis (PCA) and uniform manifold approximation and projection (UMAP) clustering. Nine major liver cell clusters were identified (Fig. 1f) based on canonical marker genes (Fig. 1g): hepatocytes, endothelial cells, dendritic cells (DCs), cholangiocytes, hepatic stellate cells (HSCs), Kupffer cells (KCs), T cells, B cells, and cycling cells.

The cell-type proportions were similar between the control and DDC-Withdrawn groups, but differed markedly between the DDC-Fed and DDC-Refed groups (Fig. 1h). MDB-forming livers exhibited significantly increased proportions of KCs, HSCs, and cholangiocytes, with a concomitant reduction in hepatocyte frequency, although hepatocytes still accounted for >43% and >49% of the total cells in the DDC-Fed and DDC-Refed groups, respectively (Fig. 1h, Supplementary Fig. 2). These hepatocyte proportions are comparable to those reported in MASH livers.^26^ Differentially expressed genes (DEGs) and marker gene distributions, visualized via t-distributed stochastic neighbor embedding (t-SNE) plots, confirmed the accuracy of the cell type classification (Fig. 1i).

These results confirmed the presence of liver fibrosis and the development of cholestatic liver injury, and revealed marked heterogeneity in MDB pathogenesis during chronic liver injury.

### Emergence of MDB-associated hepatocytes (MAHs) and their molecular signature

**Fig. 2:**
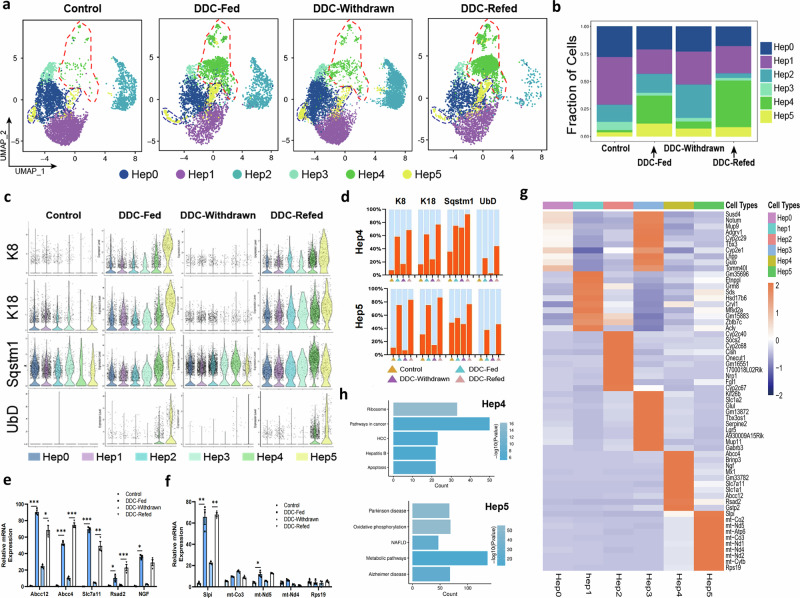
Emergence and molecular signature of MDB-associated hepatocytes (MAHs). **a** UMAP plots showing six hepatocyte subclusters across the control, DDC-Fed, DDC-Withdrawn, and DDC-Refed groups. **b** Bar plots of hepatocyte subcluster proportions in each group. **c** Violin plots of MDB marker gene expression across clusters. **d** Expression patterns of K8, K18, Sqstm1, and UbD in Hep4 (top) and Hep5 (bottom) cells across groups. **e**, **f** RT‒qPCR validation of the expression of selected genes in Hep4 and Hep5 cells from DDC-treated versus control livers. **g** Heatmap of the top 10 DEGs (Wilcoxon test) across hepatocyte clusters. **h** KEGG enrichment of DEGs in Hep4 (top) and Hep5 (bottom) cells; adjusted *p* < 0.05. The data are shown as the means ± SEMs (n = 3). *p < 0.05; **p < 0.01; ***p < 0.001

We next re-clustered hepatocytes via UMAP analysis and identified six major subclusters (Hep0–Hep5) based on canonical hepatocyte markers (Fig. 2a). The distributions of these subclusters across the four experimental groups are shown in Supplementary Fig. 3. Compared with those in the control and DDC-Withdrawn groups, the proportions of Hep1 and Hep3 cells in the DDC-Fed and DDC-Refed group were significantly lower (Fig. 2b, Supplementary Fig. 4). In contrast, the Hep4 and Hep5 proportions markedly increased in DDC-Fed and DDC-Refed livers (Fig. 2b, Supplementary Fig. 4).

Hep0 expressed high levels of Tomm40l, Gulo, Lhpp, Cyp2e1, Tbx3, Cyp2c29, Adgrv1, Mup9, Notum, and Susd4, with GO and KEGG analyses indicating enrichment in metabolic pathways, biosynthesis, and bile secretion (Supplementary Fig. 5). Hep1, Hep2, and Hep3 cells mainly exhibited activation of basal metabolism, PPAR signaling, and complement cascades (Supplementary Fig. 6), suggesting shared transcriptional regulation. The origins and functions of these subclusters warrant further investigation.

The Hep4 proportion increased dramatically, from 2.12% and 6.44% in the control and DDC-Withdrawn groups to 25.50% and 42.36% in the DDC-Fed and DDC-Refed groups, respectively (Fig. 2b, Supplementary Fig. 4). Notably, Hep5 also expanded suggesting that it may represent another progenitor subtype associated with MDB formation. Violin plots revealed that Hep5 and Hep4 had the highest and second-highest expression of K8, K18, and UbD, respectively, and the highest and second-highest expression of Sqstm1 in the DDC-treated groups (Fig. 2c). Across all clusters, the proportion and number of MDB marker-expressing cells were significantly greater in Hep4 and Hep5 cells than in control cells (Fig. 2d). The K8^high^ population originated predominantly from MDB livers, comprising 58.78% and 69.10% of Hep4 and 75.97% and 83.74% of Hep5 in the DDC-Fed and DDC-Refed groups, respectively (Fig. 2d). These data define Hep4 and Hep5 cells as distinct hepatocyte subpopulations involved in MDB pathogenesis, hereafter termed MDB-associated hepatocytes (MAHs).

MAHs presented high expression of Slc7a11 and NGF during MDB pathogenesis (Fig. 2e). Hep5 was further characterized by elevated Slpi, ribosomal protein Rps19, and mitochondrial DNA (mtDNA)-encoded genes (mt-Co2, mt-Nd5, mt-Atp6, mt-Co3, mt-Nd1, mt-Nd4, mt-Nd2, and mt-Cytb) (Fig. 2f, g). Notably, the total mtDNA content was greater in the DDC-Fed and DDC-Refed groups than in the control groups (Supplementary Fig. 7a, b), implicating that mtDNA depletion is a potential biomarker for drug-induced liver injury and MDB-associated chronic liver disease.

Pathway analysis revealed distinct functional reprogramming between the two MAH subtypes: Hep4 cells were enriched for cancer, HCC, and apoptosis pathways, whereas Hep5 cells were associated with metabolic processes, oxidative phosphorylation, and neurodegenerative disease pathways, including Alzheimer’s disease and Parkinson’s disease (Fig. 2h). Collectively, these findings highlight that MDB-associated hepatocytes undergo profound molecular and functional remodeling during MDB pathogenesis.

### MAH subsets are closely associated with HCC progression

**Fig. 3:**
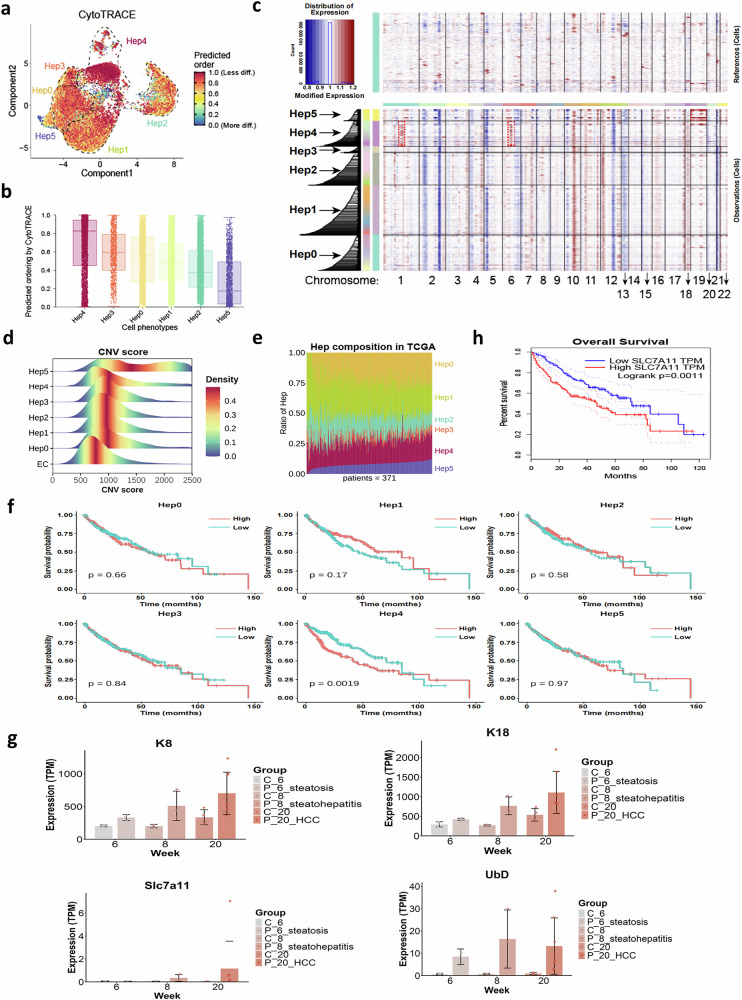
The MAH subset is closely associated with HCC progression. **a**, **b** CytoTRACE analysis showing the differentiation states of hepatocyte subsets, ordered from mature (low values) to immature (high values). **c**, **d** CNV profiles and distribution scores inferred by InferCNV across hepatocyte subsets and endothelial cells. **e** Hepatocyte subset composition (Hep0–Hep5) in HCC patients (TCGA-LIHC). **f** Kaplan–Meier survival analysis of hepatocyte subsets. **g** Expression of K8, K18, Slc7a11, and UbD across liver disease stages in a public database. **h** Kaplan–Meier survival analysis of Slc7a11 expression in TCGA-LIHC cohort

To investigate the developmental potential and differentiation status of hepatocyte subsets during chronic liver disease, we performed CytoTRACE analysis. The MDB-associated subset Hep5 displayed a highly differentiated state, whereas Hep4 displayed the highest differentiation potential, suggesting its potential contribution to malignant behavior (Fig. 3a, b).

Genomic heterogeneity analysis via InferCNV, with endothelial cells as the reference, revealed that Hep0–Hep5 cells presented broadly similar CNV patterns. However, Hep4 exhibited prominent CNV gains on chromosomes 1 and 6, whereas Hep5 showed marked heterogeneity with a distinct CNV gain on chromosome 19 (Fig. 3c). Using UPhyloplot2 to analyze clonal evolution on the basis of InferCNV results, we identified specific CNV events—including chr1p_gain, chr6p_gain, chr19p_gain, and chr19q_gain—that demonstrated nonrandom distribution in the DDC-treated groups (Supplementary Fig. 8). These events consistently emerged as early, concentrated “trunk” events in the phylogenetic tree. CNV score analysis indicated that Hep4 cells had a slightly higher score than Hep0–Hep3 cells did, whereas Hep5 cells had the highest score, reflecting its pronounced genomic instability (Fig. 3d). These CNV alterations likely contributed to the heterogeneity observed in MDB-related subsets.

To assess clinical relevance, we performed deconvolution analysis on bulk RNA-seq data from the TCGA-LIHC cohort via Bisque, which is based on hepatocyte expression profiles derived from snRNA-seq, to resolve the hepatocyte subpopulation composition (Fig. 3e). Survival analysis revealed that a greater proportion of Hep4 cells was correlated with poorer prognosis, a finding validated in the LIRI cohort (Fig. 3f, Supplementary Fig. 9a). Thus, Hep4 cells appear to be associated with unfavorable outcomes, whereas Hep5 cells are characterized by substantial transcriptional heterogeneity.

Expression analysis of MDB marker genes (K8, K18, UbD, and Slc7a11) in the GSE83596 (mouse) and GSE114564 (human) datasets revealed elevated expression levels in steatosis, steatohepatitis, and HCC (Fig. 3g). Notably, K8, Sqstm1, Slc7a11, and UbD were significantly upregulated in advanced HCC, with Slc7a11 expression uniquely restricted to advanced cases (Supplementary Fig. 9b). To investigate the significance of MDB-related genes in human fatty liver disease, we utilized human MASH-related snRNA-seq data from GSE202379 and analyzed the changes in the expression of MDB-related genes in hepatocytes across different stages of fatty liver disease. Our results revealed that K8/Sqstm1 and UbD were gradually upregulated with the progression of liver disease, whereas Slc7a11 was an exception; it was highest in healthy liver tissue but subsequently increased with the worsening of liver disease (Supplementary Fig. 9c). K‒M analysis of Hep4-derived genes in the TCGA-LIHC cohort revealed that high Slc7a11 and K8 expression was significantly associated with reduced overall survival (Fig. 3h, Supplementary Fig. 10). Immunostaining further confirmed that stronger cytoplasmic Slc7a11 signals colocalized with p62 in ballooned hepatocytes containing MDBs in HCC biopsies (Supplementary Fig. 11a, b). We further explored hepatocyte differentiation trajectories following DDC-induced chronic liver injury, which confirmed that MDB cells are hepatocyte-derived and that liver injury alters hepatocyte differentiation trajectories toward a pathological, cancer-related fate (Supplementary Figs. 12 and 13).

Collectively, these findings suggest that MDB-related hepatocyte subsets, particularly MAHs, exhibit distinct developmental potential and genomic instability that contribute to HCC progression. Slc7a11 has emerged as a potential prognostic biomarker for advanced HCC.

### snRNA-seq reveals distinct HSC subpopulations involved in MDB formation

**Fig. 4:**
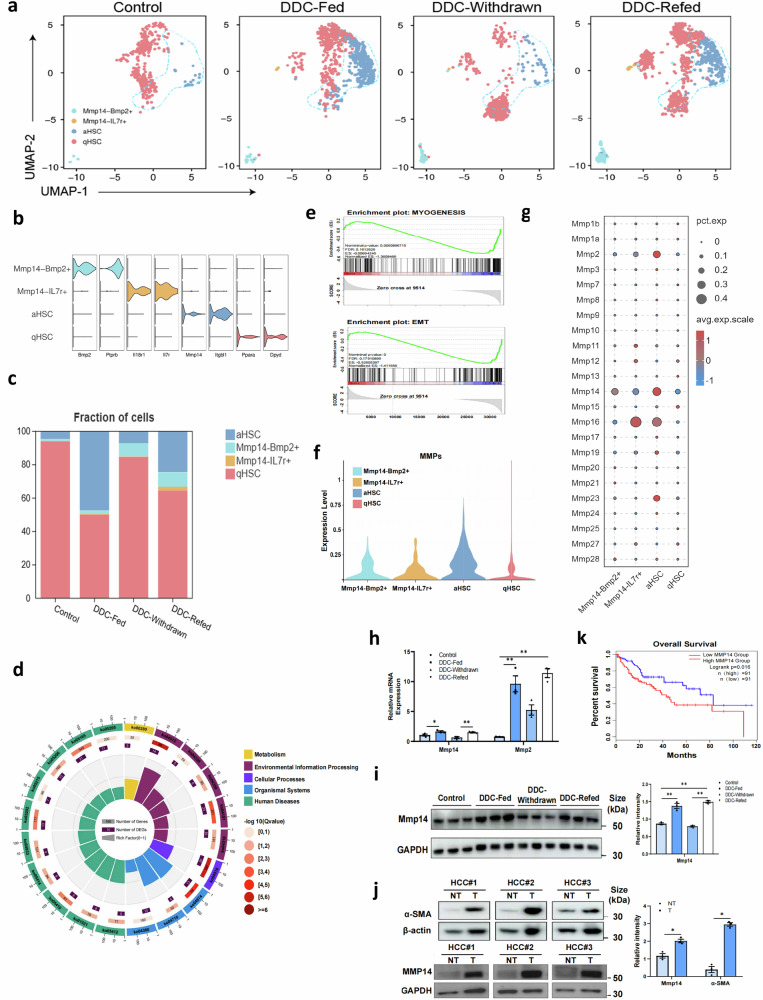
Single-cell analyses identify distinct HSC populations in MDB livers. **a** UMAP visualization showing aHSC, qHSC, Mmp14-Bmp2⁺, and Mmp14-Il7r⁺ subpopulations in control, DDC-Fed, DDC-Withdrawn, and DDC-Refed livers. **b** Violin plots of marker genes for each subcluster. **c** Contribution of each group to the HSC subclusters. **d** KEGG circular plot of enriched pathways in aHSCs from DDC-Fed livers. **e** GSEA of EMT and myogenesis in aHSCs versus other clusters. **f**, **g** Violin plots and bubble maps of Mmp gene expression across HSC subsets. **h** RT‒qPCR validation of Mmp14 and Mmp2 expression in control vs DDC-treated livers. **i** Western blot analysis of Mmp14 in DDC and control mice. **j** Western blot of MMP14 and α-SMA in paired tumor and nontumor tissues from HCC patients. **k** Kaplan–Meier survival analysis of LIHC patients stratified by Mmp14 expression (TCGA, GEPIA). The data are shown as the means ± SEMs (n = 3). *p < 0.05; **p < 0.01; ***p < 0.001

To elucidate the mechanisms driving hepatic MDB pathogenesis, we re-clustered HSC subpopulations and identified four distinct clusters via UMAP analysis, namely, activated HSCs (aHSCs), quiescent HSCs (qHSCs), Mmp14-Bmp2⁺, and Mmp14-IL7r⁺, on the basis of gene expression profiles (Fig. 4a). The endothelial-associated cluster, characterized by Bmp2 and Ptprb expression, may play a role in endothelial metabolism and vascular tone regulation, whereas the inflammation-associated cluster exhibited IL18r1 and IL7r expression. The aHSC cluster, which is consistent with fibrosis exacerbation, showed high expression of Itgbl1 and the EMT-related gene Mmp14 (Fig. 4b).

Notably, both the number and proportion of aHSCs and Mmp14-IL7r⁺ cells were significantly increased in the DDC-Fed and DDC-Refed groups (Fig. 4c, Supplementary Fig. 14), indicating the activation of HSCs and immune-related cells during MDB pathogenesis. KEGG pathway analysis revealed that the aHSC cluster was enriched in ECM–receptor interactions, focal adhesion, and inflammatory pathways (e.g., PI3K–AKT and TGFβ) (Fig. 4d). Hallmark gene set analysis further highlighted the upregulation of the EMT and myogenesis programs (Fig. 4e). A key feature of EMT, loss of epithelial integrity via adhesion junction degradation, is largely mediated by matrix metalloproteinases (MMPs). Indeed, the Mmp genes were predominantly enriched in the aHSC cluster (Fig. 4f), with Mmp2, Mmp3, Mmp14, Mmp17, Mmp19, Mmp23, and Mmp24 markedly upregulated in aHSCs, and Mmp11, Mmp12, and Mmp16 elevated in Mmp14-IL7r⁺ cells (Fig. 4g). RT‒qPCR confirmed the significant induction of Mmp14 and Mmp2 in DDC-treated mice (Fig. 4h), and the MMP14 protein levels were similarly elevated (Fig. 4i).

To explore the clinical relevance of these findings, we examined MMP14 and α-SMA in tumor and nontumor tissues from HCC patients with MDBs. Both proteins were markedly increased in tumor tissues (Fig. 4j), and Kaplan–Meier analysis (GEPIA2) showed that high MMP14 expression was associated with significantly shorter overall survival (Fig. 4k). Public datasets (GSE83596, mouse; GSE162694, human) confirmed that K8, Sqstm1, UbD, and Mmp14 were all upregulated in fibrosis (Supplementary Fig. 15a, b). Immunohistochemistry further confirmed elevated Mmp14 and α-SMA levels in cancerous versus paracancerous tissues (Supplementary Fig. 15c, d).

Collectively, these results demonstrate that HSCs undergo functional reprogramming during MDB pathogenesis, with Mmp14 emerging as a potential biomarker for liver fibrosis and a prognostic indicator in HCC.

### The HGF/MET axis in hepatocyte–macrophage–HSC interactions

**Fig. 5:**
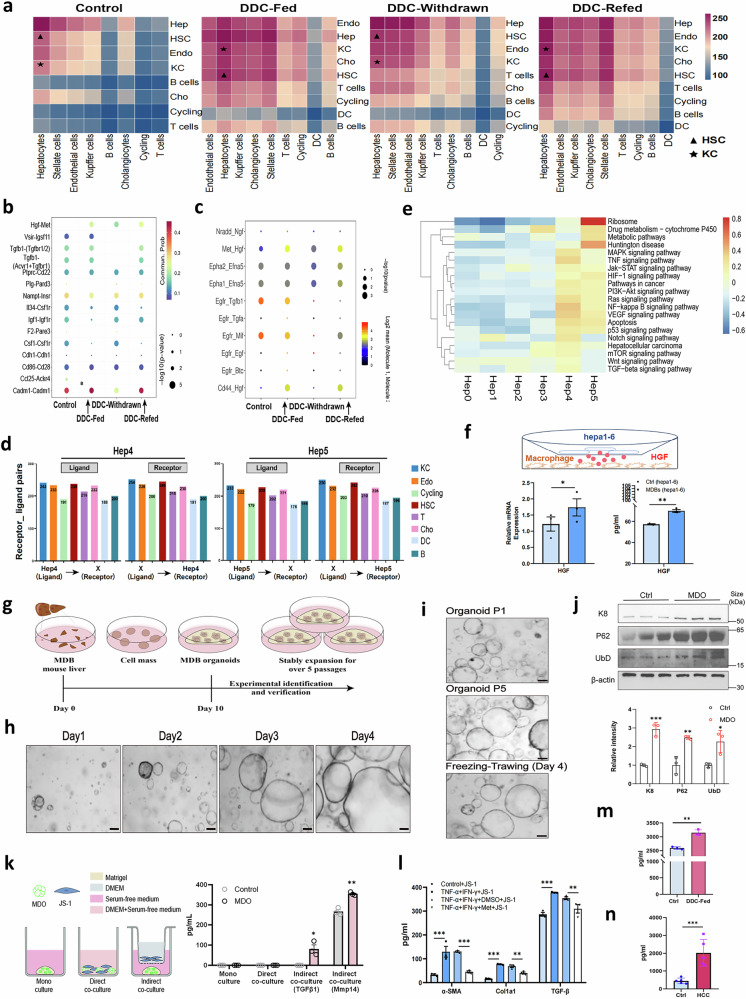
Enrichment of the HGF/MET axis during MDB pathogenesis. **a** Heatmap of ligand–receptor interactions between hepatocytes and HSCs/KCs in DDC-Fed and DDC-Refed livers. **b**, **c** Dot plots of enriched ligand‒receptor interactions between hepatocytes and KCs (**b**) or aHSCs (**c**). **d** Bar plot of receptor–ligand interactions between Hep4/Hep5 cells and the indicated cell types. **e** GSVA of enriched pathways in hepatocyte subclusters. **f** RT‒qPCR and ELISA results showing HGF expression in Raw264.7 cells cocultured with MDB-forming Hepa1-6 cells. **g** Experimental schematic of mouse MDB organoid (MDO) cultures. **h**, **i** Representative images of DDC-induced MDOs across time points (**h**) and passages (**i**). Scale bar: 100 μm. **j** Western blot of K8, UbD and p62 in organoids from control vs DDC-Fed mice. **k** ELISA of TGFβ1 and Mmp14 in coculture supernatants from direct and indirect MDO–JS-1 coculture systems. **l** ELISA analysis of TGFβ1, α-SMA, and Col1a1 secretion in coculture supernatants. **m**, **n** ELISA of HGF secretion in mouse serum (n = 3) and HCC samples (n = 5). The data are shown as the means ± SEMs (n = 3 or 5). *p < 0.05; **p < 0.01; ***p < 0.001

To investigate cell–cell communication during MDB pathogenesis, we analyzed intercellular interactions on the basis of ligand–receptor pairs. As expected, hepatocytes exhibited significant ligand–receptor interactions with HSCs and KCs in DDC-treated livers (Fig. 5a). HSCs also exhibited a robust signaling network with hepatocytes, macrophages, and cholangiocytes in MDB-forming livers (Fig. 5a), consistent with their role as hubs for intrahepatic signaling through stellakine secretion.^26^ Notably, the hepatocyte growth factor (HGF)/mesenchymal–epithelial transition factor (MET) pair was highly enriched in hepatocyte interactions with both KCs (Fig. 5b) and HSCs (Fig. 5c) in DDC-treated mouse livers.

Because hepatocyte ballooning and MDB formation are hallmarks of ongoing inflammation, we further examined the interactions of the hepatocyte subpopulation with KCs. KCs in DDC-Fed mice expressed more ligands that target the receptors on MAH subpopulations Hep4 and Hep5 than did those in control mice (Fig. 5d), suggesting a key role for KCs in MDB formation, which is in line with our recent findings.^31^ Both HGF and MET were significantly induced and evenly expressed in KCs and hepatocyte subsets, respectively (Supplementary Fig. 16). Downstream activation signatures of the HGF/MET axis, such as PI3K/AKT and NF-κB signaling, were significantly upregulated in the Hep4 and Hep5 subpopulations of DDC-treated livers, as revealed by GSVA (Fig. 5e). These results suggest that aberrant HGF/MET signaling is closely linked to MDB pathogenesis.

To explore macrophage–hepatocyte crosstalk, we cocultured Raw264.7 macrophages with MDB-forming Hepa1-6 cells pretreated with TNFα and IFNγ. HGF mRNA expression was markedly increased in macrophages after coculture with MDB-forming Hepa1-6 cells (Fig. 5f, left). ELISA confirmed a significant rise in HGF levels in the culture medium when macrophages were directly cocultured with MDB-forming Hepa1-6 cells (Fig. 5f, right), indicating that MDB-forming hepatocytes activate macrophages to secrete HGF.

To further dissect HGF/MET-mediated mechanisms, we established MDB organoids (MDOs) from DDC-induced mouse livers. The cell aggregates embedded in 3D Matrigel formed hollow spheres within two days and expanded into large 3D organoids over the next 10 days, which could be passaged every 7–9 days at a 1:2–1:4 ratio for >5 passages (Fig. 5g). Organoids grew rapidly after passage (Fig. 5h) and retained viability after freezing–thawing (Fig. 5i). Western blot analysis confirmed the high expression of the MDB markers K8, UbD and p62 in these organoids compared with that in the organoids derived from non-DDC normal liver tissues (Fig. 5j).

Given that prior KEGG analysis showing TGFβ pathway activation in aHSCs, we tested whether MDOs secrete factors that affect HSCs. JS-1 HSCs were cocultured with normal or MDB organoids in both direct and indirect systems to distinguish contact-dependent effects from soluble factor-mediated effects (Fig. 5k). ELISAs revealed increased TGFβ1 and Mmp14 secretion in the indirect coculture setup when JS-1 HSCs were cocultured with MDB organoids (Fig. 5k). To further assess hepatocyte-to-HSC crosstalk, conditioned media from MET-inhibited MDB-forming Hepa1-6 hepatocytes were applied to JS-1 HSCs. ELISAs revealed a significant increase in TGFβ1, α-SMA, and Col1a1 secretion in the coculture system (Fig. 5I), confirming that MDB-forming hepatocytes secrete profibrotic factors that activate HSCs. Finally, serum HGF levels were measured in DDC-treated mice and HCC patients with MDBs. As expected, HGF was significantly elevated in both DDC-Fed mouse (Fig. 5m) and HCC patient samples compared with normal liver tissues samples (n = 5; Fig. 5n).

Collectively, these findings demonstrate that the HGF/MET axis is a key signaling pathway enriched in hepatocyte–KC and hepatocyte–HSC interactions and likely plays a central role in MDB pathogenesis.

### The HGF/MET axis mediates liver MDB formation via NF-κB and STAT3 signaling

**Fig. 6:**
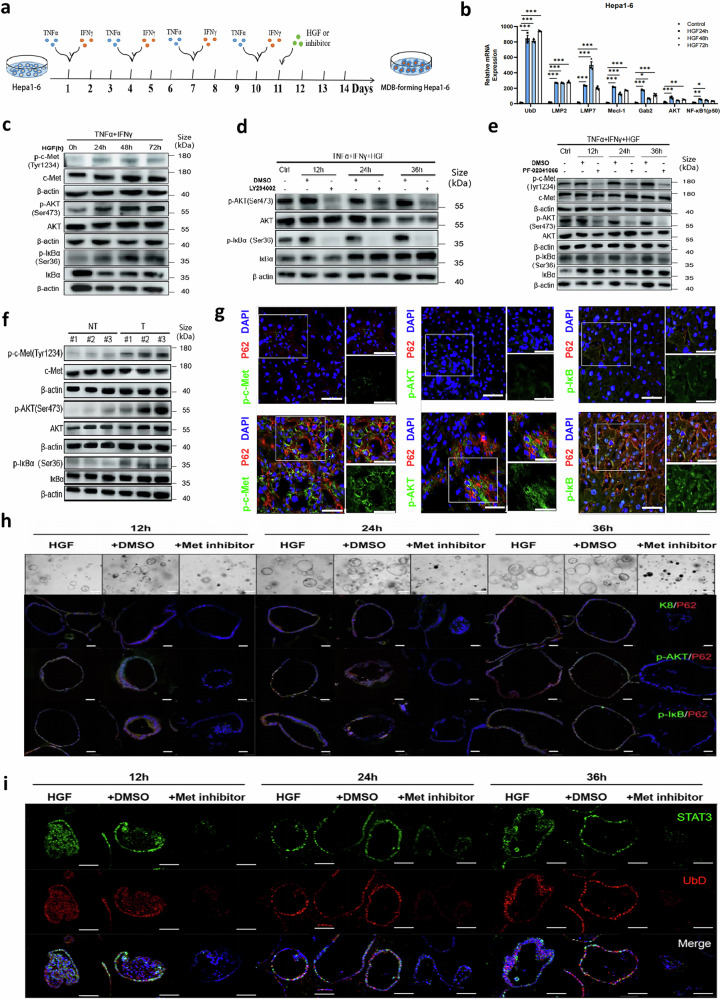
The HGF/MET axis drives MDB formation via NF-κB and STAT3 signaling. **a** Experimental design schematic. Hepa1-6 cells were stimulated with TNFα (40 ng/mL) and IFNγ (400 ng/mL) at three-day intervals, and the cells were collected for assays. **b** mRNA expression in Hepa1-6 cells at 24, 48, and 72 h after HGF (20 ng/mL) treatment. **c** Western blot analysis of total and phosphorylated MET, AKT, and IκBα in MDB-forming Hepa1-6 cells after HGF treatment. **d**, **e** Western blot analysis of PI3K/AKT/NF-κB signaling in Hepa1-6 cells treated with HGF in the presence/absence of a PI3K inhibitor (LY294002) or MET inhibitor (PF-02341066). **f** Western blot analysis of signaling proteins in MDB-HCC tissues. **g** Immunofluorescence costaining of p-AKT, p-MET, and p-IκBα with p62 in MDB-HCC versus nontumor tissues (n = 5). Scale bar: 50 μm. **h** Bright-field and immunofluorescence imaging of MDOs treated with the MET inhibitor revealed that K8/p-AKT/p-IκBα colocalized with p62. Scale bars: 200 μm (bright field) and 50 μm (fluorescence). **i** Immunofluorescence imaging of STAT3 and UbD colocalization in MDOs treated with MET inhibitors. Scale bars: 100 μm (fluorescence). The data are shown as the means ± SEMs (n = 3 or 5). *p < 0.05; **p < 0.01; ***p < 0.001

In our recent work,^31^ we established an MDB-forming Hepa1-6 cell model by treating cells with TNFα and IFNγ (Fig. 6a) to examine whether the HGF/MET axis contributes to liver MDB progression. Immunocytochemical staining revealed increased cytoplasmic intensity of K8/p62 in Hepa1-6 cells that had formed MDBs (Supplementary Fig. 17). HGF treatment significantly upregulated the mRNA expression of UbD, LMP2, LMP7, MECL-1, Gab2, AKT, and NF-κB1 (p50) in MDB-forming Hepa1-6 cells compared with that in untreated cells (Fig. 6b).

To investigate the underlying mechanisms, we assessed PI3K/AKT/NF-κB pathway activation. HGF treatment induced the phosphorylation of MET (p-MET) and AKT (p-AKT) in MDB-forming Hepa1-6 cells and increased the phosphorylation of IκBα (p-IκBα), a key NF-κB signaling component, which peaked at 72 h after treatment (Fig. 6c, Supplementary Fig. 18a).

Inhibition experiments confirmed the role of the PI3K/AKT/NF-κB pathway in MDB formation. The levels of phosphorylated PI3K/AKT/NF-κB signaling in Hepa1-6 cells treated with HGF in the presence/absence of a PI3K inhibitor (LY294002) or MET inhibitor (PF-02341066) lower than those in HGF + DMSO control cells (Fig. 6d, e; Supplementary Fig. 18b). Consistent with these results, elevated p-MET, p-AKT, and p-IκBα levels were markedly increased in HCC patients with MDBs compared with nontumor tissues (Fig. 6f, Supplementary Fig. 18c). Immunostaining confirmed strong cytoplasmic staining of p-MET, p-AKT, and p-IκBα in ballooned hepatocytes containing MDBs from five different HCC patients (Fig. 6g, Supplementary Fig. 18d).

In MDB organoids, MET inhibition delayed proliferation, reduced organoid size, and caused cell disintegration (Fig. 6h, top). Immunostaining revealed decreased expression of p-AKT, p-IκBα, K8, and p62 under MET inhibition (Fig. 6h, bottom), supporting a key role for HGF/MET signaling in MDB pathogenesis within a 3D culture context.

Given the established role of STAT3 in inflammation, fibrosis and cancer progression, we next examined its involvement. In the Hepa1-6 model, HGF treatment induced time-dependent increases in UbD and p-STAT3 expression, which were suppressed upon MET inhibition (Supplementary Fig. 18e). Similarly, in MDB organoids, MET inhibition revealed significant downregulation expression of STAT3 and UbD levels (Fig. 6i).

Collectively, these findings demonstrate that the HGF/MET axis drives MDB pathogenesis through the activation of the NF-κB- and STAT3-mediated signaling pathways.

### UbD deficiency inhibits HGF/MET signaling and liver fibrosis

Using the single-cell regulatory network inference and clustering (SCENIC) method, we observed marked upregulation of the transcription factors NF-κB1 (p50) and Jun in the Hep4 subcluster (Supplementary Fig. 19a) and in DDC-treated liver tissues across all four groups (Supplementary Fig. 19b). NF-κB mediates proinflammatory responses, whereas AP-1 regulates growth responses, both of which are implicated in MDB formation under DDC-induced conditions in vivo and in primary hepatocyte cultures.^25^

To verify whether HGF/MET signaling drives the proinflammatory response, we measured key inflammasome components in DDC-treated liver tissues. DDC markedly increased TNFα, IFNγ, caspase-1, and IL-1β expression, whereas DDC withdrawal or a normal diet prevented these increases (Supplementary Fig. 20a). The expression of ERK1, an upstream activator of AP-1 and cell proliferation, was also upregulated in DDC-treated mice (Supplementary Fig. 20b). These results indicate that HGF/MET signaling activates both proinflammatory and growth pathways, which are potentially amplified by TNFα and IFNγ via NF-κB activation.

We next examined UbD expression in MDB-forming Hepa1-6 cells. Compared with that in untreated controls, the level of UbD was strongly induced (Supplementary Fig. 20c) and was similarly elevated in mouse liver tissues (Supplementary Fig. 20d) and in the livers of HCC patients with MDBs (Supplementary Fig. 20e). To determine whether NF-κB regulates UbD transcription, we found that DDC treatment significantly increased the phosphorylation of p50 (p-p50) in the MDBs of mouse livers (Supplementary Fig. 20f). Bioinformatic analysis identified three putative NF-κB1 (p50) binding sites in the UbD promoter (Supplementary Fig. 20g, Supplementary Table 4), as well as additional binding sites in the promoters of TNFα (n = 4) and IFNγ (n = 1). We also identified three high-scoring STAT3-binding sites in the upstream UbD promoter (Supplementary Fig. 20g), supporting STAT3-mediated transcriptional regulation. These findings suggest that elevated HGF/MET signaling induces a proinflammatory response through NF-κB and STAT3 activation, leading to UbD upregulation and promoting liver MDB formation.

**Fig. 7:**
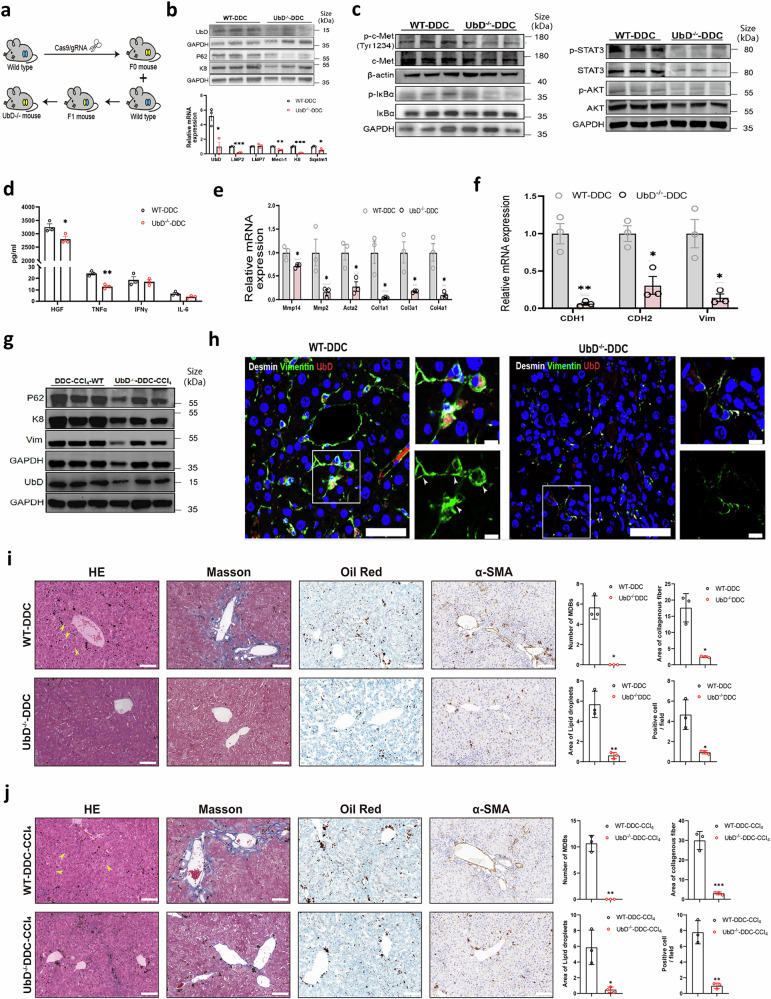
UbD deletion suppresses MDB formation and fibrosis. **a** Schematic of the generation of UbD knockout (UbD⁻/⁻) mice via CRISPR/Cas9. **b** Western blot (top) and RT‒qPCR (bottom) analyses of UbD, p62, K8, and proteasome-related genes in WT-DDC vs UbD⁻/⁻-DDC mice. **c** Western blot analysis of the HGF/MET/NF-κB and STAT3 pathways in WT vs UbD⁻/⁻ mice. **d** ELISA analysis of HGF, TNFα, IFNγ, and IL-6 secretion in WT and UbD⁻/⁻ mice after 8 weeks of DDC treatment (n = 3). **e**, **f** RT‒qPCR of fibrosis markers (Mmp14, Mmp2, Acta2, Col1a1, Col3a1, and Col4a1) and EMT markers (Cdh1, Cdh2, and Vim) in WT and UbD⁻/⁻ mice. **g** The expression of MDB and the fibrosis-related molecule vimentin was detected by Western blot in the UbD^-^/^-^-/DDC-CCl_4_ mice. **h** Immunofluorescence showing colocalization of Vimentin and UbD (white arrows) in the UbD⁻/⁻ and WT mice. Scale bar: 50 μm; zoom: 10 μm. **i** H&E, Masson, Oil Red O and α-SMA immunostaining showing reduced MDB formation (yellow arrows), fibrosis, and lipid accumulation in the UbD⁻/⁻-DDC mice. Scale bar: 100 μm. **j** Histological analysis of liver sections from DDC-CCl₄-induced fibrosis models (WT vs UbD⁻/⁻). Scale bar: 100 μm. The data are shown as the means ± SEMs (n = 3). *p < 0.05; **p < 0.01; ***p < 0.001

We next generated a UbD-knockout (UbD⁻/⁻) mouse model via the CRISPR/Cas9 system (Fig. 7a) to examine the role of UbD in MDB formation and liver fibrosis. The effects of UbD deficiency on MDB formation were assessed at 8 and 10 weeks after DDC administration (n = 3 per group). Hepatic UbD deletion was confirmed in the UbD⁻/⁻ mice, which showed markedly reduced DDC-induced activation of UbD (Fig. 7b, Supplementary Fig. 21a). Similarly, the mRNA levels of UbD, LMP2, LMP7, MECL-1, K8, and p62 were consistently downregulated in the UbD⁻/⁻ livers at 8 weeks post-DDC (Fig. 7b, bottom). At 10 weeks, K8 and UbD protein and mRNA levels were significantly decreased in the UbD⁻/⁻ mice (Supplementary Fig. 21b, c). Moreover, the expression of proapoptotic genes was significantly reduced in the UbD⁻/⁻ mice at 8 weeks (Supplementary Fig. 21c).

We next explored whether UbD deficiency altered DDC-induced changes in the HGF/MET/NF-κB and STAT3 signaling pathways. UbD deletion significantly decreased c-Met and IκB levels but did not affect STAT3 or AKT levels in wild-type (WT) and UbD⁻/⁻ mice (Fig. 7c, Supplementary Fig. 21d, e). Inflammatory analysis revealed that HGF secretion and the proinflammatory cytokine TNFα were significantly lower in the UbD⁻/⁻ livers than in the WT-DDC livers (Fig. 7d), whereas IFNγ and IL-6 levels were unaffected (*P* > 0.05).

To determine the relationship between UbD⁻/⁻ deficiency and fibrosis, we evaluated liver fibrosis in WT and UbD⁻/⁻ mice. Whole-liver homogenates from the UbD⁻/⁻ mice presented reduced expression of fibrosis-related genes (Fig. 7e, f). Additionally, the levels of UbD, K8 and the fibrosis-related molecule Vimentin were decreased in the UbD⁻/⁻–DDC–CCl₄ mice (Fig. 7g, Supplementary Fig. 21f). Moreover, immunostaining further demonstrated decreased Vimentin expression in hepatic stellate cells (marked by Desmin) from the UbD⁻/⁻ mice (Fig. 7h), indicating that UbD promotes fibrosis progression by regulating HSC activation and epithelial–mesenchymal transition (EMT). Histological analysis revealed that MDBs, extensive myofibroblast accumulation, fat droplet deposition around hepatic blood vessels and α-SMA immunostaining were prominent in WT-DDC mice but markedly diminished in UbD⁻/⁻–DDC mice (Fig. 7i). Notably, these features were further reduced in the UbD⁻/⁻–DDC–CCl₄ mice compared with the WT–DDC–CCl₄ mice (Fig. 7j).

Collectively, these findings indicate that UbD deletion attenuates hepatocellular inflammation, fibrosis, and steatosis under fibrotic conditions.

## Discussion

In this study, we employed snRNA-seq to comprehensively delineate the hepatic transcriptomic landscape and revealed novel cellular interactions among hepatocytes, macrophages, and HSCs at single-cell resolution. During chronic liver injury, cholangiocytes can expand and act as liver progenitor cells (LPCs), contributing to hepatocyte regeneration when hepatocyte proliferation is impaired.^33,34^ Therefore, the significant increase in cholangiocyte proliferation in the DDC-Fed and DDC-Refed groups might indicate that cholangiocytes are activated to promote tissue repair and liver regeneration because cholangiocytes can expand and act as LPCs. Previously, we reported that macrophage activation and high IL-7R expression are involved in MDB formation.^31^ In MASH, the hepatocyte–macrophage–HSC network is the most important driver of fibrosis.^6^ An important feature of our snRNA-seq data is that macrophages and HSCs were markedly enriched and expanded following DDC treatment, suggesting that hepatocytes impair fibrosis development during MDB pathogenesis. While HSCs are well characterized as executors of fibrogenesis, how HSCs affect other aspects of MASH and MDB formation is not known. Activated HSCs in liver fibrosis models have been reported to exhibit increased heterogeneity.^35^ Single-cell analysis of HSCs during MDB formation associated with liver fibrosis has not been reported. We revealed a significant expansion of aHSC clusters expressing genes related to inflammation and ECM production. aHSCs undergo epithelial‒mesenchymal transition (EMT), where they differentiate into myofibroblasts that secrete ECM components that contribute to fibrosis.^36^ Among the aHSCs, the MMPs family was enriched, with Mmp14 emerging as a key mediator of the HSC-to-myofibroblast transition during MDB formation, although the precise mechanisms warrant further investigation.

HSCs are a main source of HGF, and the role of HSC-derived HGF has not yet been studied. The HGF receptor MET plays an essential role in hepatocyte regeneration and survival in various liver diseases.^37^ We found that the HGF/MET axis is highly enriched in interactions between Hep4/Hep5 cells and KCs or HSCs. Aberrant HGF/MET signaling activates multiple pathways, including PI3K/AKT/NF-κB^38,39^ and STAT3 pathways,^40^ with NF-κB playing a central role in the proinflammatory responses that drive MDB formation.^25,41^ The phosphorylation of NF-κB promotes the release of cytokines such as TNFα, IL-6, and IL-1β, contributing to liver injury.^42^ In a 3D MDB organoid model, costaining of K8/p-AKT/p-IκB with p62 was markedly reduced upon MET inhibition, demonstrating that HGF/MET signaling promotes MDB formation via the STAT3 and NF-κB pathways. Notably, MDB organoids activated HSCs through unidirectional TGFβ1 secretion, while aHSCs in turn secreted HGF, further promoting pathological MDB formation.

**Fig. 8:**
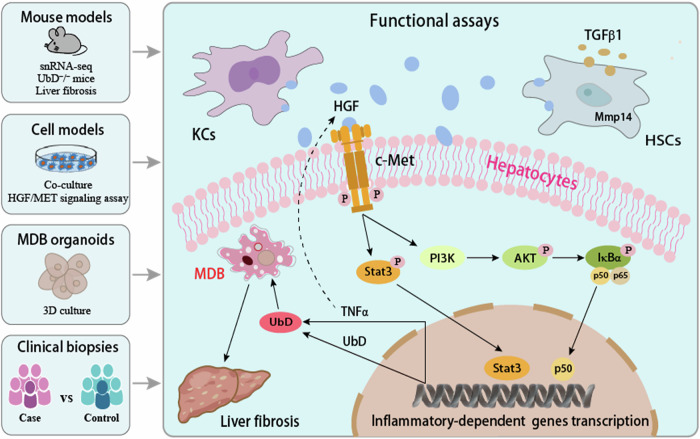
Schematic diagram of the study. Functional in vitro and in vivo experiments demonstrated that, upon injury, HGF interacts with c-Met, activating the PI3K/AKT/NF-κB and STAT3 pathways and promoting UbD upregulation and the release of the proinflammatory cytokine TNFα, which leads to MDB pathogenesis and fibrosis development

UbD is highly upregulated by TNFα and IFNγ, with these cytokines inducing UbD expression via the NF-κB and/or STAT3 pathways.^43,44^ Indeed, UbD overexpression in ASHs promotes balloon degeneration and MDB aggregation, effects that are prevented in UbD⁻/⁻ mice, as previously demonstrated.^24^ Functional assays further demonstrated that HGF treatment, which is induced by TNFα/IFNγ, significantly increases UbD expression. Our findings suggest that, upon chronic liver injury, HGF secreted by KCs or HSCs interacts with MET on balloon hepatocytes, stimulating downstream gene expression, hepatocyte growth and proliferation, and NF-κB activation via protein phosphorylation. This activation leads to the release of inflammatory factors, including TNFα, which further promotes HGF transcription, establishing a positive feedback loop. TNFα-induced UbD expression, driven by HGF/MET signaling, contributes directly to MDB formation. Additionally, aHSCs promote MDB pathogenesis by regulating STAT3 via the HGF/MET axis, whereas MDB-forming hepatocytes activate HSCs through TGFβ1 secretion. Mmp14-mediated Col1a1 deposition facilitates aHSC differentiation into myofibroblasts, leading to liver fibrosis (Fig. 8). TNFα, through TNFR1 or TNFR2, activates both canonical and noncanonical NF-κB signaling,^45,46^ driving widespread gene expression changes that fuel inflammation and MDB formation. Microarray analysis of DDC-Fed mouse livers revealed upregulation of UbD and the catalytic subunits of the immunoproteasome, as we have previously shown,^30^ which is consistent with a transition from the 26S proteasome to the immunoproteasome in MDB-forming hepatocytes.

In addition to MDBs, other cytoplasmic inclusions may be present in hepatocytes including intracytoplasmic hyaline bodies (IHBs) or other aggresomes.^15^ One important feature that links MDBs to other non-MDB tissue-selective cytoplasmic inclusions is the abundant presence of keratin intermediate filament (IF) proteins.^47^ Both types of inclusions are the result of the accumulation of the stress protein p62. In MDBs, p62 is associated with keratins, whereas classical IHBs lack keratins. Therefore, a more refined understanding of the mechanism of MDB pathogenesis presented in this study should help us understand not only hepatocyte cytoplasmic inclusion-related liver diseases but also other IF-related diseases and possibly help devise directed therapeutic interventions.

Despite these findings, we recognize that this study has inherent limitations. While the DDC diet provides a reproducible model for MDB induction and mechanistic studies, the pathophysiological context of human liver disease involves more complex and heterogeneous stimuli. Therefore, studies using human liver fibrosis cohorts (autoimmune and other etiologies) and designing single-cell and spatial transcriptomic analyses of these samples to further validate HGF/MET/UbD signaling molecule colocalization within MDB-rich regions are needed to strengthen the translational significance of our findings in future studies. Additionally, although we used UbD⁻/⁻ mice to assess changes in the HGF/MET signaling pathway, the HGF/MET claim also requires in vivo causality. It is essential to design studies employing hepatocyte-targeted MET inhibition and HGF neutralization in both DDC-induced and CCl₄-induced fibrosis models to delineate their respective roles in the injury and repair phases. Furthermore, the mechanisms underlying HGF secretion, particularly in the crosstalk between hepatocytes and nonparenchymal liver cells such as HSCs, remain to be fully elucidated. The activation of HSCs provides additional paracrine signals (e.g., HGF, cytokines, and chemokines) that could further promote MDB formation.

In summary, these findings significantly extend the mechanistic understanding of MDB pathogenesis and fibrotic progression at single-cell resolution. Our findings reveal that MDBs promote HSC activation and liver fibrosis via the HGF/MET/UbD signaling pathway, providing new insights into the molecular mechanisms driving MDB formation and progression to liver fibrosis and highlighting potential therapeutic strategies for chronic liver disease.

## Materials and methods

For further details regarding the materials and methods, please refer to the supplementary information.

### Animal experiments

One-month-old male C3H mice were fed a diet containing 0.1% DDC (137030–25 G, Sigma–Aldrich) for 10 weeks to induce MDB formation, as we previously described.^20^ The mice were subsequently withdrawn from the DDC for 1 month (n = 4), during which most MDBs disappeared, and then the DDC was refed for 7 days (n = 4). The control mice were fed a protein-rich, semisynthetic standard diet. Four mice per group were used for snRNA-seq analysis across four conditions: (1) Control, (2) DDC-Fed, (3) DDC-Withdrawn, and (4) DDC-Refed. The mice were anesthetized, and blood samples, including ALT, ALP, and AST, were collected from the orbital sinus for serum analysis at Guangzhou Medical University’s Pathology Core Facility. Liver tissues were processed for (1) nuclear isolation and snRNA-seq, (2) fixation in 10% buffered zinc formalin for histology, and (3) snap-freezing at −80 °C for biochemical and immunofluorescence analyses. Histopathological features of MDBs, including steatosis and inflammation, were confirmed in all the mice (Supplementary Table 1). All procedures were approved by the Animal Ethics Committee of Guangzhou Medical University (GY2022-144 and GY2024-047).

### Mouse MDB organoid formation and drug treatment assays

MDBs were induced in the mice by being fed a DDC-containing diet for 10 weeks. Liver cells were then isolated to establish an MDB organoid model via the Mouse Liver Organoid Kit (abs9516, Absin). Liver tissues were washed three times with primary culture buffer and digested in tissue digestion solution at 37 °C for 30 minutes. Digestion was terminated with primary culture buffer, and the tissues were dissociated by repeated pipetting until a turbid suspension was obtained. The cell suspension was filtered, enriched by centrifugation at 300 rcf for 5 minutes at 4 °C, and washed twice with primary culture buffer.

The resulting cell pellet was resuspended in growth factor-free basement membrane matrix and plated as 25 μL droplets in 24-well plates. The matrix was solidified at 37 °C for 40–60 minutes before 500 μL of culture medium was added. The medium was changed every two days. Organoids exceeding 200 μm in diameter were passaged at a 1:2-to-1:4 ratio by fragmenting the organoids and reinoculating them into fresh matrix droplets.

For protein extraction, organoids were separated from Matrigel by passaging buffer, and proteins were extracted from ~1,000 organoids via RIPA lysis buffer (P0013B, Beyotime). Protein concentrations were determined via a Pierce BCA protein assay kit (23227, Invitrogen). Bright-field images were captured via a 10× fluorescence microscope (AMEX1000, Invitrogen).

To assess the role of the HGF/MET pathway in organoid growth, the organoids were treated with vehicle (DMSO) or a MET inhibitor for 12, 24, or 36 hours. Organoids were fixed in 4% paraformaldehyde (BL539A, Biosharp) overnight, paraffin-embedded, sectioned, and processed for immunofluorescence. The sections were incubated at 60 °C for 30 minutes, deparaffinized, hydrated, and subjected to antigen retrieval using citrate solution (P0081, Beyotime). Membrane permeabilization was performed with 0.25% Triton X-100 (P0096, Beyotime) for 20 minutes, followed by blocking with 5% bovine serum albumin for 1 hour. The sections were incubated overnight with primary antibodies (K8, p-AKT, p-IκB, STAT3, UbD, and p62), followed by incubation with fluorescent secondary antibodies (goat anti-rabbit Alexa Fluor 488; Invitrogen, A-11008; and goat anti-mouse Alexa Fluor 555; Invitrogen, A-31570). Nuclei were counterstained with DAPI, and imaging was performed via a laser confocal microscope.

### Statistical analysis

All the quantitative in vitro and in vivo data are presented as the means ± SDs or SEMs from at least three independent experiments, each performed in triplicate. Statistical analyses were performed via GraphPad Prism version 9.0 (GraphPad Software, California, USA). Differences were considered statistically significant at *p* < 0.05.

## Supplementary information

Supplementary materials-STTT

Western blot original

## Data Availability

All the data generated or analyzed in this study are included in this published article and supplementary files. The raw single-cell sequencing data are accessible through the Gene Expression Omnibus (GEO) database (http://www.ncbi.nlm.nih.gov/geo) under accession number GSE201569 as we recently described, and include the following samples: Control: GSM6067633; DDC-Fed: GSM6067634; DDC-Withdrawn: GSM6067635; DDC-Refed: GSM6067636. Additionally, bulk RNA-seq datasets (GSE83596, n = 24; GSE114564, n = 108) related to fatty liver disease and HCC were extracted for comparative analyses. In addition, we downloaded human NASH-related snRNA-seq data from GSE202379 and investigated the changes in the expression of MDB-related genes in hepatocytes across different stages of fatty liver disease. These data are also available upon request from the corresponding author.
