# Supplementary material for: Unraveling the HGF/MET axis in Mallory-Denk body pathogenesis associated with liver fibrosis through single-cell transcriptomics
Source: Signal Transduct Target Ther. 2026 Jun 11;11:227. doi: 10.1038/s41392-026-02722-4 (PMC13254210; doi:10.1038/s41392-026-02722-4)
Supplement: Supplementary file 1 — Supplementary materials-STTT [file 41392_2026_2722_MOESM1_ESM.docx]

**Supplementary Materials for**

**Unraveling the HGF/MET axis in Mallory-Denk body pathogenesis associated with liver fibrosis through single-cell transcriptomics**

Xiaoping Tang^1,2^, Yi Shi^1,2^, Jia Pan^1,2^, Yi Zhao^1,2^, Maoping Huang^1^, Huanhou Su^3,4^, Wanmei Zhou^1^, Xingguang Luo^5^, Xiao Luo^1^, Jinge Zhang^1^, Jiuying Zhao^1^, Jiangtao Jia^1^, Jinru Li^1^, Bei Zhong^1^, Lan Tang^1^, Zhuo Chen^1^, Yongchang Ouyang^1^, Zhichun Dang^1^, Samuel W. French^6^, Yihui Huang^1,2,^*, Xinghua Pan^3,7,^*, Hui Liu^1,2,^*

Correspondence to: [liuhui806@gzhmu.edu.cn](mailto:liuhui806@gzhmu.edu.cn)

[panxinghua@must.edu.mo](mailto:panxinghua@must.edu.mo)

hyh930@sina.com

**This PDF file includes:**

Materials and Methods

Figures. S1 to S21

Tables. S1 to S4

**Other Supplementary Materials for this manuscript include the following:**

References

Clinical information

**MATERIALS AND METHODS**

Ethics statement and clinical specimen acquisition

Human formalin-fixed paraffin-embedded (FFPE) liver biopsies from HCC patients (n = 20) were obtained from the archives of the Affiliated Qingyuan Hospital, Guangzhou Medical University, with Institutional Review Board approval (IRB-2022-094). Serum parameters were measured via standard procedures at the hospital’s Clinical Laboratory (Supplementary Table 2). Biopsies with significant MDB formation (n = 5) were evaluated by experienced pathologists and selected for further analysis, following established protocols. Normal liver tissues were used as controls. The study adhered to the principles of the Declaration of Helsinki, and all procedures involving human samples were approved and registered at Medicalresearch.org.cn by the tissue bank of the Affiliated Qingyuan Hospital, Guangzhou Medical University (ChiCTR2400084248).

Induction of liver fibrosis

To induce fibrosis alongside MDB formation, one-month-old male C3H mice were fed 0.1% DDC for 10 weeks. Beginning at week 5, mice received 5 mL/kg CCl₄ (HY-Y0298, MCE) in corn oil (10%) by intraperitoneal injection twice weekly (n = 3) as described previously.[^1^](#_ENREF_1) Three control mice were fed the standard diet (n = 3). Liver tissues were processed for (1) fixation in 10% buffered zinc formalin for histology and (2) snap-freezing in liquid nitrogen for biochemical assays. All procedures were approved by the Animal Ethics Committee of Guangzhou Medical University (GY-2025-091).

Generation of UbD knockout (KO) mice

UbD KO mice (UbD⁻/⁻) were generated using CRISPR/Cas9 technology as previously described.[^2^](#_ENREF_2) Guide RNAs targeting exon 2 of the UbD locus (gRNA1: TGTTCTCCTGGTTGGCCCTC; gRNA2: TCTAACATAGTTCATTCGCA) were microinjected with Cas9 protein into fertilized C3H eggs. Genotypes of 10-day-old pups were confirmed by PCR with primers: Forward 5′-AGCACAGCATCACCACAACA-3′, Reverse 5′-TCAGGTGGAGTCTCTCTCGG-3′. F0 mice carrying deletion alleles were backcrossed with C3H mice to generate F1 heterozygotes, which were interbred to produce homozygous, heterozygous, and wild-type mice. PCR products: wild-type allele 1554-bp and 416-bp; heterozygous allele 1554-bp, 820-bp, 416-bp; homozygous KO allele 820-bp. F1 homozygous UbD KO mice were selected for experiments. Both UbD⁻/⁻ and wild-type mice were fed a normal diet containing 0.1% DDC for 8 and 10 weeks, and liver tissues and blood samples (n = 3 per group) were collected. All procedures were approved by the Guangzhou Medical University Institutional Animal Care and Use Committee (GY2022-101).

Generation of UbD⁻/⁻ fibrotic mice and treatment

To establish UbD⁻/⁻ and wild-type (WT) fibrotic mouse models, both groups were fed a normal diet containing 0.1% DDC for 8 weeks. Starting at week 5, mice received intraperitoneal injections of 5 mL/kg carbon tetrachloride (CCl₄) dissolved in 10% corn oil twice weekly (n = 3 per group). Liver tissues were collected for subsequent analyses. All procedures were approved by the Institutional Animal Care and Use Committee of Guangzhou Medical University.

Histological analysis

Mouse livers were rapidly frozen in isopentane cooled with liquid nitrogen. Tissue sections (5 μm) were cut at −20 °C, transferred to a micro slide box on dry ice, and stored at −80 °C. Slides were air-dried, formalin-fixed, and paraffin-embedded. Images were captured using a Leica DM6B fluorescence microscope (Germany) with Leica Application Suite software. Hepatocyte ballooning, steatosis, MDB formation, and inflammation were assessed by a pathological expert using a semiquantitative scoring system (0 = none, 1 = rare, 2 = frequent, 3 = abundant).

H&E, Oil Red O, and Masson Staining

Hematoxylin and eosin (H&E) staining was performed on mouse and human HCC tissue sections following standard protocols and analyzed at the pathological core of Guangzhou Medical University. MDBs were quantified by counting ten random fields per liver section at 20× magnification, with evaluations performed blinded to treatment group (mean ± SEM; n = 4 for DDC-fed group). Oil Red O and Masson staining were performed using standard protocols. Stained sections were sealed with a water-soluble sealing agent and neutral gum, imaged using a PRECICE 500 slide scanner system (UNIC), and processed with iViewer software.

Serum biochemistry assay

Serum samples were collected from all experimental groups. ALT, AST, ALP, and total bilirubin were measured using standard procedures at Wuhan Servicebio Biotechnology Co., Ltd (China).

Immunostaining analysis

FFPE liver tissue slides from human HCC biopsies and DDC-treated mouse livers were double-stained for various proteins: 1. Human HCC biopsies and DDC-treated mouse tissues: Slc7a11 (26864-1-AP, Proteintech), p-MET (AF3129, Affinity), p-AKT (AB192623, Abcam), p-IκBα (AB133462, Abcam), UbD (F40095, NSJ Bioreagents), and p62 (sc-48402, Santa Cruz). 2. Mouse liver tissues: K8 (AB53280, Abcam), Slc7a11, p-AKT, UbD, p-IκBα, and p62. 3. HCC and mouse tissues: HGF (DF6326, Affinity) and F4/80 (NB600-404SS, NOVUS). Slides were counterstained with DAPI (C1002, Beyotime) for nuclei and imaged using a ZEISS LSM880 laser confocal microscope equipped with photomultiplier tube (PMT) detectors. Images (1024 × 1024 pixels) were processed with ZEN software (ZEISS). Co-localization analysis was conducted using the JAcoP plugin in ImageJ, with Pearson’s correlation coefficient used to quantify spatial colocalization between two fluorescent channels (values range from −1 to 1; >0.5 indicates significant co-localization).

Real-time quantitative polymerase chain reaction (RT‒qPCR)

Total RNA from cells or tissues was extracted and reverse transcribed into cDNA using PrimeScript RT Master Mix (RR036A, Takara). RT‒qPCR was performed with the TB Green® Premix Ex Taq™ II kit (RR820A, Takara) on a LightCycler® 480 II Real-Time PCR System (Roche) as previously described.[^3^](#_ENREF_3) Target mRNA levels were normalized to endogenous controls and analyzed using the ΔΔCT method. Reactions were performed in triplicate. Primer sequences are listed in Supplementary Table 3.

Nuclei isolation

Hepatocyte nuclei were isolated following a modified protocol.[^4^](#_ENREF_4) Liver tissues from six regions of Control, DDC-Fed, DDC-Withdrawn, and DDC-Refed mice were pooled and minced. Tissue was resuspended in 5 mL lysis buffer (250 mM sucrose, 5 mM CaCl₂, 3 mM MgAc₂, 10 mM Tris-HCl, 1 mM DTT, 0.1 mM EDTA, 1× protease inhibitor, 1 U/μL RiboLock RNase inhibitor) and homogenized with a Dounce tissue grinder (15 strokes). Homogenates were centrifuged at 1000 × g for 5 min at 4°C to pellet nuclei.

The pellet was mixed with an equal volume of 50% iodixanol solution (160 mM sucrose, 3 mM MgCl₂, 10 mM NaCl₂, 10 mM Tris-HCl, 1 mM DTT, 0.1 mM PMSF, 1 U/μL RiboLock RNase inhibitor) and layered over 33% iodixanol. After centrifugation at 10,000 × g for 30 min at 4°C, nuclei were collected from the 30–35% interface, washed in nuclei resuspension buffer (0.04% BSA, 0.2 U/μL RiboLock RNase inhibitor, 0.1 mM PMSF, 500 mM mannitol in PBS), and pelleted at 1000 × g for 5 min at 4°C. Nuclei were filtered through a 40-μm strainer, counted using DAPI staining and a hemocytometer, and adjusted to ~1200 nuclei/μL for immediate processing with the 10× Genomics® Single Cell 3’ system. Matched Control and DDC-Fed samples were loaded on the same chip to reduce batch effects.

Single-nucleus RNA-sequencing (snRNA-seq)

snRNA-seq libraries were constructed from four independent groups of adult mice at four time points: DDC-Fed, DDC-Withdrawn, DDC-Refed, and healthy controls (n=3 per group) using Chromium Single Cell 3′ Reagent Kits v3.1 (10× Genomics) according to the manufacturer’s instructions. Individual cells (~500–10,000 per sample) were encapsulated in Gel Bead-In-Emulsions (GEMs) containing unique barcodes. After cDNA generation, silane magnetic beads removed residual reagents, and full-length, barcoded cDNA was amplified by PCR. Libraries were compatible with Illumina platforms and sequenced on a NovaSeq 6000. Read 1 contained the 16 bp 10× barcode and 10 bp UMI; Read 2 sequenced the cDNA fragment; the i7 index read contained the sample index.

Quality control and gene expression quantification

Raw sequencing data were processed with the Cell Ranger software pipeline (v2.0.0, 10× Genomics) for demultiplexing, barcode assignment, and UMI counting. Reads were aligned to the mouse reference genome (Ensembl GRCm38.p6, release 98) using the STAR aligner (<https://github.com/alexdobin/STAR>).Cell Ranger classifies reads into exonic, intronic, or intergenic categories based on their alignment. Reads are designated as exonic if at least 50% of the sequence overlaps an exon, intronic if non-exonic and overlapping an intron, and intergenic otherwise.

Exonic reads are further aligned to annotated transcripts to determine compatibility. Reads aligning to the exons of a single annotated transcript on the same strand are considered uniquely mapped to the transcriptome. Only confidently mapped reads are included in UMI counting. The number of recovered cells is estimated as the 99^th^ percentile of the top N barcodes by total UMI counts, with barcodes exceeding m/10m in total UMI counts identified as cells.

Cell/nuclei clustering

Downstream analysis was conducted using the Seurat package. Gene expression matrices were normalized using the “LogNormalize” method, scaling each cell’s gene expression by total counts, multiplying by a scale factor of 10,000, and applying a log transformation. Dimensionality reduction was performed via Principal Component Analysis (PCA).

Significant principal components (PCs) were selected determined using a jackStraw-based resampling test. It constructs a null distribution by permuting 1% of the data and identifying PCs enriched with low p-value genes.[^5^](#_ENREF_5) A K-nearest neighbors (KNN) graph was generated based on Euclidean distances in PCA space, with edge weights refined by shared neighborhood overlap. Clusters were visualized using t-SNE or UMAP based on principal components. For major liver cell types, we used at least two canonical markers to define each cluster, including Cycling cells (Top2a, Mki67), Stellate cells (Dcn, Lrat, GUCY1B1), Kupffer cells (Cd68, Clec4f), Cholangiocyte (Epcam, Krt19), Endothelial cells (PTPRB, Cdh5, Pecam1), Hepatocytes (Albumin, Pck1, F5, Hnf4a), DC (Cd83, Xcr1), B cells (Blnk, Ighm), and T cells (Cd3g, Cd3e).

To explore similarity relationships among clusters, the function "BuildClusterTree" was used to construct a hierarchical tree of expression profiles. For clustering, a resolution of 0.5 was used. Harmony correction was applied to remove batch effects, and PCs 1–50 were used for dimensionality reduction. The top 2000 highly variable genes were selected for downstream analysis, and all other parameters were kept at their default values.

Differential expression, cell trajectory and pathway analysis

Differentially expressed genes (DEGs) for each cluster were identified using a likelihood-ratio test, comparing each cluster to all other cells. Peak-related genes were mapped to Gene Ontology (GO) terms (<http://www.geneontology.org/>) and significantly enriched terms were determined by a hypergeometric test with FDR ≤ 0.05. Similarly, enriched metabolic and signal transduction pathways in DEGs were identified using the Kyoto Encyclopedia of Genes and Genomes (KEGG) database. Top marker genes for each cluster were visualized using heatmaps and bubble plots, and cell-type identities were manually assigned based on a curated panel of known cell-type transcripts.

Cell trajectories were inferred using Monocle,[^6^](#_ENREF_6) which identifies DEGs between cell groups and evaluates statistical significance. Key genes involved in development and differentiation were identified with FDR < 1e-5. Genes with similar expression dynamics were grouped, assuming shared biological functions. Pathway enrichment for hepatocyte clusters was further assessed using Gene Set Variation Analysis (GSVA, Version 2.2.1).

Prediction of cellular crosstalk

CellPhoneDB (Version 5.0.1)[^7^](#_ENREF_7) was used to infer ligand-receptor interactions between cell types. Pairwise comparisons were performed across all clusters, with cluster labels randomly permuted 1,000 times to assess statistical significance. For each ligand-receptor pair, the mean ligand expression in one cluster and receptor expression in the interacting cluster were used to generate a null distribution. *P*-values < 0.05 were considered significant. A cell interaction network was constructed based on the number of significantly enriched ligand-receptor pairs to visualize intercellular regulatory relationships.

Evaluation of cellular subpopulation differentiation potential

CytoTRACE^[8](#_ENREF_8)^ is a computational method that predicts cell potency and developmental potential from single-cell RNA sequencing data. Using an interpretable deep learning framework, CytoTRACE assesses the differentiation state of individual cells, validated across large-scale datasets. Each cell received a CytoTRACE score ranging from 0 to 1, with higher scores indicating greater stemness (less differentiation) and lower scores representing more differentiated states.

Inference of Copy Number Variations (inferCNVs)

InferCNV^[9](#_ENREF_9)^ was employed to infer large-scale CNVs from snRNA-seq data by comparing expression profiles of individual cells to a reference population of non-mutated cells. In this analysis, endothelial cells were used as the reference, and CNVs across all hepatocyte clusters were calculated. A sliding window approach across genomic regions detected significant deviations in gene expression indicative of amplifications or deletions. CNVs were visualized across single-cell transcriptomes to identify heterogeneous subpopulations with genomic alterations.

Gene expression, cellular composition analysis and survival analysis

To investigate the potential link between cellular features and clinical outcomes in patients, this study utilized snRNA-seq data to derive gene expression and survival information from the TCGA-LIHC cohort (<https://portal.gdc.cancer.gov/>). The "Bisque" R package [^10^](#_ENREF_10) facilitates the deconvolution of cellular composition from bulk RNA sequencing data by using scRNA-seq data as a reference. Initially, the gene expression matrices from both bulk RNA sequencing and snRNA-seq datasets are processed through the "ExpressionSet" function. Following this, the "ReferenceBasedDecomposition" function is applied, leveraging snRNA-seq data as a reference to deconvolute the bulk RNA-seq data via a linear regression model. This method estimates the contribution of different cell types to the bulk sample, providing cellular composition proportions for each cell type within the bulk sample. To visualize the expression of MDB-related genes, a bar plot was created using the "ggplot2" package in R. For survival analysis, the "survival" package was applied. A survival object is created from a dataset containing survival time, status, and covariates using the *surv* function. Kaplan-Meier curves were fitted using *survfit* and visualized with *ggsurvplot*. The *arrange_ggsurvplots* function formatted output for presentation. In addition, we also downloaded human NASH-related snRNA-seq data from GSE202379 and displayed the expression changes of MDB-related genes in hepatocytes across different stages of fatty liver disease.

In vitro induction of MDBs

Hepa1-6 cells (1 × 10^6^, iCell-m019, iCell Bioscience) were induced to form MDBs, as previously described,[^3^](#_ENREF_3)^,^ [^11^](#_ENREF_11) by continuous treatment with TNFα (40 ng/ml, 315-01A, PeproTECH) and IFNγ (400 ng/ml, C746, Novoprotein) for 10 days, with media refreshed every three days. Cells were subsequently treated with HGF (20 ng/ml), the MET inhibitor PF-02341066 (HY-50878, MedChemExpress), or the PI3K inhibitor LY294002 (HY-10108A, MedChemExpress) for downstream assays.

Immunofluorescence was performed to co-stain K8, p-MET, p-AKT, and UbD with p62; nuclei were counterstained with DAPI. Fluorescence microscopy was used for imaging. Western blot analysis quantified total MET (25869-1-AP, Proteintech), AKT (AB179463, Abcam) and IκBα (AB32518, Abcam), as well as phosphorylated MET, AKT, and IκBα, as well as total UbD, with β-actin (AC026, ABclonal) serving as a loading control.

MDB-forming coculture assays

Hepa1-6 cells were treated with TNFα (40 ng/mL) and IFNγ (400 ng/mL) for 10 days to induce MDB formation. Subsequently, 1 × 10⁶ liver cells were seeded in the apical chamber of Transwell-24 inserts (6.5 mm diameter, 3 μm pores; Nest, CN) and allowed to attach for 12 hours. The medium was replaced with 100 μL DMEM (C11995500BT, Gibco) containing 5% FBS. Inserts were then transferred to macrophage-seeded wells (n=3 per phenotype) in the lower compartment containing high-glucose DMEM with 10% FBS. After 12 hours of coculture, cells and supernatants were harvested. Macrophages were lysed with 350 μL LB Buffer (Yeasen, CN) for RT-qPCR and HGF ELISA Kit (E-EL-H0084C, Elabscience).

JS-1 cells (2 × 10^5^, HTX2388, OTWO Biotech) were cultured in DMEM supplemented with 10% FBS and 1% penicillin-streptomycin. To induce HSC activation, JS-1 cells were starved for 24 hours and treated with TGFβ (10 ng/mL, CK33, Novoprotein) for 12, 24, or 48 hours. For HSC/hepatocyte coculture, organoids and JS-1 cells were cultured at a 5:1 ratio, reflecting the hepatocyte: HSC ratio in DDC-fed mice. Approximately 100 organoids (>200 μm in diameter, ~10⁴ cells each) were mixed with 2 × 10⁵ JS-1 cells in a 24-well plate for direct coculture. For indirect coculture, JS-1 cells were cultured in the upper lumen, and organoids in the lower lumen, maintaining the same cell ratio. Cocultures were maintained for 96 hours in a 1:1 medium mixture. Supernatants were collected, centrifuged at 1,500 rpm for 10 minutes at 4°C, TGFβ1 (88-8350-88, Invitrogen), and Mmp14 (UpingBio, SYP-M0312 levels were measured following standard ELISA protocol.

Hepa1-6 cells pretreated with TNFα and IFNγ to form MDB and then were cocultured with JS-1 cells at a 1:1 ratio. Approximately 5 × 10⁵ MDB-forming hepatocytes were mixed with 5 × 10⁵ JS-1 cells in a 24-well plate for indirect coculture. JS-1 cells were cultured in the lower lumen, and hepa1-6 cells in the upper lumen, maintaining the same cell ratio. Co-cultures were maintained for 24 hours in a 1:1 medium mixture. The Met inhibitor was dissolved into a 10 mM stock solution according to the manufacturer’s instructions, and the stock solution was further diluted with medium to a working concentration of 10 μM (designated as Met-1) for subsequent use. The blank group received 1 mL of medium per well, while the Met-treated group was supplemented with 1 mL of Met-1 per well. All groups were incubated in a cell culture incubator, and the supernatants were collected after 24 hours of incubation. Supernatants were collected, centrifuged at 1,500 rpm for 10 minutes at 4°C, and TGFβ1, Col1a1 (UpingBio, SYP-M0849), and α-SMA (UpingBio, SYP-M2212) levels were measured using ELISA protocol.

Enzyme-linked immunosorbent assay (ELISA)

ELISA kits were used to quantify TNFα (BMS607-3, Thermo Fisher), IFNγ (BMS6027, Thermo Fisher), IL-6, and HGF levels in serum samples (dilution 1:200). TNFα and HGF concentrations in culture media were also measured according to the manufacturer’s instructions.

Protein extraction and immunoblotting

Total protein was extracted from whole-cell lysates, mouse tissues, and five HCC tissue samples stored in liquid nitrogen using RIPA lysis buffer (P0013B, Beyotime) following the manufacturer’s protocol. Mouse membrane proteins were isolated using the Membrane and Cytosol Protein Extraction Kit (P0033, Beyotime). Protein concentrations were determined using the Pierce BCA Protein Assay Kit (23227, Thermo Fisher). Proteins were resolved by SDS-PAGE and transferred onto 0.45 μm PVDF membranes (IPVH00010, Immobilon). Western blotting was performed as previously described.[^3^](#_ENREF_3) Target proteins analyzed included total and phosphorylated MET, STAT3 (4904T, CST), phosphorylated STAT3 (9145T, CST), Mmp14 (A2549, ABclonal), AKT, IκBα, p50/105 (14220-1-AP, Proteintech), α-SMA (14395-1-AP, Proteintech), Vimentin (T55134, Abmart), phosphorylated p50/105 (TA3219, Abmart), and total UbD. To quantify the Western blot bands, we utilized ImageJ software following the standard protocol and relative protein expression was performed using (phosphorylated target proteins)/(total target proteins) or (target proteins)/(internal reference protein) respectively.

**Supplementary figures**

**
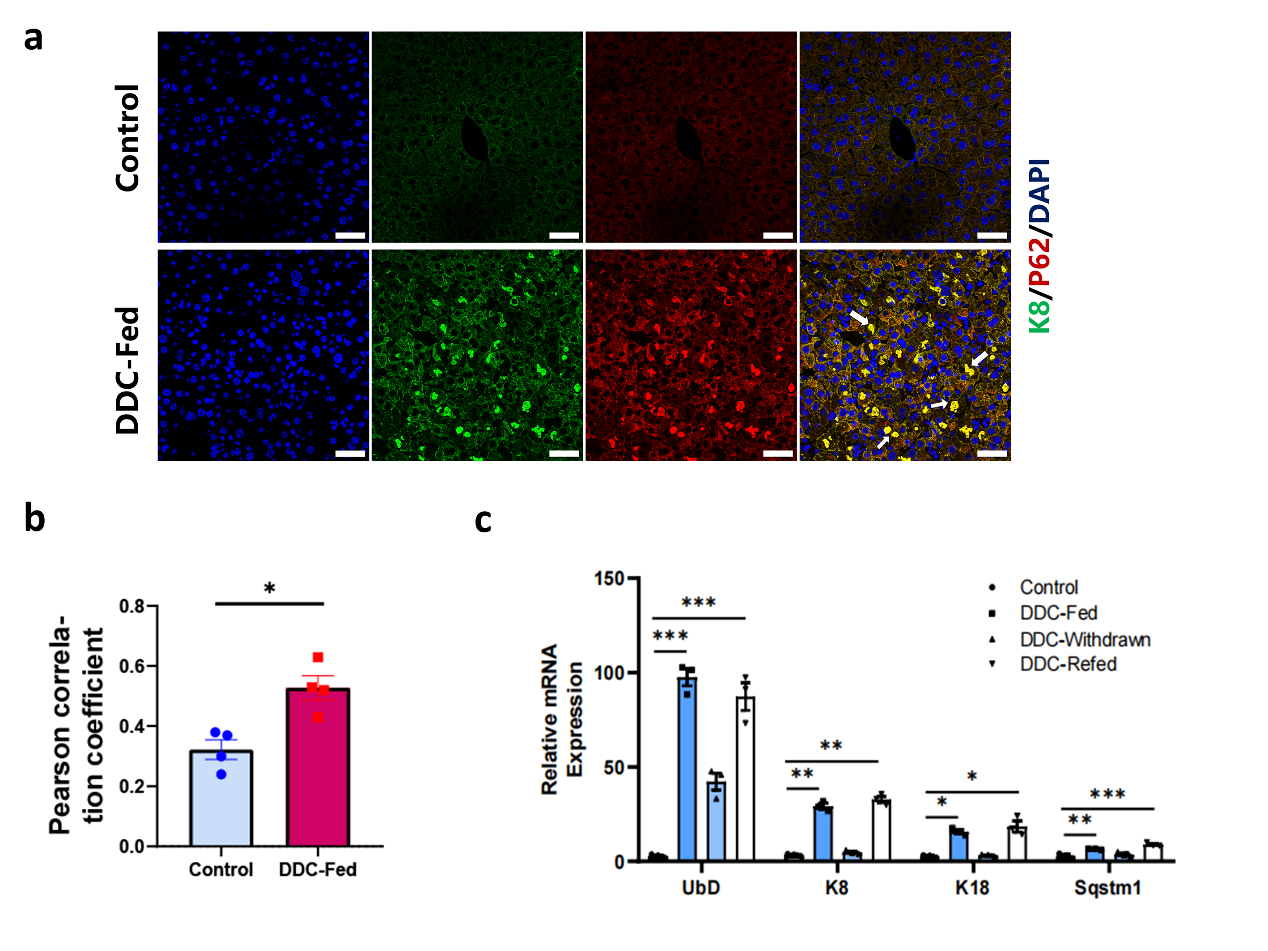
**

**Figure. S1.** MDB-related genes analysis in MDB formation upon DDC treatment

**(a)** Double immunofluorescent staining of liver sections from DDC-Fed and Control groups with antibodies against K8 (green), P62 (red), and DAPI (blue). MDBs (arrow) were stained yellow because they were both P62-positive and K8-positive. Scale bar: 50 μm. (**b**) Morphometric quantiﬁcation of immunoﬂuorescent staining demonstrates co-localization analysis of MDBs in mouse liver tissues using Pearson correlation coefficient display (n=4 mice/group). (**c**) Quantification of mRNA expression of UbD, K8, K18 and Sqstm1 in the livers of Control, DDC-Fed, DDC-Withdrawn and DDC-Refed samples. Data are shown as mean ± SEM (n=4). *p*<0.05*; *p*<0.01**; *p*<0.001***.

**
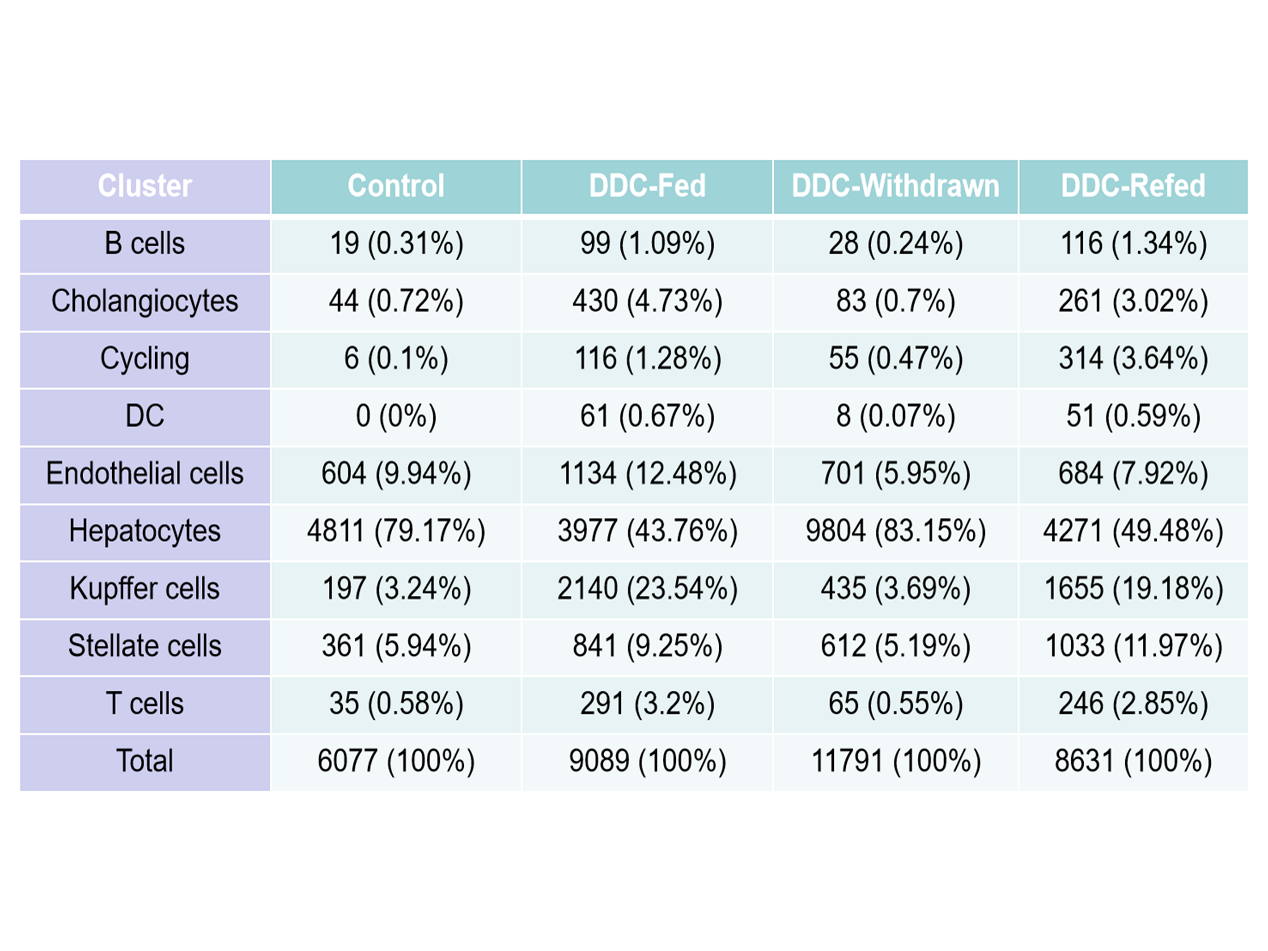
**

**Figure. S2.** Proportional changes of nine heterogeneous clusters in Control, DDC-Fed, DDC-Withdrawn, and DDC-Refed samples.

**
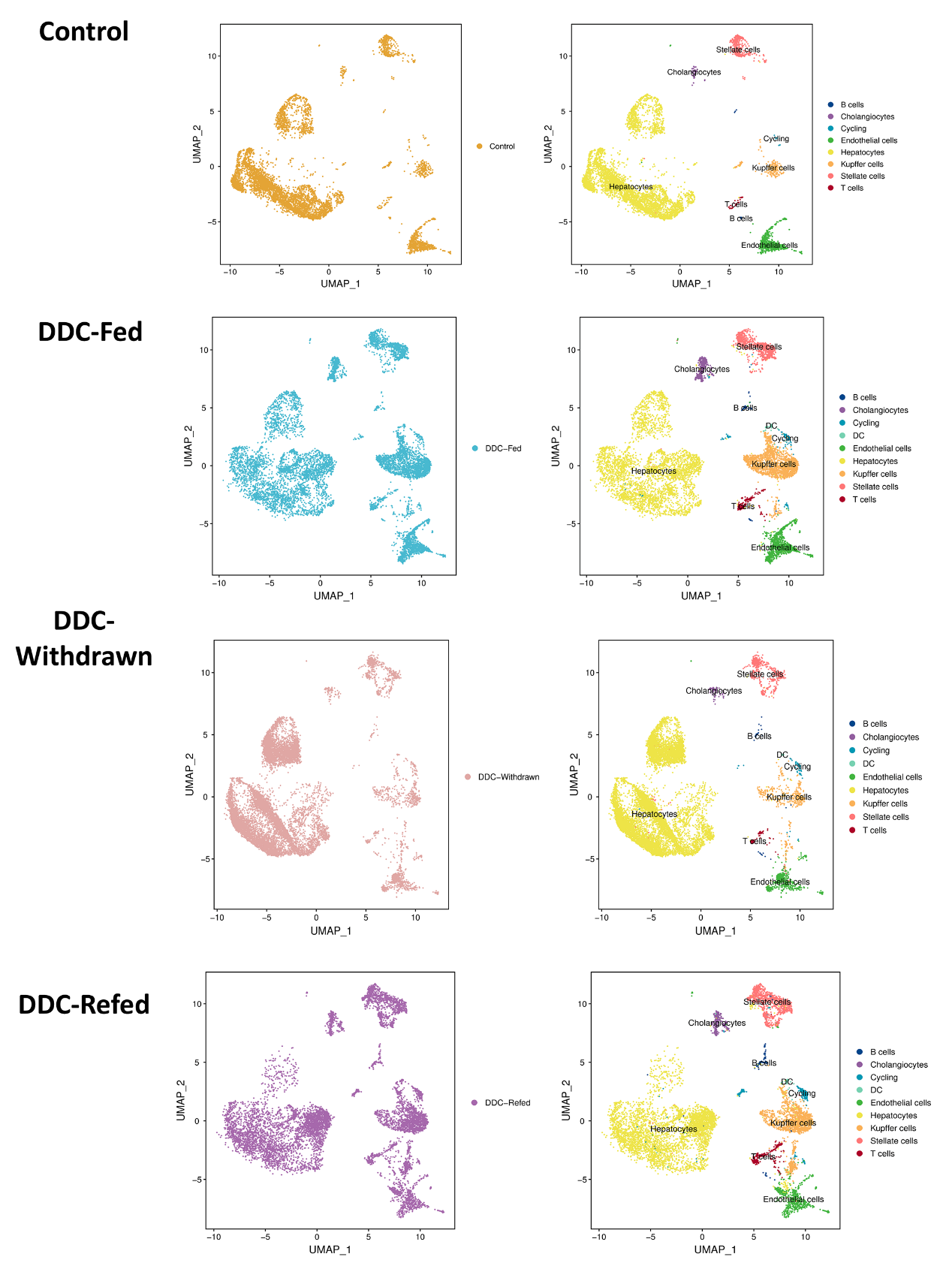
**

**Figure. S3.** UMAP projection of the single-cell atlas from Control, DDC-Fed, DDC-Withdrawn, and DDC-Refed groups, showing nine major cell types categorized by sample origin and cell type.

**
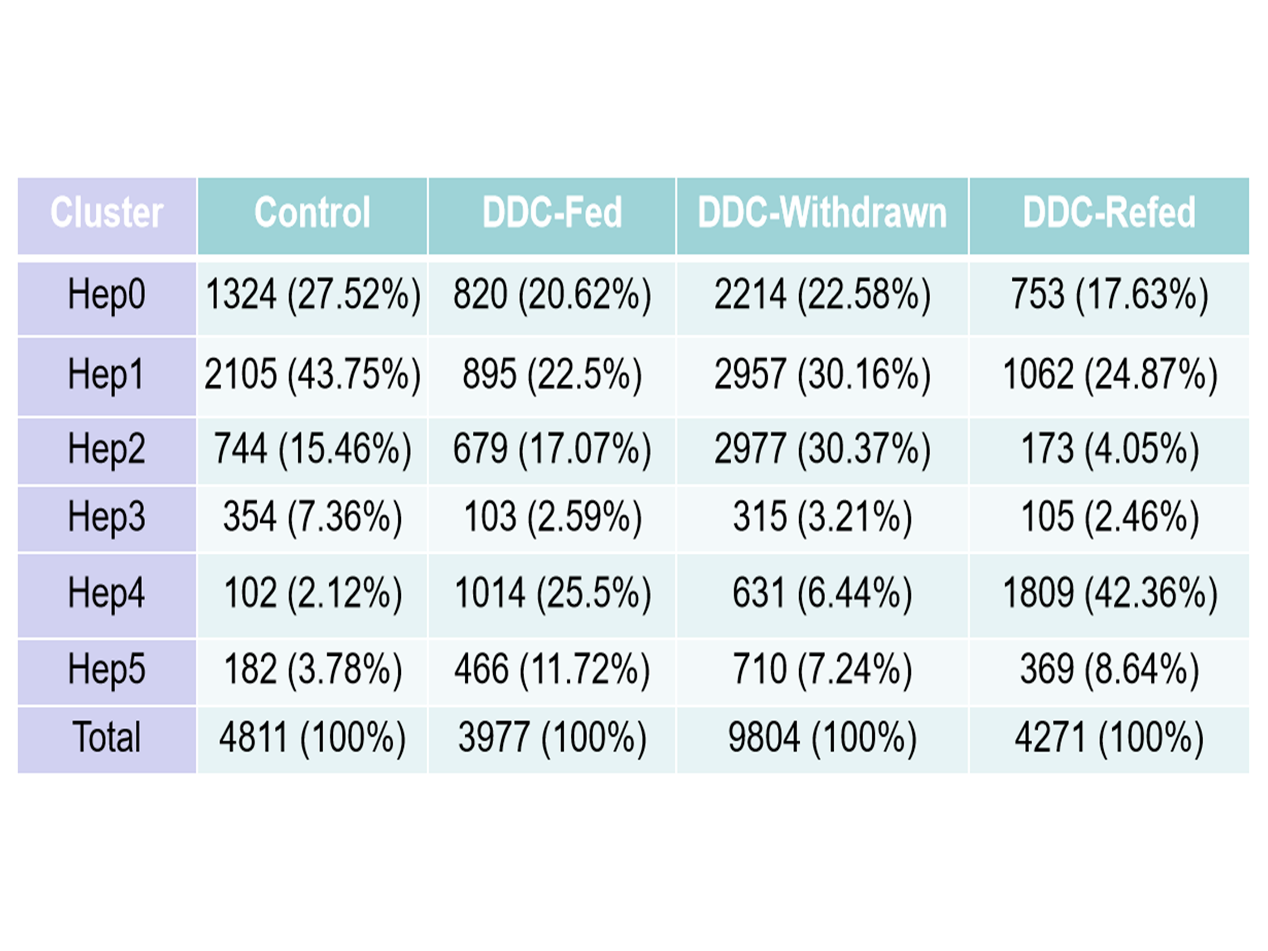
**

**Figure. S4.** Distribution of hepatic subtypes across four experimental groups.

**
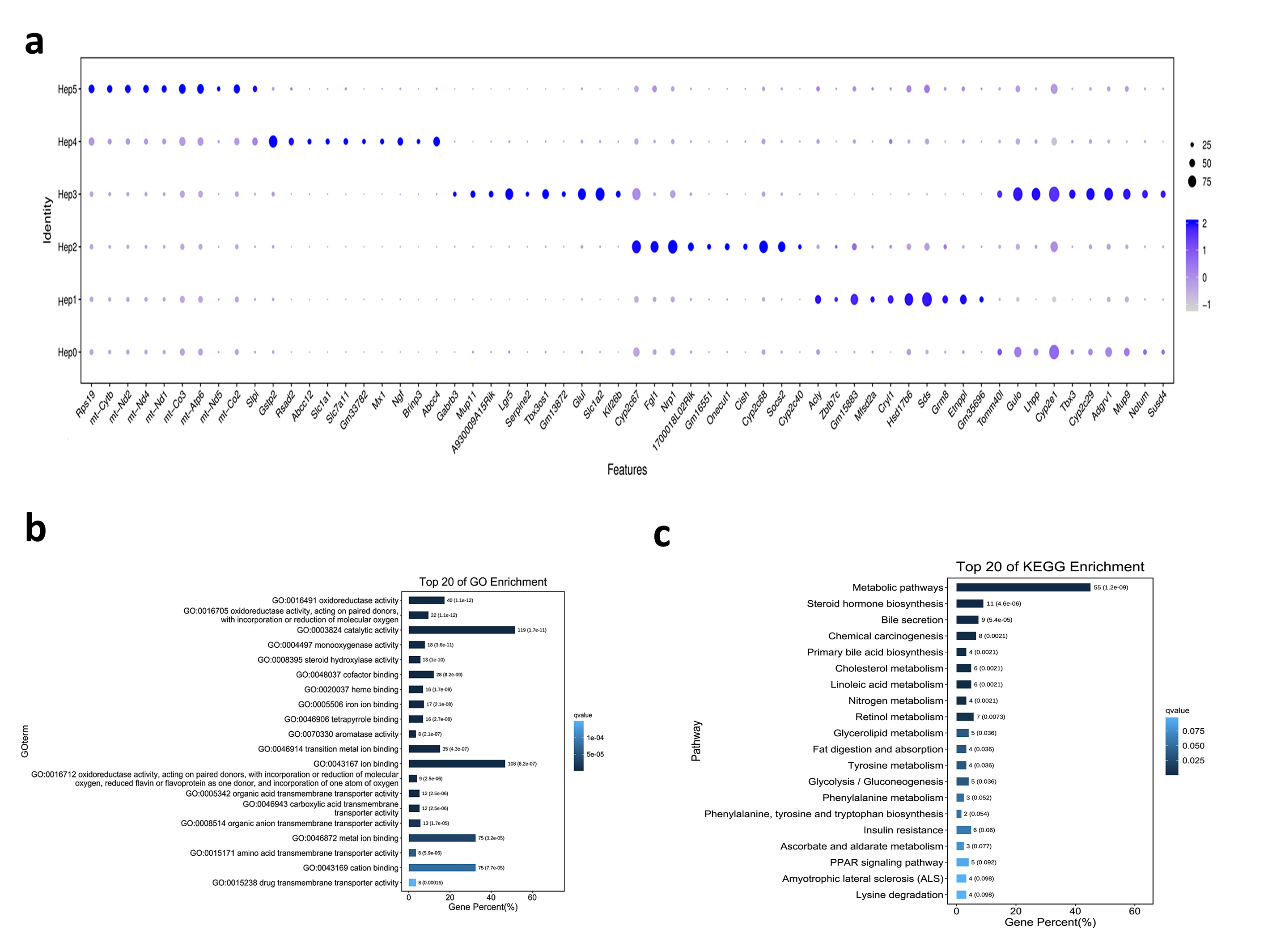
**

**Figure. S5.** Expression and pathway analysis of Hep0. Bubble chart showing the top 10 DEGs across six hepatic subtypes (**a**) and pathway analyses of Hep0 by Gene Ontology (GO) (**b**) and KEGG (**c**) analysis.

**
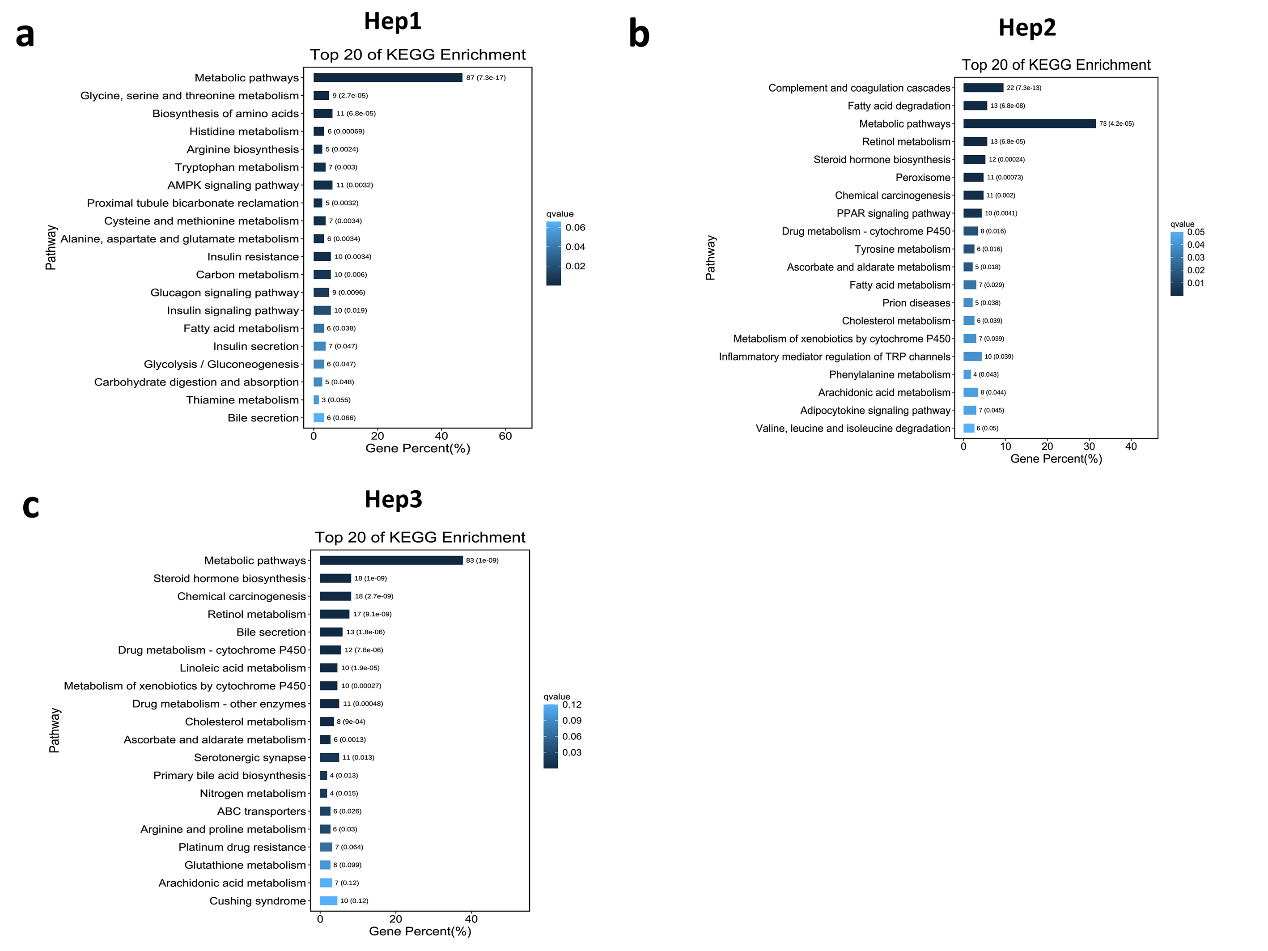
**

**Figure. S6.** KEGG pathway enrichment analyses of Hep1 (**a**), Hep2 (**b**), and Hep3 (**c**) subtypes.

**
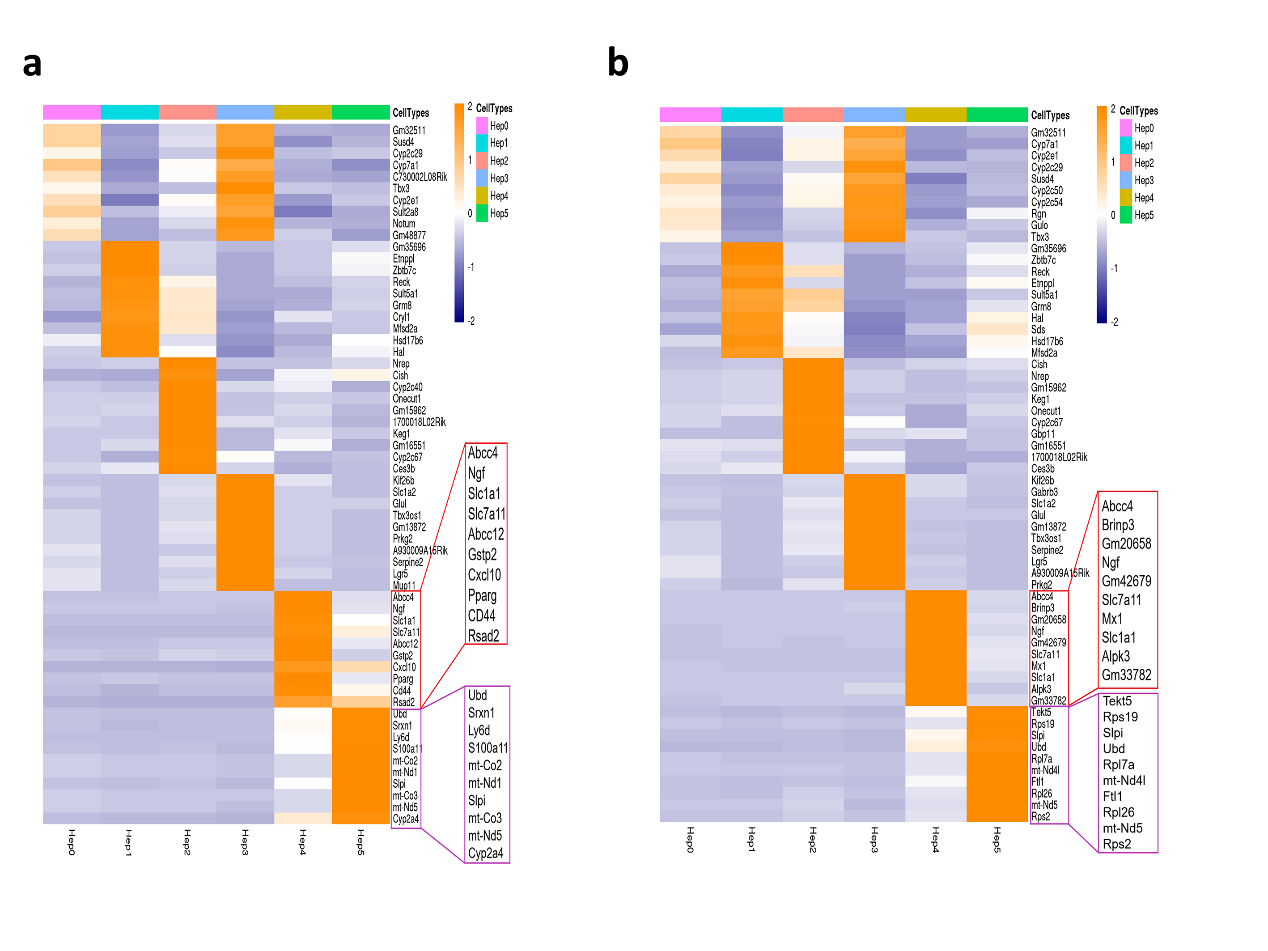
**

**Figure. S7.** mtDNA depletion in livers of Control and DDC-Fed mice (**a**) and Control versus DDC-Refed mouse livers (**b**).

**
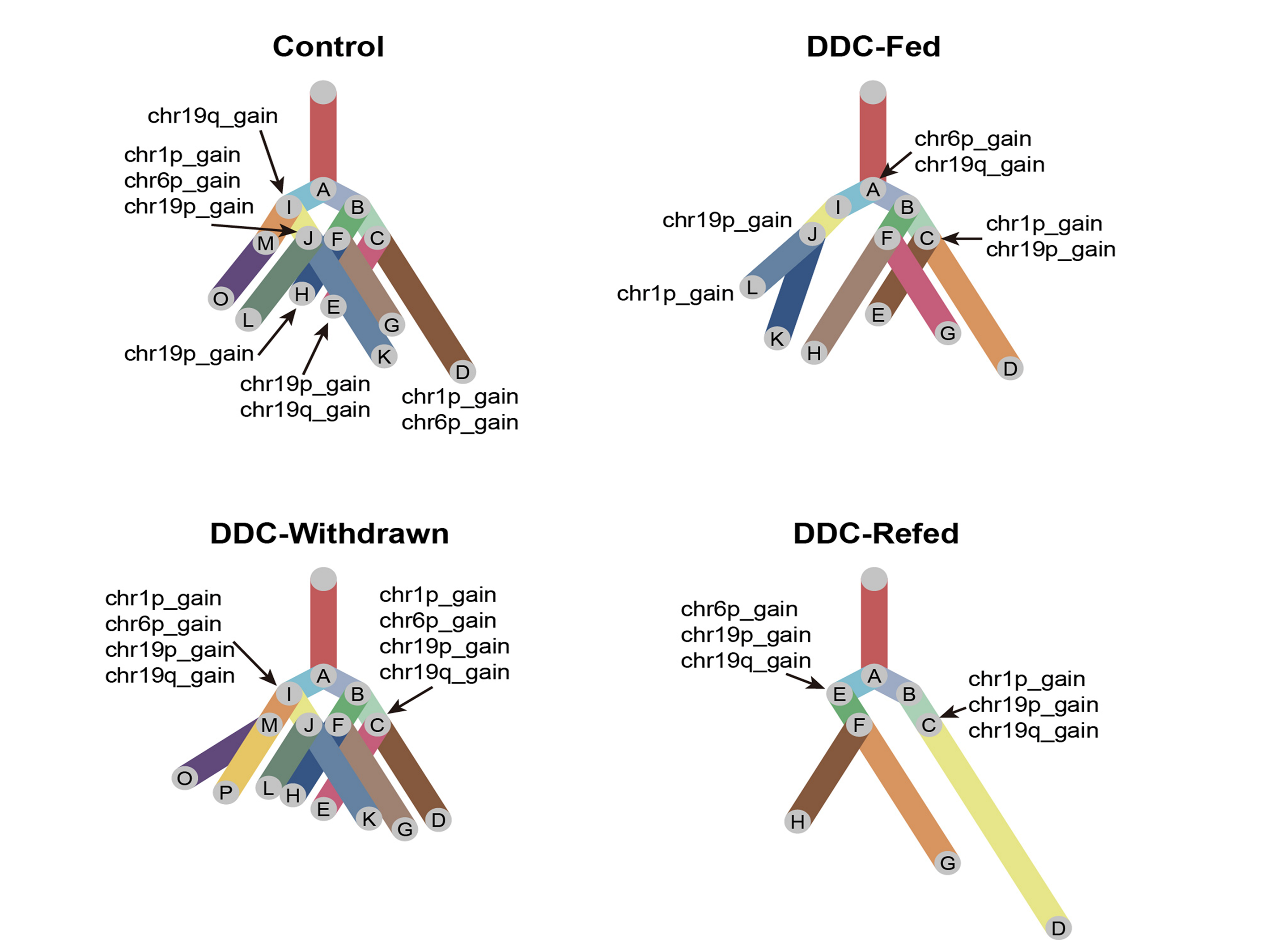
**

**Figure. S8.** Clonal Evolution Analysis based on UPhyloplot2 and InferCNV results. The figure illustrates the clonal evolution analysis, highlighting CNV events of chr1p_gain, chr6p_gain, chr19p_gain, and chr19q_gain.

**
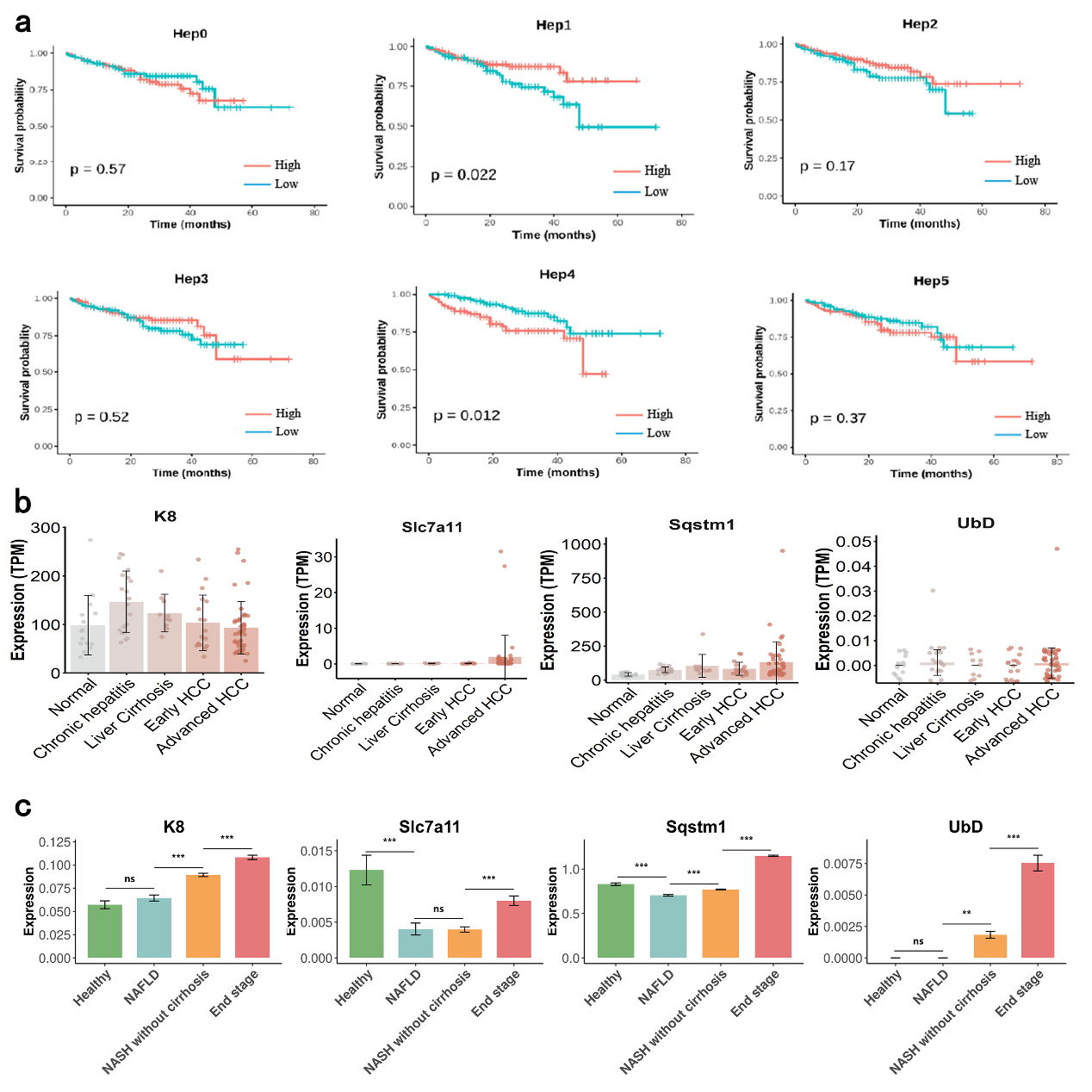
**

**Figure. S9.** Survival curves of hepatocyte subsets and expression analysis of MDB-related molecules in public database

**(a)** Kaplan–Meier survival curves of hepatocyte subsets in the LIRI database. Red line: high cell proportion; turquoise line: low cell proportion. (**b)** Expression of K8, Slc7a11, Sqstm1, and UbD across different liver disease stages in a public database. **(c)** Analysis of expression levels of K8, Slc7a11, Sqstm1, and UbD across different stages of liver disease in human public snRNA-seq dataset.

**
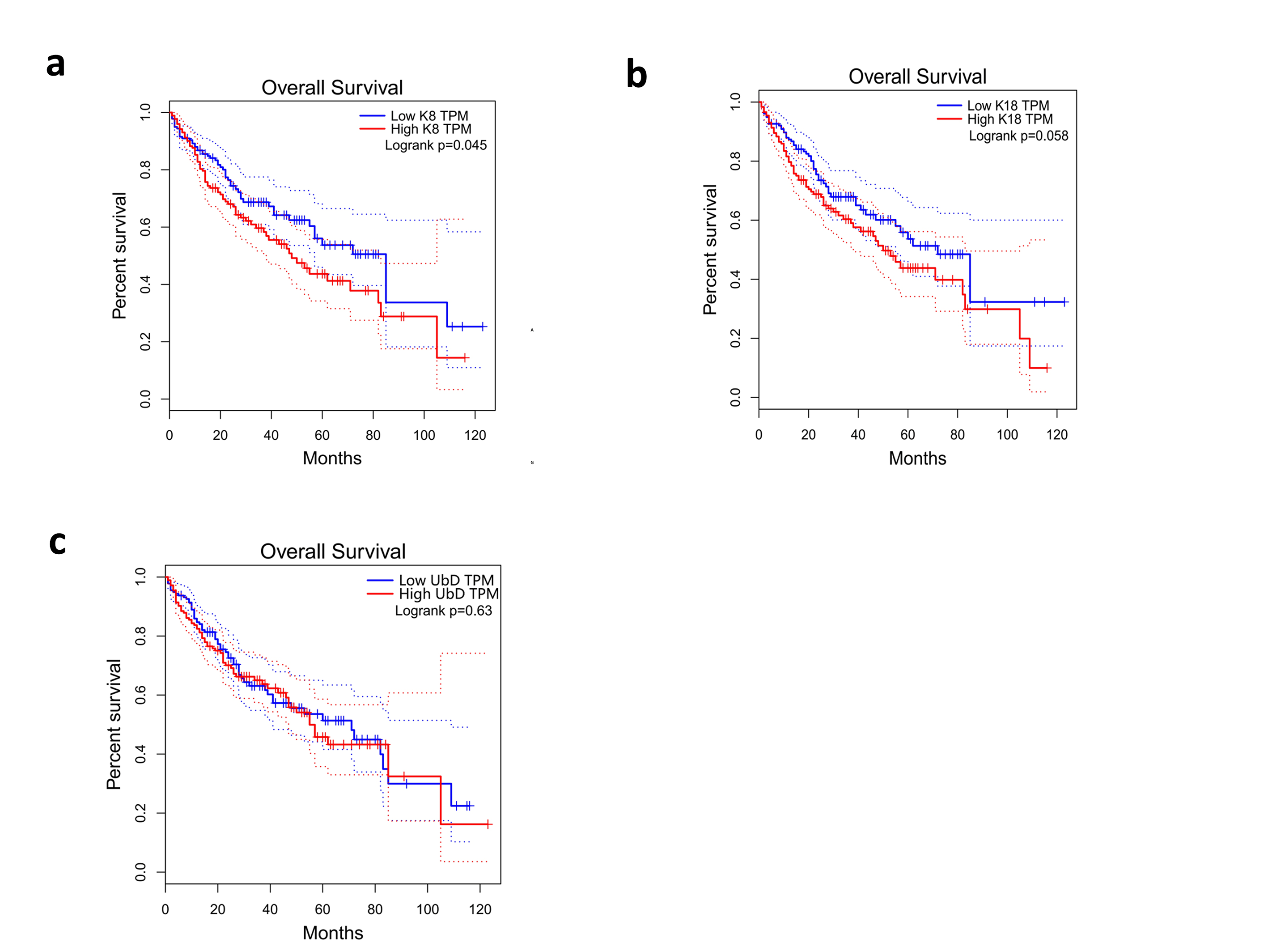
**

**Figure. S10.** Kaplan–Meier survival analysis of K8 **(a)**, K18 **(b)** and UbD **(c)** expression in TCGA-LIHC.

**
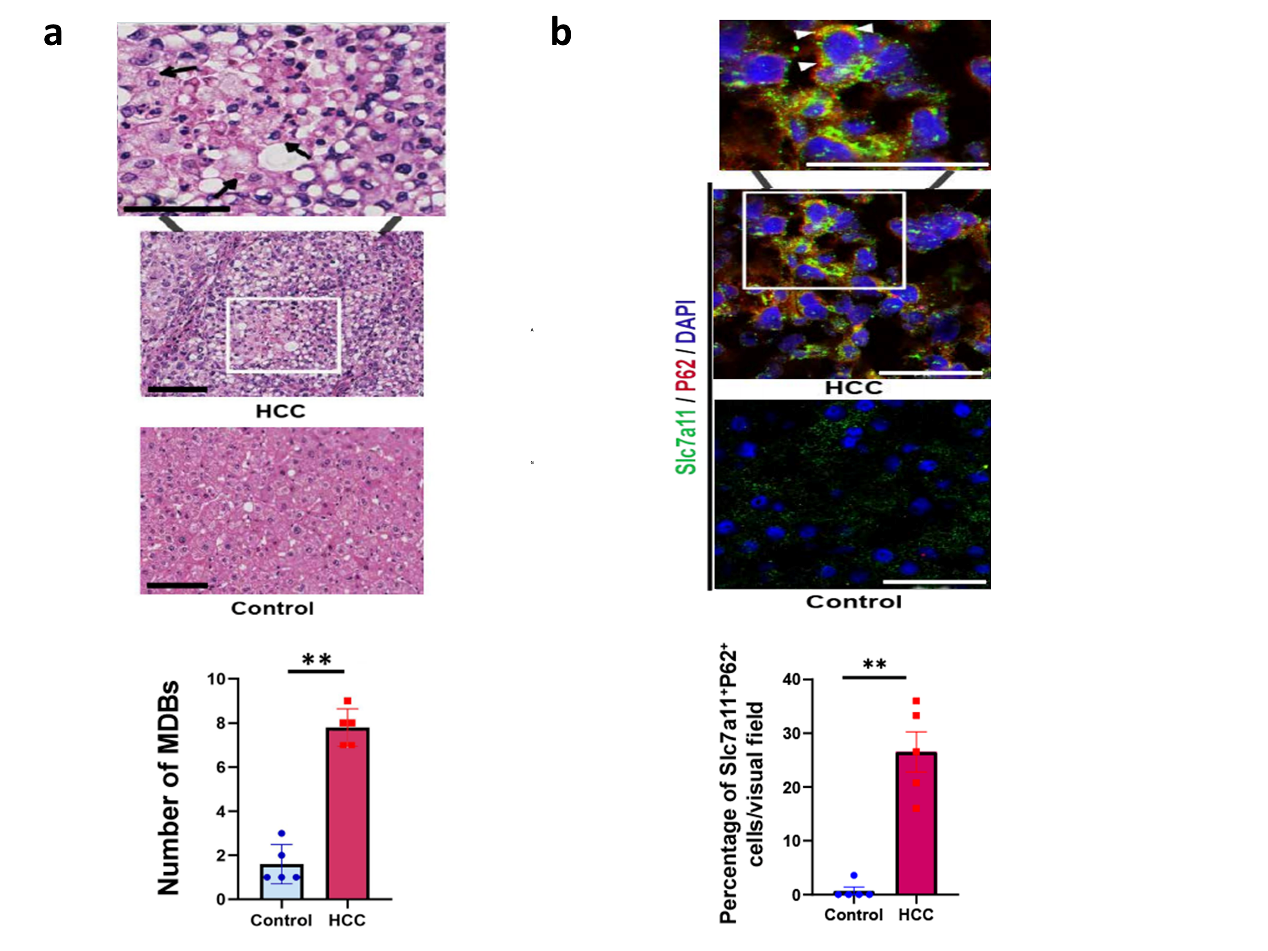
**

**Figure. S11.** H&E and Immunostaining analysis of Slc7a11 in HCC biopsies with MDBs

**(a)** H&E staining showing MDBs (arrows) in five HCC specimens. MDB numbers were quantified. Scale bar: 100 μm. (**b)** Immunofluorescent staining of positive Slc7a11 and p62 (arrows) in liver sections from Control and HCC samples. Quantification of Slc7a11⁺p62⁺ double-positive cells relative to DAPI⁺ nuclei in MDB-positive HCC tissues. Scale bar: 50 μm. Data are mean ± SEM (n=5). **p*<0.05; ***p*<0.01.


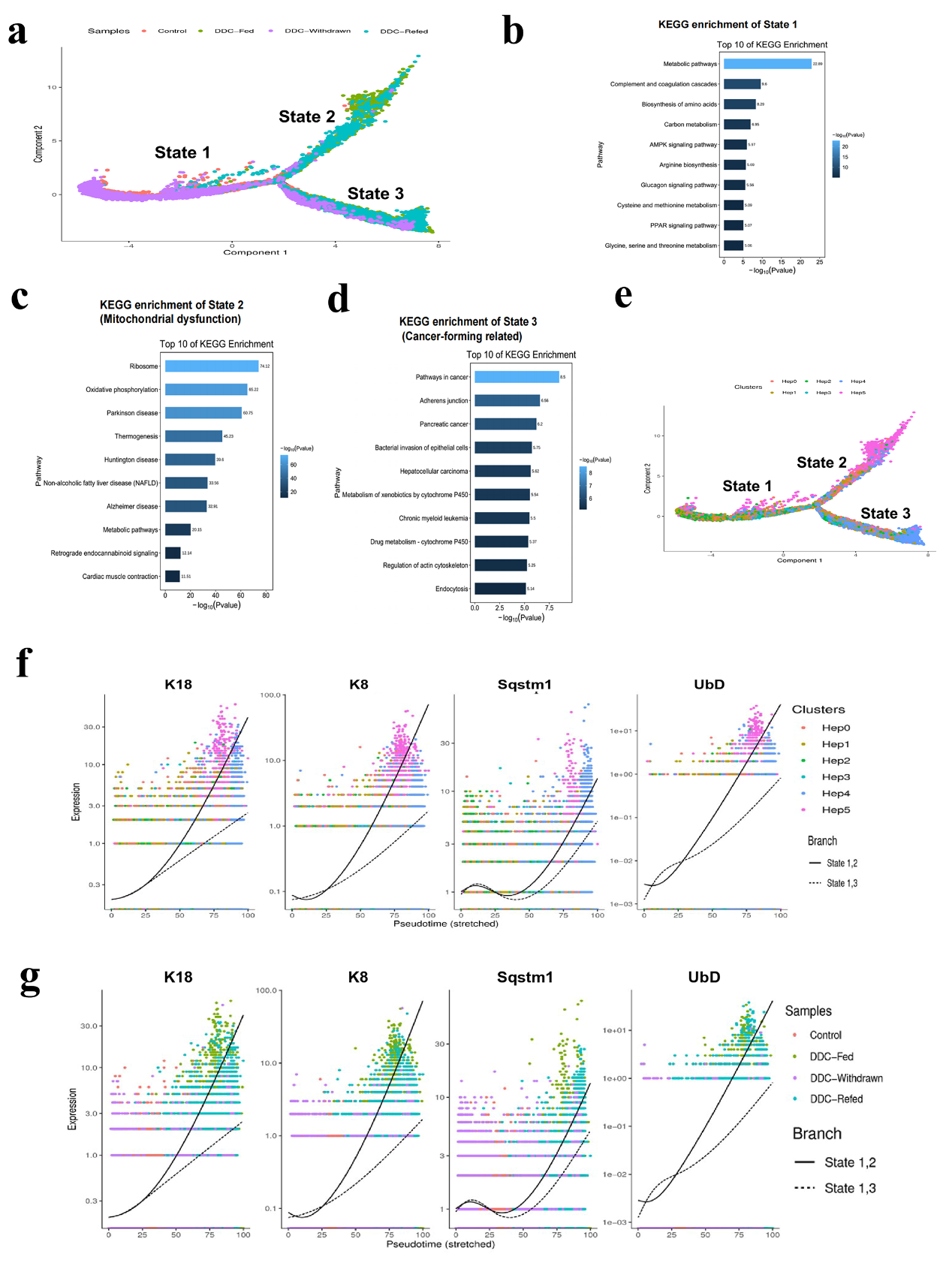


**Figure. S12.** Construction of the MDB-forming progenitor subtype differentiation trajectory in a pseudotime manners

**(a)** The Monocle trajectory plot showing the dynamics of DDC-treated and Controlled liver tissues and their pseudo-time curve across 4 groups hepatocytes. (**b-d)** KEGG analysis of each cell fate, including **(b)** Control and DDC-Withdrawn cells (State 1), **(c)** cell fate 2 branch (State 2, Mitochondrial dysfunction branch), and **(d)** cell fate 3 branch (State 3, Cancer-forming related branch). (**e)** The Monocle trajectory plot showing the dynamics of hepatocytes subclusters and their pseudo-time curve. (**f)** Spline plots showing the expression dynamics of K8/18, Sqstm1 and UbD from hepatic cluster pseudotime. (**g)** Spline plots showing the expression dynamics of K8/18, Sqstm1 and UbD from hepatic cluster pseudotime from DDC-treated and Controlled liver tissues.

**
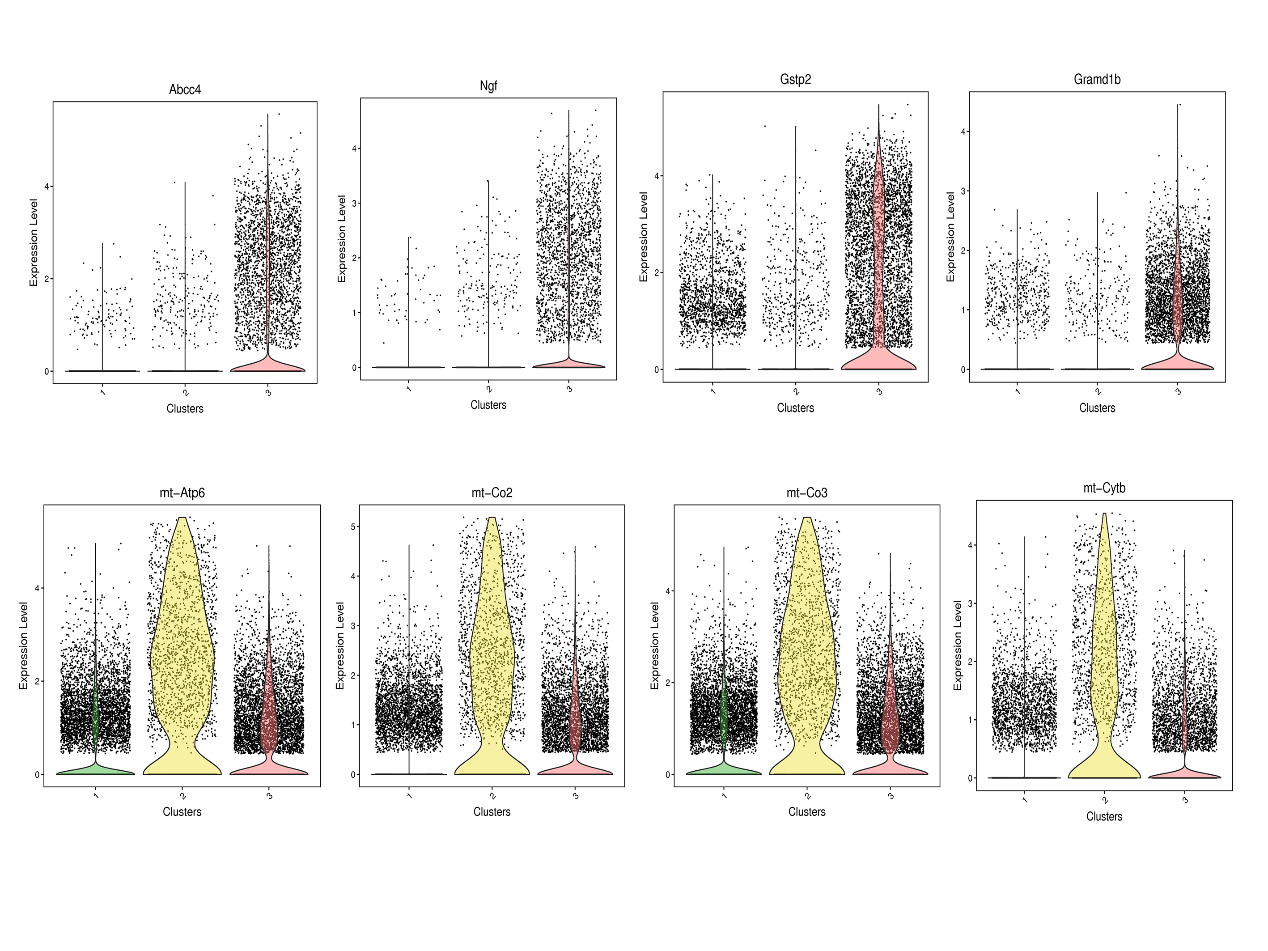
**

**Figure. S13.** Violin plots showing the expression level distributions of some top10 genes Abcc4 Ngf, Gstp2 and Gramd1b in Hep4 were upregulated in State 3, While Top10 genes mt-Atp6, mt-Co2, mt-Co3 and mt-Cytb were all upregulated in Hep5 in State 2.

**
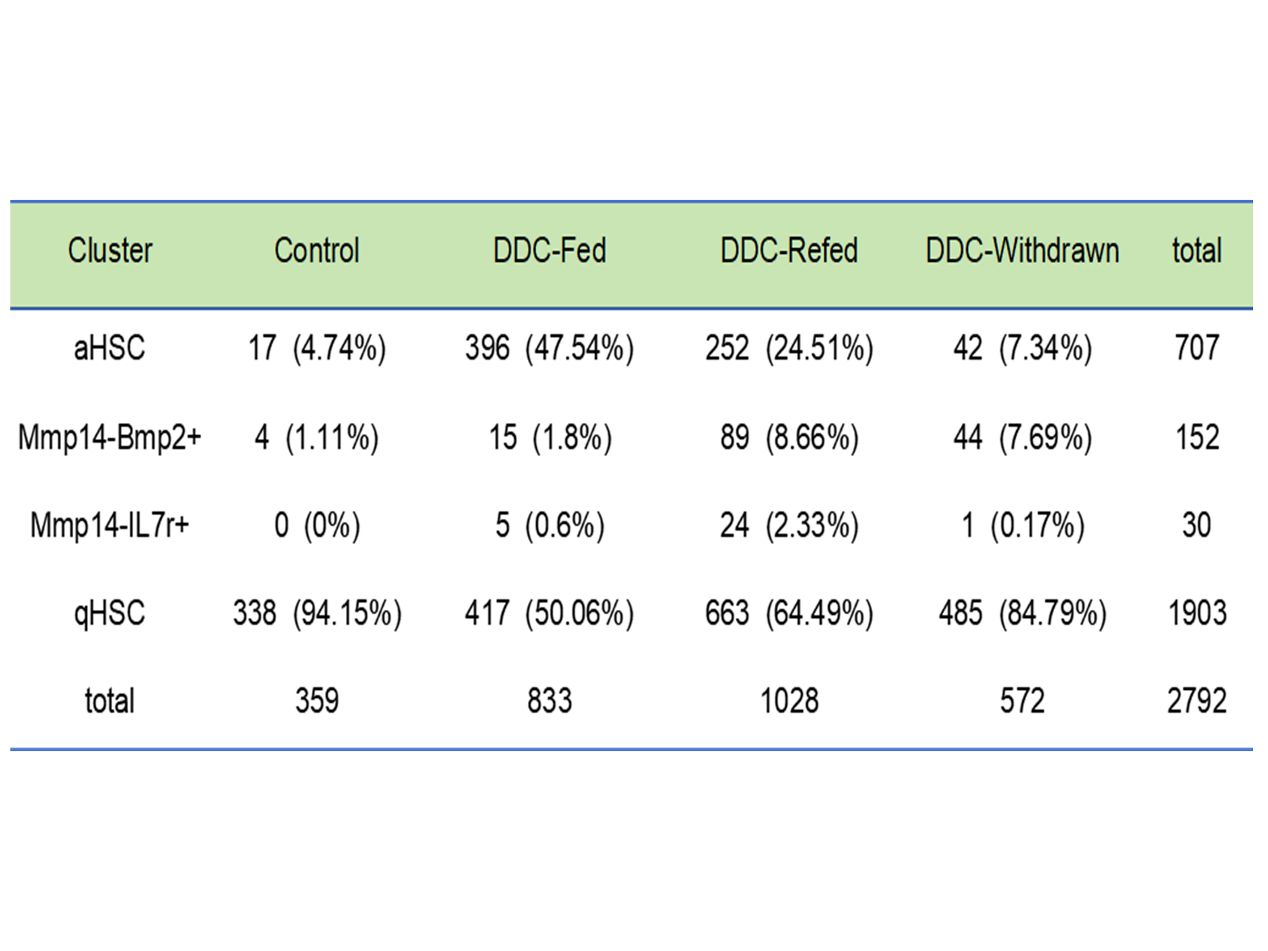
**

**Figure. S14.** Numbers of cells in each HSC subgroup across four groups.

**
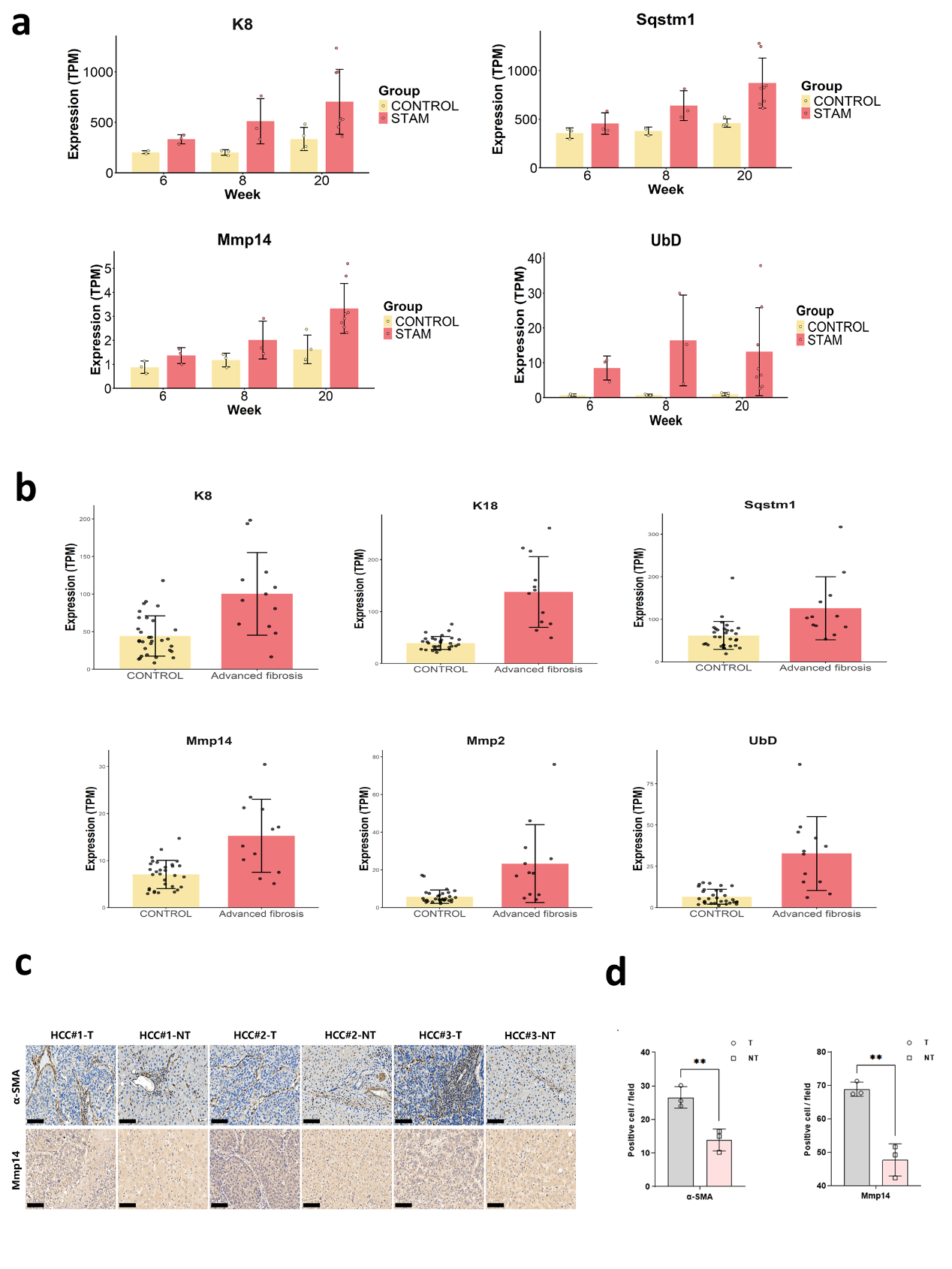
**

**Figure. S15.** Mmp14 association with liver fibrosis progression

**(a)** Expression of K8, Sqstm1, Mmp14, and UbD across fibrosis stages (GSE83596). **(b)** Expression of Mmp14, Mmp2, K8, K18, Sqstm1, and UbD in normal versus fibrotic livers (GSE162694). (**c)** IHC staining of α-SMA and Mmp14 in three HCC samples. Scale bar: 100 μm; magnification, 20×. (**d)** Quantification of IHC staining of Mmp14 and α-SMA. Data are mean ± SEM (n=3). ***p*<0.01.

**
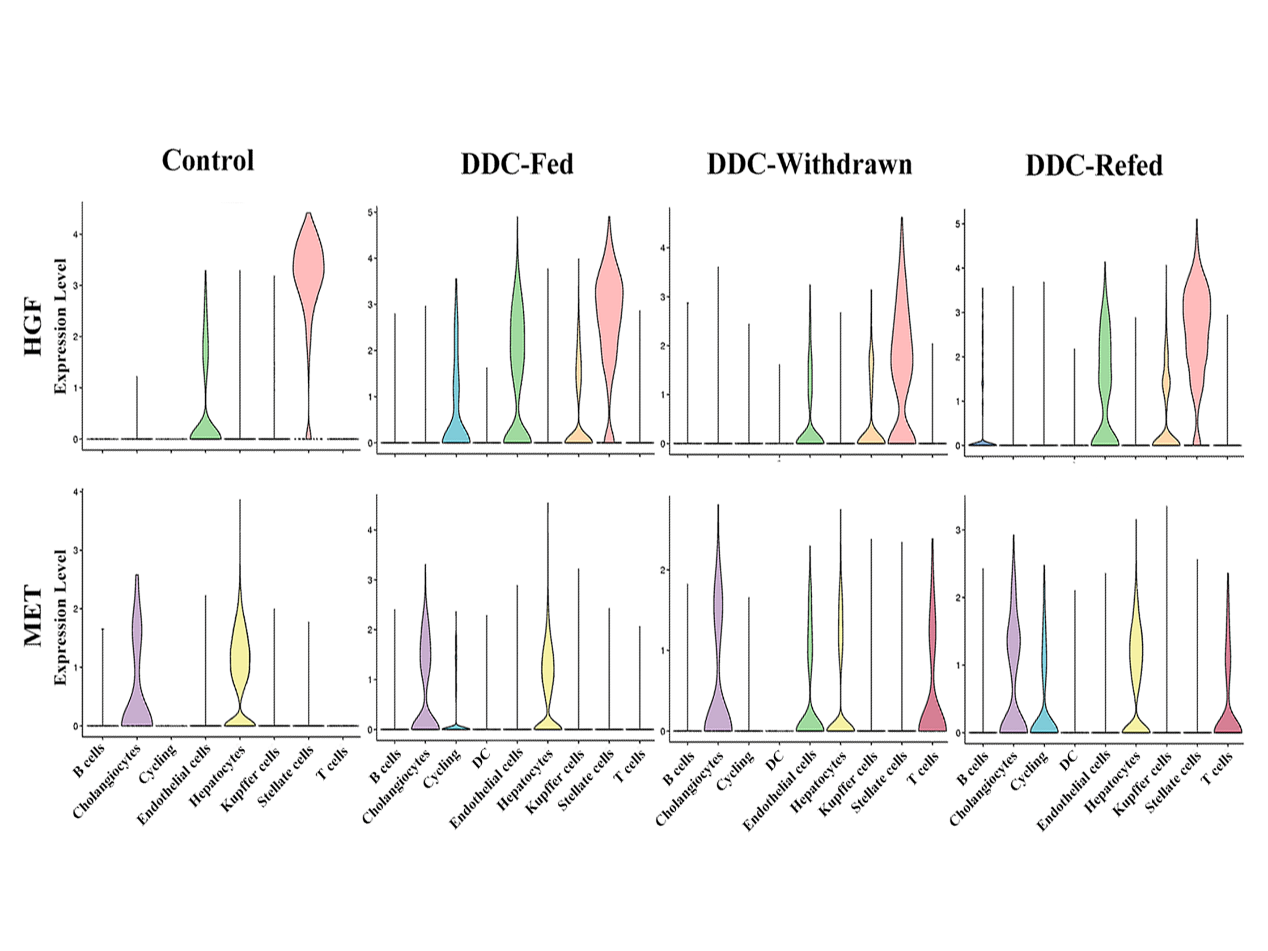
**

**Figure. S16.** Violin plots showing HGF and MET expression across four groups.

**
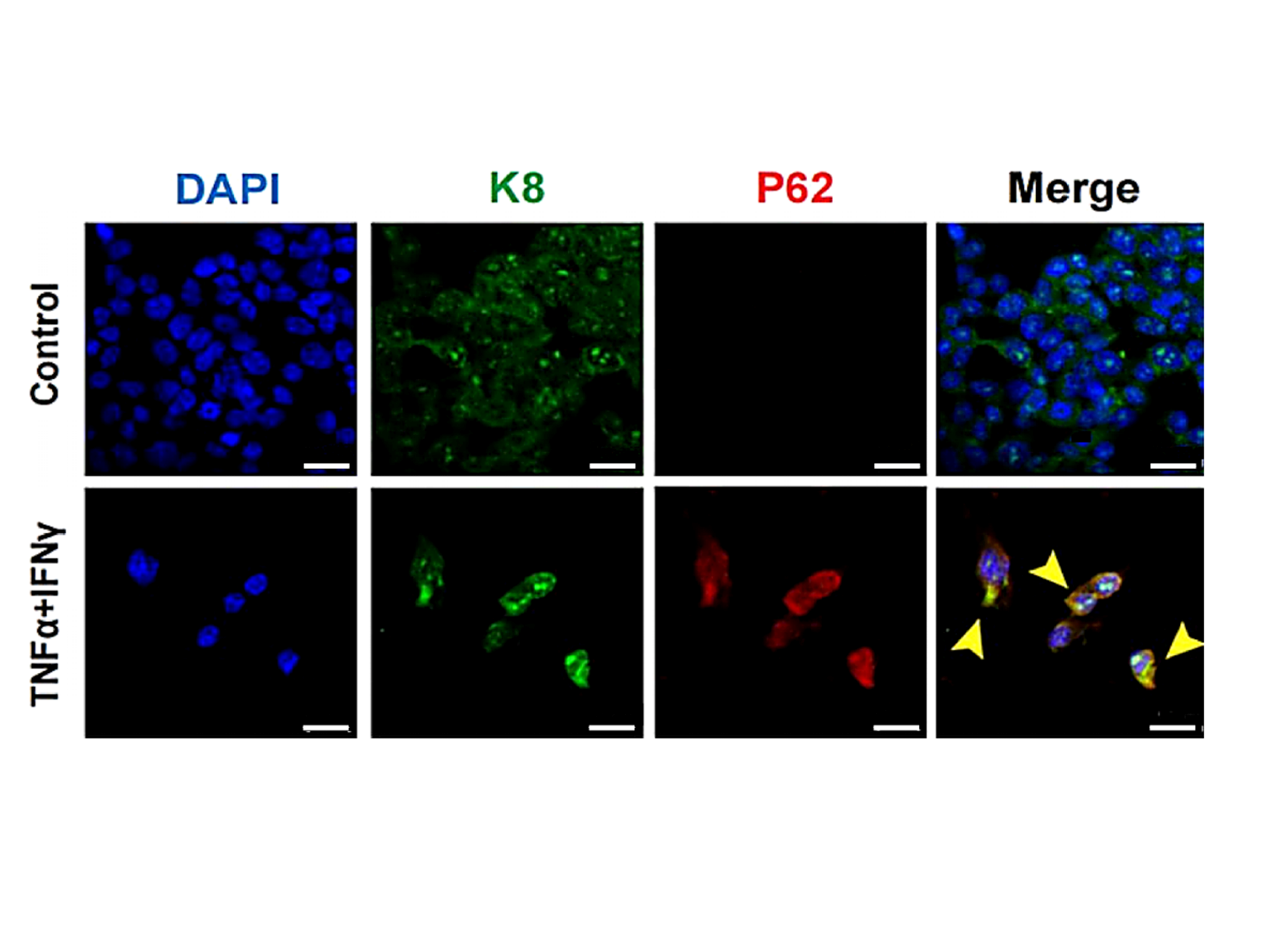
**

**Figure. S17.** Study of the formation of MDBs in Hepa1-6 cells treated continuously with a cotreatment of IFNγ and TNFα. The immunostaining is done with antibodies against endogenous K8 (green) with p62 (red). The immunoﬂuorescence labeling with antibodies against K8 and p62 (the major MDB constituents) for the detection of MDBs (arrow) was shown. The nuclear stain was DAPI (blue). Scale bar: 20 μm.

**
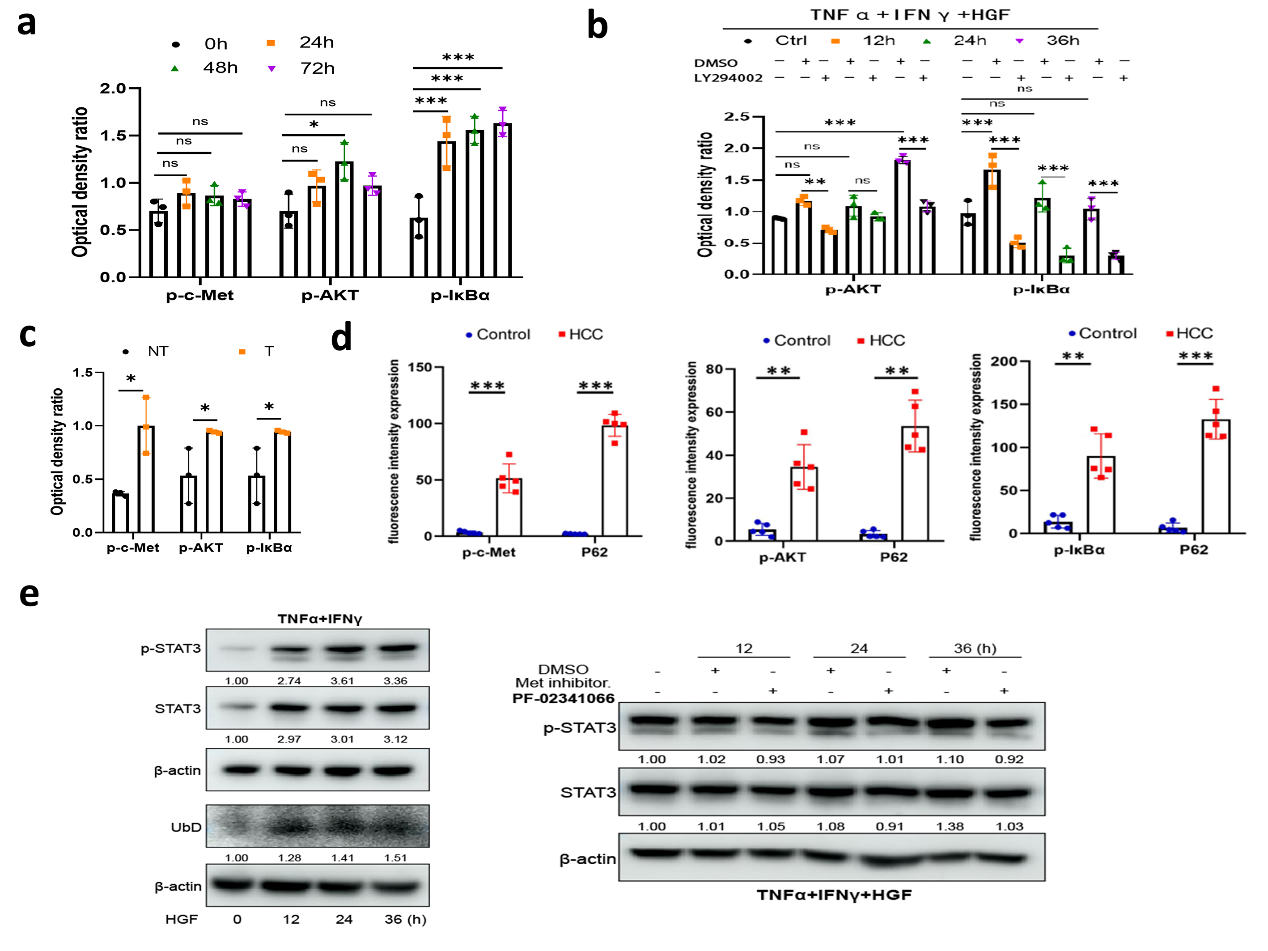
**

**Figure. S18.** HGF/MET axis drives MDB formation via NF-κB and STAT3 signaling (**a)** Quantification of total and phosphorylated MET, AKT, and IκBα in Hepa1-6 cells after TNFα/IFNγ treatment. (**b)** Quantification analysis of PI3K/AKT/NF-κB signaling in Hepa1-6 cells treated with HGF in the presence/absence of PI3K inhibitor (LY294002). (**c)** Quantification of signaling proteins in MDB-HCC tissues. (**d)** Densitometry of co-staining for p-c-MET, p-AKT, and p-IκB with p62 in MDB-positive HCC versus non-tumor tissues (n=5). (**e)** Western blot of p-STAT3, and UbD proteins in Hepa1-6 cells treated with HGF or MET inhibitors for 12–36h. Scale bar, 50 μm. Data are mean ± SEM (n=3 or 5). **p*<0.05; ***p*<0.01; ****p*<0.001; ns, no significance.


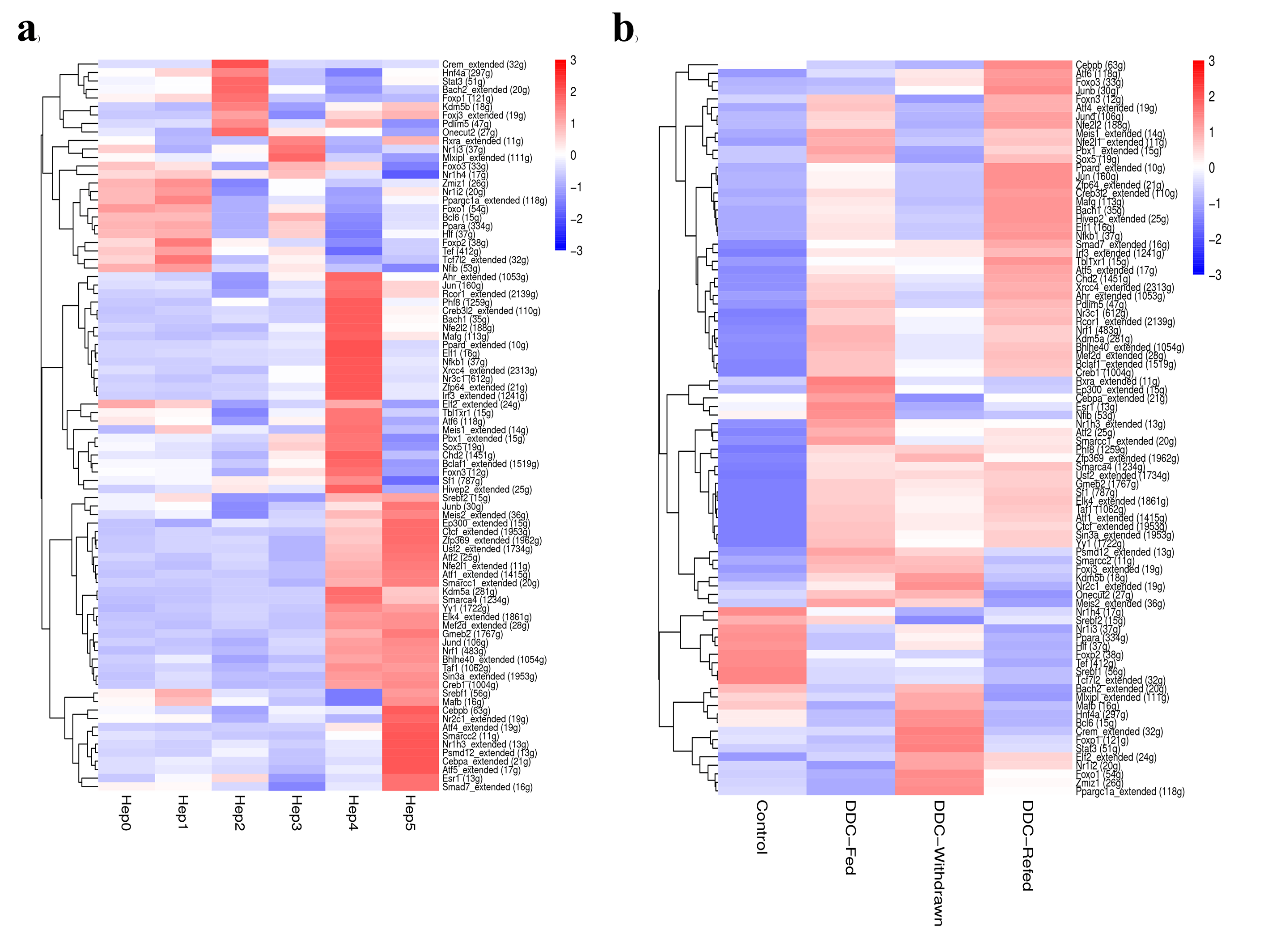


**Figure. S19.** Heatmap of transcription factor regulatory activity in hepatic subpopulations (**a**) and DDC-treated livers (**b**) estimated by SCENIC.

**
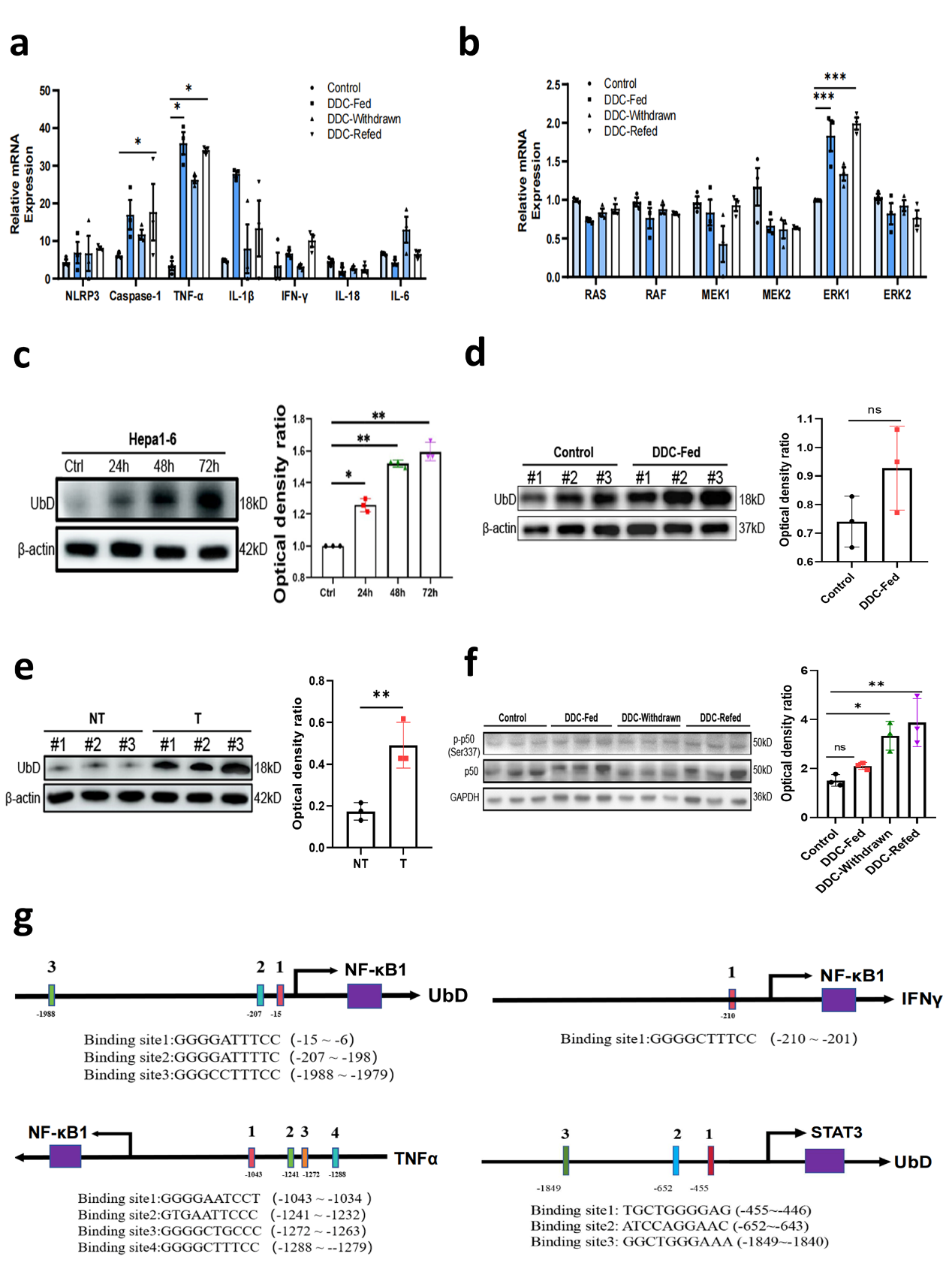
**

**Figure. S20.** HGF/MET signaling drives the proinflammatory response

**(a)** mRNA expression of IL-1β, IL-18, NLRP3, and Casp-1 in DDC versus Control livers (n=4). **(b)** mRNA expression of RAS, RAF, MEK1, MEK2, ERK1, and ERK2 in DDC versus Control livers (n=4). **(c-g)** UbD and NF-κB/STAT3 pathway regulation: **(c)** UbD in Hepa1-6 cells after TNFα/IFNγ treatment; **(d)** UbD in Control vs. DDC-fed mouse livers; **(e)** UbD in MDB-positive HCC vs. non-tumor tissues; **(f)** p-p50 and p50 in Control vs. DDC livers; **(g)** predicted NF-κB1 (p50) and STAT3 binding sites in TNFα, IFNγ, and UbD promoters. Data are mean ± SEM (n=3). **p*<0.05; ***p*<0.01; ****p*<0.001; ns, no significance.


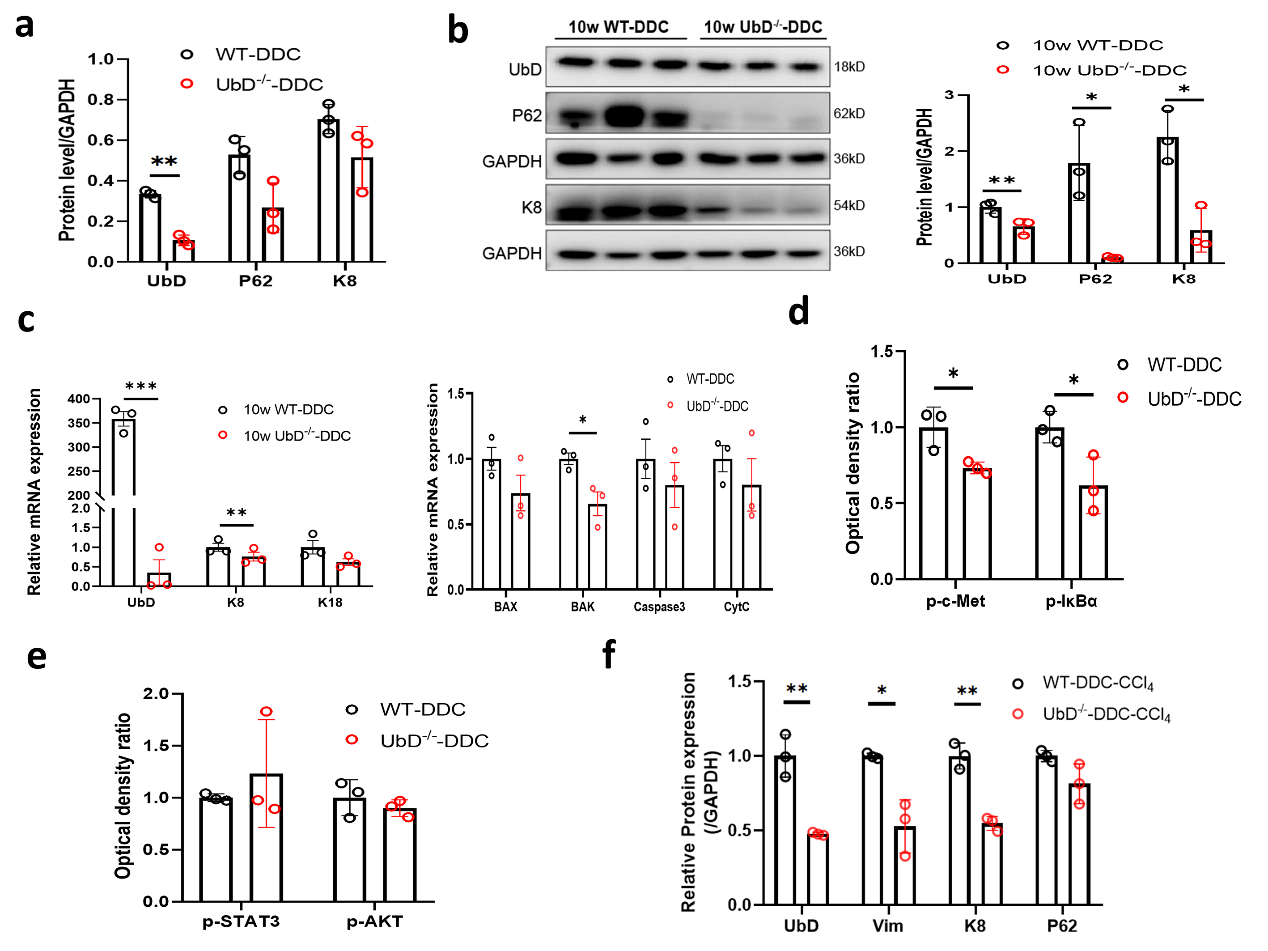


**Figure. S21.** MDB constitutes and apoptosis molecules analysis in UbD⁻/⁻ mice

**(a)** Quantification analysis of UbD, p62, and K8 in WT-DDC vs UbD⁻/⁻-DDC mice. (**b)** Western blot of UbD, p62, and K8 in WT-DDC and UbD⁻/⁻-DDC mice after 10 weeks of DDC feeding. (**c)** mRNA expression of MDB markers and pro-apoptotic genes in WT-DDC and UbD⁻/⁻-DDC mice. (**d-e)** Quantification analysis of p-MET, p-IκBα, p-AKT and p-STAT3 in WT-DDC vs UbD⁻/⁻-DDC mice. (**f)** Quantification analysis of MDB constitutes and fibrosis related molecule Vimentin in UbD⁻/⁻-DDC-CCl4 mice. Data are mean ± SEM (n=3). **p*<0.05; ***p*<0.01; ****p*<0.001.

**Supplementary tables**

Table S1. Liver, serum parameters, and body weight in DDC-induced MDB mouse model

| Experimental Group | C3H | | | |
| --- | --- | --- | --- | --- |
|  | Control | DDC-Fed | DDC-Withdrawn | DDC-Refed |
| Number of mice | 4 | 4 | 4 | 4 |
| Body weight | 31.07±1.38^A,a^ | 23,8±1.39^A,a;B,a^ | 28.8±1.41^B,a;C,c^ | 27.87±1.17^C,c^ |
| ALT (U/L) | 51.59±13.48^A,b^ | 318.54±68.41^A,b;B,a^ | 118.5±38^B,a;C,c^ | 1955.11±889.71^C,c^ |
| AST (U/L) | 112.02±11.96^A,b^ | 311.14±47.74^A,b;B,a^ | 157.25±40.46^B,a;C,c^ | 1904.96±718.43^C,c^ |
| ALP (U/L) | 31.96±10.74^A,c^ | 187.31±72.07^A,c;B,a^ | 65.76±27.88^B,a;C,c^ | 336.29±173.73^C,c^ |
| Total bilirubin (Umol /L) | 215.52±45.96^A,a^ | 50.5±14.6^A,a;B,c^ | 18.04±11.02^B,c;C,c^ | 14.67±4.52^C,c^ |
| Ductular reaction | 0.33±0.58^A,c^ | 1.67±1.15^A,c;B,c^ | 1^B,c^ | 1 |
| Periductal fibrosis | 0^A,c^ | 0.67±0.58^A,c;B,c^ | 0.67±0.58^B,c;C,c^ | 1^C,c^ |
| Hepatocyte ballooning | 0^A,c^ | 2±1^A,c;B,c^ | 2^B,c^ | 3 |
| Steatosis | 0^A,c^ | 1.33±0.58^A,c;B,c^ | 1^B,c^ | 1 |
| Inflammation | 0.33±0.58^A,c^ | 1.33±0.58^A,c;B,c^ | 1^B,c^ | 1 |
| MDB formation | 0^A,c^ | 2.33+1.15^A,c;B,c^ | 0.33+0.58^B,c;C,c^ | 1^C,c^ |

Control, control group without DDC; DDC-Fed, experimental group with DDC; DDC-Withdrawn, experimental group with DDC withdraw for 1month; DDC-Refed, experimental group with DDC after withdrawn for 1week; See 10 fields of view under 20×lenses whether there are MDBs < 5, it is “1”; 5-10 is “2”; >=10 is “3”. Values are expressed as mean ± SEM. P values (Student t test); A: Control vs DDC-Fed; B: DDC-Fed vs DDC-Withdrawn; C: DDC-Withdrawn vs DDC-Refed; aP, p<0.05; bP, p<0.01; cP, p=ns

Table S2. Liver and serum parameters in five HCC samples with MDB formation

Human Sample

|  | HCC#1 | HCC#2 | HCC#3 | HCC#4 | HCC#5 |
| --- | --- | --- | --- | --- | --- |
| Differentiation severity | mild | low | mild | mild | mild |
| Gender | female | male | male | female | male |
| Liver volume (cm^3^) | 756 | 1268 | 1320 | 490 | 270 |
| Age (year) | 44 | 62 | 48 | 62 | 59 |
| Weight (kg) | 47 | 62 | 64 | 57 | 78 |
| ALT (U/L) | 20 | 37 | 11 | 21 | 31 |
| AST (U/L) | 29 | 29 | 20 | 33 | 33 |
| GGT (U/L) | 21 | 64 | 59 | 25 | 24 |
| ALP (U/L) | 74 | 80 | 108 | 103 | 81 |
| TBIL (U/L) | 2.9 | 17.3 | 14 | 16.9 | 24.6 |
| AFP (U/L) | >1210 | >1210 | 905 | 41.4 | 569 |
| HBV-DNA (IU/mL) | 77100 | 8190 | 9200 | 2300000 | 1940 |
| CK19 | - | + | + | + | - |
| CK8/18 | + | + | + | + | + |
| MDBs | +++ | +++ | +++ | +++ | ++ |

^1^ALT, alanine transaminase; ^2^AST, aspartate transaminase; ^3^GGT, gamma-glutamyl transferase; ^4^ALP, alkaline phosphatase; ^5^TBIL, total bilirubin; ^6^AFP, alpha fetoprotein; ^7^HBV, hepatitis B virus; ^8^CK19, cytokeratin-19; ^9^CK8/18, cytokeratin-8/18; ^10^See 10 fields of view under 20x lenses whether there are MDBs < 5, it is +; 5-10 is ++; >=10 is +++

Table S3. Primers for RT‒qPCR

| Gene name  （Mouse） | Accession Number | Sequences of primers |
| --- | --- | --- |
| GAPDH | NM_008084 | Forward Primer 5’-TGCTGAGTATGTCGTGGAG-3’  Reverse Primer 5’-TGTCATATTTCTCGTGGTTC-3’ |
| UbD (FAT10) | NM_023137 | Forward Primer 5’-GATTGACAAGGAAACCACTATCCA-3’  Reverse Primer 5’-ACAAGGGCAGCTCTTCATCAC -3’ |
| K8 | NM_031170 | Forward Primer 5’-TCCATCAGGGTGACTCAGAAA-3’  Reverse Primer 5’-CCAGCTTCAAGGGGCTCAA-3’ |
| K18 | NM_010664 | Forward Primer 5’-CAGCCAGCGTCTATGCAGG-3’  Reverse Primer 5’-CCTTCTCGGTCTGGATTCCAC-3’ |
| Sqstm1(p62) | NM_011018 | Forward Primer 5’-AAGTCAGCAAACCTGACG-3’  Reverse Primer 5’-CCATCTGTTCCTCTGGCT-3’ |
| HGF | [NM_001289458](https://www.ncbi.nlm.nih.gov/nuccore/NM_001289458.1) | Forward Primer 5’-ATGTGGGGGACCAAACTTCTG-3’  Reverse Primer 5’-GGATGGCGACATGAAGCAG-3’ |
| Slc7a11 | NM_011990 | Forward Primer 5’-GGCACCGTCATCGGATCAG-3’  Reverse Primer 5’-CTCCACAGGCAGACCAGAAAA-3’ |
| MET | NM_001410120 | Forward Primer 5’-TCACCATCTATCTGGGATCTCG-3’  Reverse Primer 5’-CCCCGAAGACGATTGAGCTAA-3’ |
| STAT3 | NM_011486 | Forward Primer 5’-CAATACCATTGACCTGCCGAT-3’  Reverse Primer 5’-GAGCGACTCAAACTGCCCT-3’ |
| TGFβ1 | NM_011577 | Forward Primer 5’-CTCCCGTGGCTTCTAGTGC-3’  Reverse Primer 5’-GCCTTAGTTTGGACAGGATCTG-3’ |
| Acta2 | NM_007392 | Forward Primer 5’-GTCCCAGACATCAGGGAGTAA-3’  Reverse Primer 5’-TCGGATACTTCAGCGTCAGGA-3’ |
| CDH1 | NM_009864 | Forward Primer 5’-CAGGTCTCCTCATGGCTTTGC-3’  Reverse Primer 5’-CTTCCGAAAAGAAGGCTGTCC-3’ |
| CDH2 | NM_007664 | Forward Primer 5’-AGCGCAGTCTTACCGAAGG-3’  Reverse Primer 5’-TCGCTGCTTTCATACTGAACTTT-3’ |
| Vim | NM_011701 | Forward Primer 5’-CGGCTGCGAGAGAAATTGC-3’  Reverse Primer 5’-CCACTTTCCGTTCAAGGTCAAG-3’ |
| Mmp14 | NM_008608 | Forward Primer 5’-CAGTATGGCTACCTACCTCCAG-3’  Reverse Primer 5’-GCCTTGCCTGTCACTTGTAAA-3’ |
| Mmp2 | NM_008610 | Forward Primer 5’-CAAGTTCCCCGGCGATGTC-3’  Reverse Primer 5’-TTCTGGTCAAGGTCACCTGTC-3’ |
| Col1a1 | NM_007742 | Forward Primer 5’-GCTCCTCTTAGGGGCCACT-3’  Reverse Primer 5’-CCACGTCTCACCATTGGGG-3’ |
| Col3a1 | NM_009930 | Forward Primer 5’-ACGTAGATGAATTGGGATGCAG-3’  Reverse Primer 5’-GGGTTGGGGCAGTCTAGTG-3’ |
| Col4a1 | NM_009931 | Forward Primer 5’-CTGGCACAAAAGGGACGAG-3’  Reverse Primer 5’-ACGTGGCCGAGAATTTCACC-3’ |
| Slpi | NM_001412601 | Forward Primer 5’-GGCCTTTTACCTTTCACGGTG-3’  Reverse Primer 5’-TACGGCATTGTGGCTTCTCAA-3’ |
| Ngf | NM_001112698 | Forward Primer 5’-CCAGTGAAATTAGGCTCCCTG-3’  Reverse Primer 5’-CCTTGGCAAAACCTTTATTGGG-3’ |
| Rps19 | NM_001360115 | Forward Primer 5’-CAGCAGGAGTTCGTCAGAGC-3’  Reverse Primer 5’-CACCCATTCGGGGACTTTCA-3’ |
| Mt-ND5 | NP_904338 | Forward Primer 5’-AACTTTACTGGGGTTTTTC-3’  Reverse Primer 5’-GTTGAGGTGGATTTTGGGA-3’ |
| Mt-ND4 | NP_904337 | Forward Primer 5’-CCTTCATCCTTCTCTCCCT-3’  Reverse Primer 5’-TTATTAGTATTGTTGCTCC-3’ |
| Mt-Co3 | NP_904334 | Forward Primer 5’-AACGAAACCACATAAATCA-3’  Reverse Primer 5’-GTAGGCAAACAATAAGGAA-3’ |
| Gab2 | NM_001162477 | Forward Primer 5’-AATCGCCTCCCGAGAAGAAGT-3’  Reverse Primer 5’-ACAAGTTCAGGTTGATGATCCG-3’ |
| AKT | NM_011785 | Forward Primer 5’-TGGGTTCAGAAGAGGGGAGAA-3’  Reverse Primer 5’-AGGGGATAAGGTAAGTCCACATC-3’ |
| NF-κB1 (p50) | NM_001410442 | Forward Primer 5’-ATGGCAGACGATGATCCCTAC-3’  Reverse Primer 5’-TGTTGACAGTGGTATTTCTGGTG-3’ |
| LMP7 | NM_010724 | Forward Primer 5’-GGCTCTCGGGACAGATGTTTT-3’  Reverse Primer 5’-ACCACTGTCCATCACCCCATA-3’ |
| LMP2 | NM_013585 | Forward Primer 5’-TGGAGCTACACGGGTTGGA -3’  Reverse Primer 5’-GAGATGTTCTTCACCACGTTTGC -3’ |
| Mecl-1 | NM_013585 | Forward Primer 5’-TTGTGTTCCGAGATGGAGTCAT -3’  Reverse Primer 5’-GCCACAACCGAATCGTTAGTG-3’ |
| RAS | [NM_001130444](http://www.ncbi.nlm.nih.gov/entrez/query.fcgi?cmd=Search&db=Nucleotide&term=NM_001130444) | Forward Primer:5’CGTGAGATTCGGCAGCATAAA 3’  Reverse Primer:5’GACAGCACACATTTGCAGCTC 3’ |
| RAF | [NM_029780](http://www.ncbi.nlm.nih.gov/entrez/query.fcgi?cmd=Search&db=Nucleotide&term=NM_029780) | Forward Primer:5’TGGACTCAAAGATGCGGTGTT 3’  Reverse Primer:5’ AAAACCCGGATAGTATTGCTTGT3’ |
| MEK1 | [NM_008927](http://www.ncbi.nlm.nih.gov/entrez/query.fcgi?cmd=Search&db=Nucleotide&term=NM_008927) | Forward Primer:5’AAGGTGGGGGAACTGAAGGAT 3’  Reverse Primer:5’CGGATTGCGGGTTTGATCTC 3’ |
| MEK2 | [NM_023138](http://www.ncbi.nlm.nih.gov/entrez/query.fcgi?cmd=Search&db=Nucleotide&term=NM_023138) | Forward Primer:5’GTTACCGGCACTCACTATCAAC 3’  Reverse Primer:5’CCTCCAGCCGCTTCCTTTG 3’ |
| ERK1 | [NM_011952](http://www.ncbi.nlm.nih.gov/entrez/query.fcgi?cmd=Search&db=Nucleotide&term=NM_011952) | Forward Primer:5’TCCGCCATGAGAATGTTATAGGC 3’  Reverse Primer:5’GGTGGTGTTGATAAGCAGATTGG 3’ |
| ERK2 | [NM_001038663](http://www.ncbi.nlm.nih.gov/entrez/query.fcgi?cmd=Search&db=Nucleotide&term=NM_001038663) | Forward Primer:5’GGTTGTTCCCAAATGCTGACT 3’  Reverse Primer:5’CAACTTCAATCCTCTTGTGAGGG 3’ |
| IL-18 | NM_008360 | Forward Primer:5’GACTCTTGCGTCAACTTCAAGG 3’  Reverse Primer:5’CAGGCTGTCTTTTGTCAACGA 3’ |
| IFNγ | NM_008337 | Forward Primer:5’ATGAACGCTACACACTGCATC 3’  Reverse Primer:5’CCATCCTTTTGCCAGTTCCTC 3’ |
| NLRP3 | NM_145827 | Forward Primer:5’ATTACCCGCCCGAGAAAGG 3’  Reverse Primer:5’TCGCAGCAAAGATCCACACAG 3’ |
| Caspase-1 | NM_009807 | Forward Primer:5’ACAAGGCACGGGACCTATG 3’  Reverse Primer:5’TCCCAGTCAGTCCTGGAAATG 3’ |
| TNFα | NM_013693 | Forward Primer:5’CCCTCACACTCAGATCATCTTCT 3’  Reverse Primer:5’GCTACGACGTGGGCTACAG 3’ |
| IL-1β | NM_008361 | Forward Primer:5’GCAACTGTTCCTGAACTCAACT 3’  Reverse Primer:5’ATCTTTTGGGGTCCGTCAACT 3’ |
| IL-10 | NM_010548 | Forward Primer 5’-GCTCTTACTGACTGGCATGAG-3’  Reverse Primer 5’-CGCAGCTCTAGGAGCATGTG-3’ |
| IL-6 | NM_031168 | Forward Primer:5’TAGTCCTTCCTACCCCAATTTCC 3’  Reverse Primer:5’TTGGTCCTTAGCCACTCCTTC 3’ |

Table S4. Prediction binding site of NF-κB1 (p50) in TNFα, IFNγ and UbD promoter region


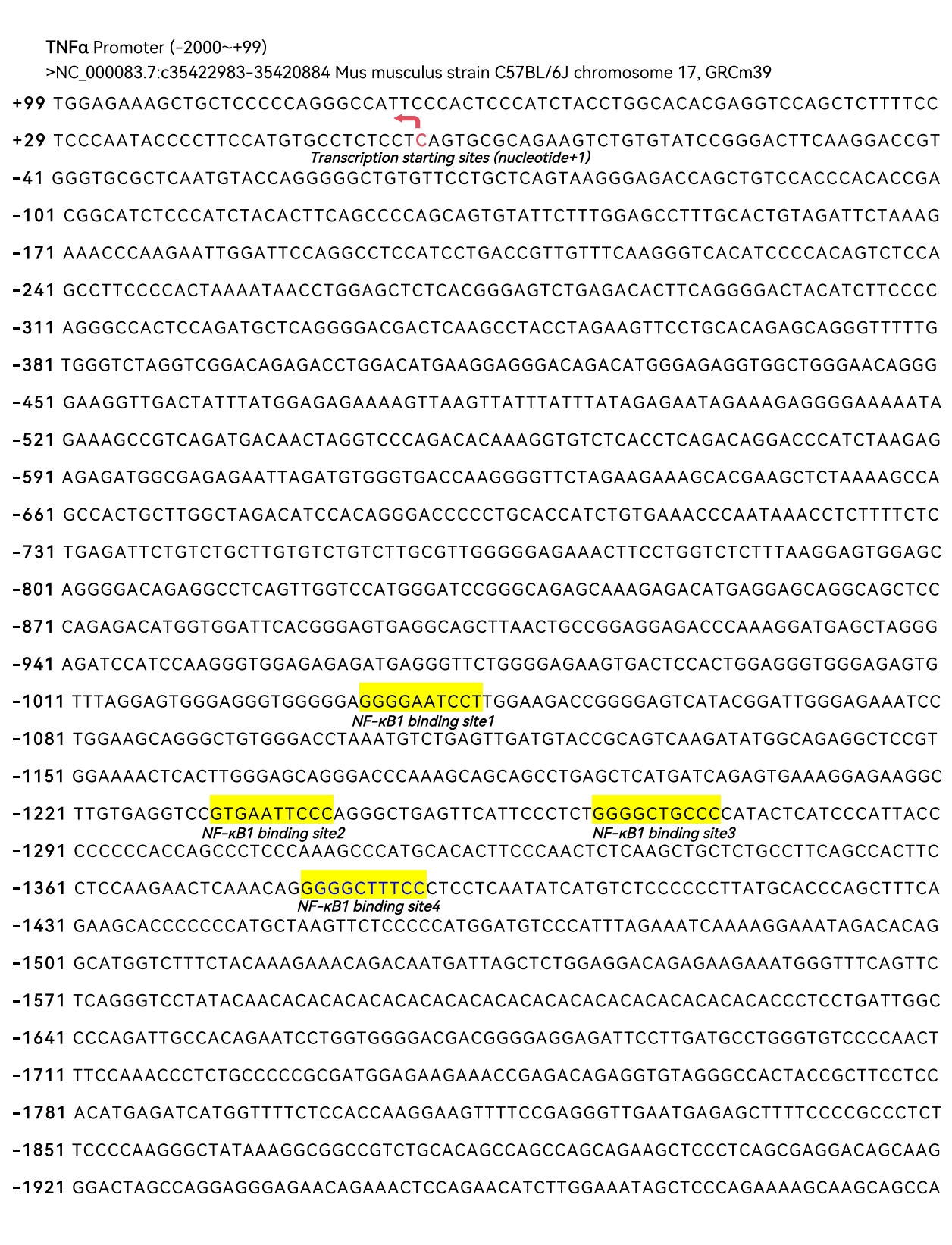


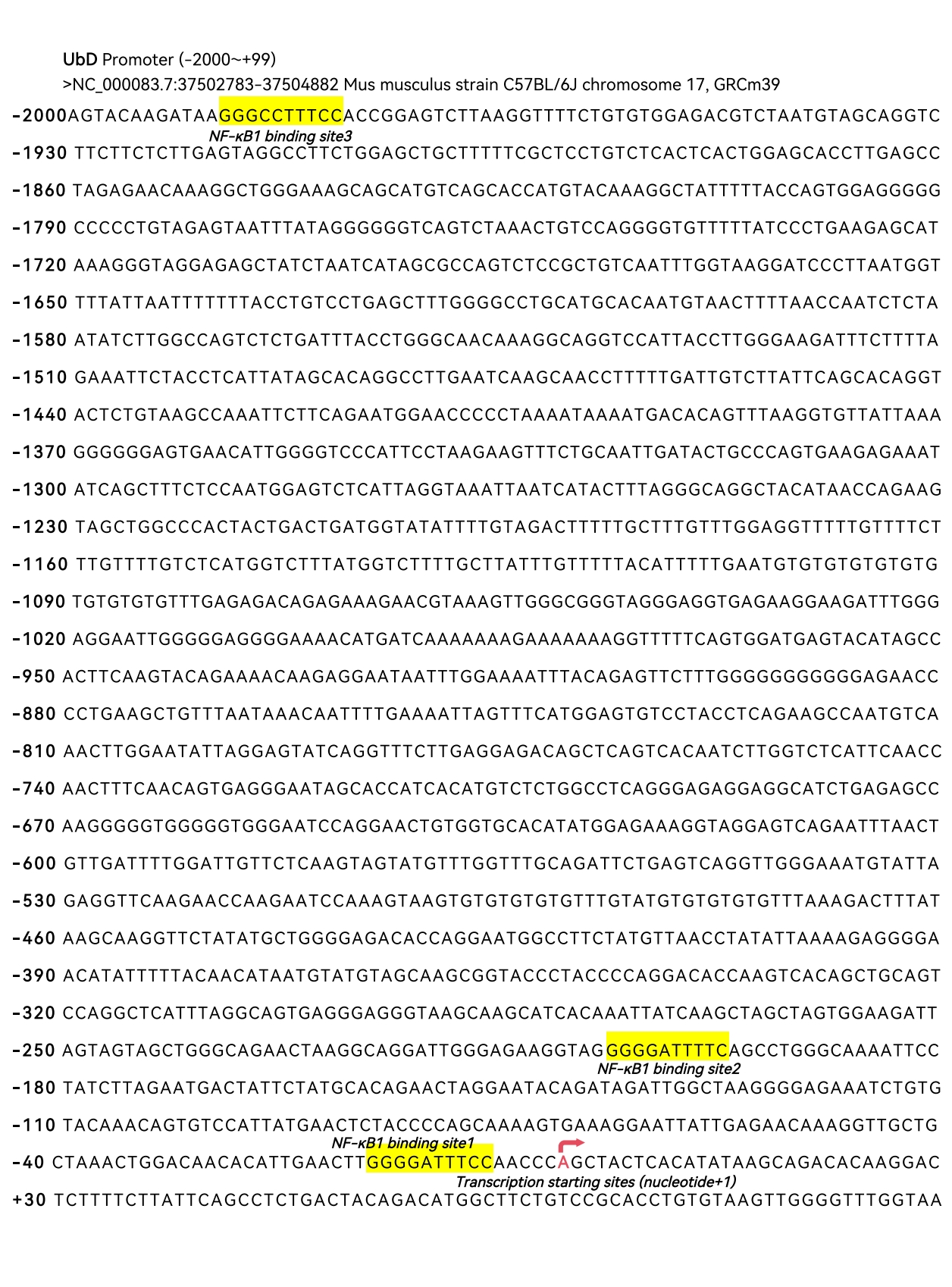


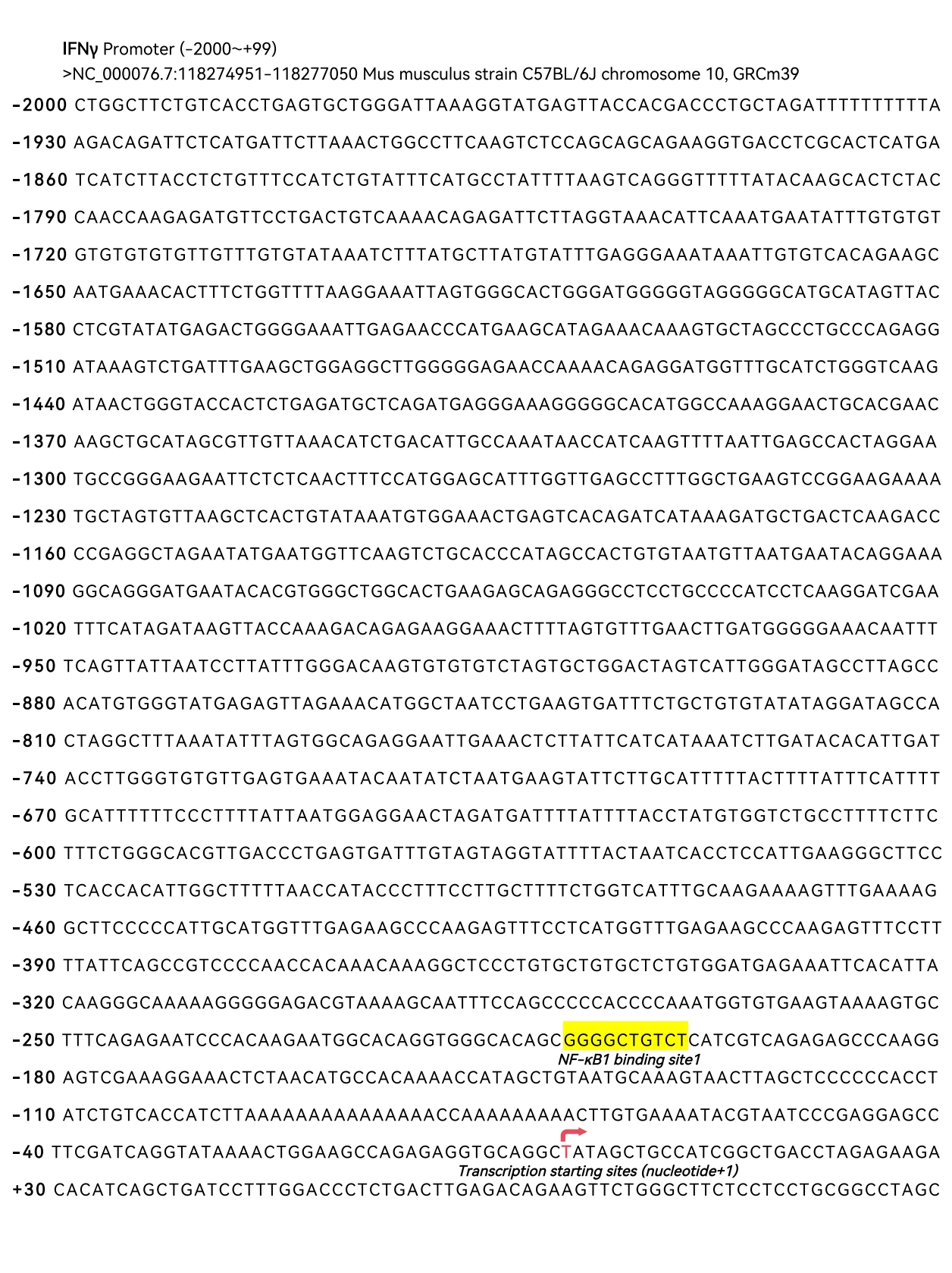


**REFERENCES**

1. Mattos, A., de Jager-Krikken, A., de Haan, M., Beljaars, L. & Poelstra, K. PEGylation of interleukin-10 improves the pharmacokinetic profile and enhances the antifibrotic effectivity in CCl(4)-induced fibrogenesis in mice. *J Control Release* **162**, 84-91 (2012).

2. Ma, Y., Zhang, L. & Huang, X. Genome modification by CRISPR/Cas9. *FEBS J* **281**, 5186-5193 (2014).

3. Fang, Z. et al. Single-cell transcriptomic analysis reveals characteristic feature of macrophage reprogramming in liver Mallory-Denk bodies pathogenesis. *J Transl Med* **23**, 77 (2025).

4. Velmeshev, D. et al. Single-cell genomics identifies cell type-specific molecular changes in autism. *Science* **364**, 685-689 (2019).

5. Chung, N.C. & Storey, J.D. Statistical significance of variables driving systematic variation in high-dimensional data. *Bioinformatics* **31**, 545-554 (2015).

6. Trapnell, C. et al. The dynamics and regulators of cell fate decisions are revealed by pseudotemporal ordering of single cells. *Nat Biotechnol* **32**, 381-386 (2014).

7. Efremova, M., Vento-Tormo, M., Teichmann, S.A. & Vento-Tormo, R. CellPhoneDB: inferring cell-cell communication from combined expression of multi-subunit ligand-receptor complexes. *Nat Protoc* **15**, 1484-1506 (2020).

8. Gulati, G.S. et al. Single-cell transcriptional diversity is a hallmark of developmental potential. *Science* **367**, 405-411 (2020).

9. Kenny, P.A. InferAMP, a python web app for copy number inference from discrete gene-level amplification signals noted in clinical tumor profiling reports. *F1000Res* **8**, 807 (2019).

10. Jew, B. et al. Accurate estimation of cell composition in bulk expression through robust integration of single-cell information. *Nat Commun* **11**, 1971 (2020).

11. Oliva, J., Bardag-Gorce, F., Lin, A., French, B.A. & French, S.W. The role of cytokines in UbD promoter regulation and Mallory-Denk body-like aggresomes. *Exp Mol Pathol* **89**, 1-8 (2010).

**Clinical information**

Human formalin-fixed paraffin-embedded (FFPE) liver biopsies from patients with HCC were obtained from the Affiliated Qingyuan Hospital Archives of Guangzhou Medical University (Qingyuan, China), with Institutional Review Board approval (IRB-2022-094), which is registered at Medicalresearch.org.cn (ChiCTR2400084248)

**Informed consent of HCC1**

**
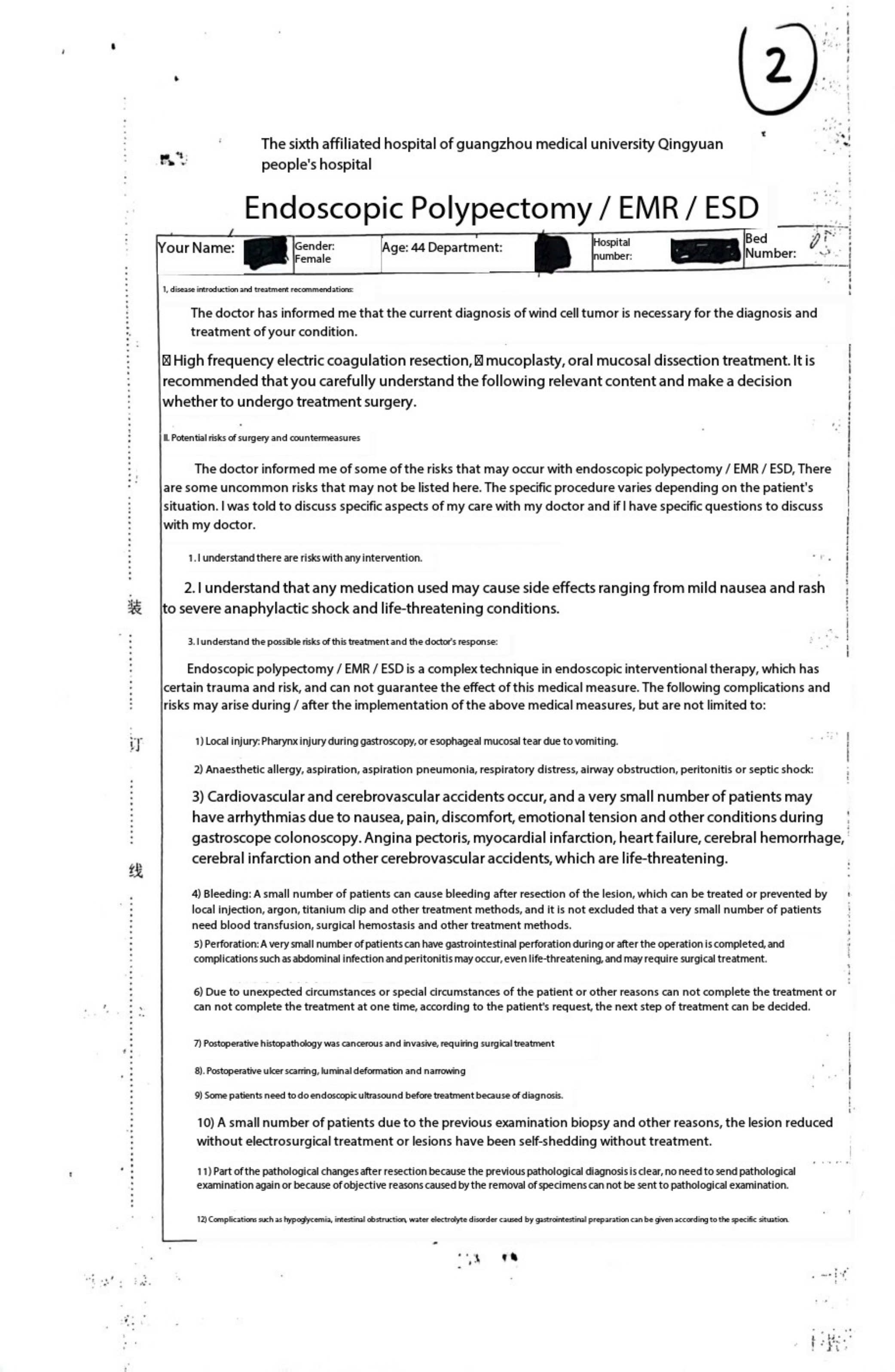

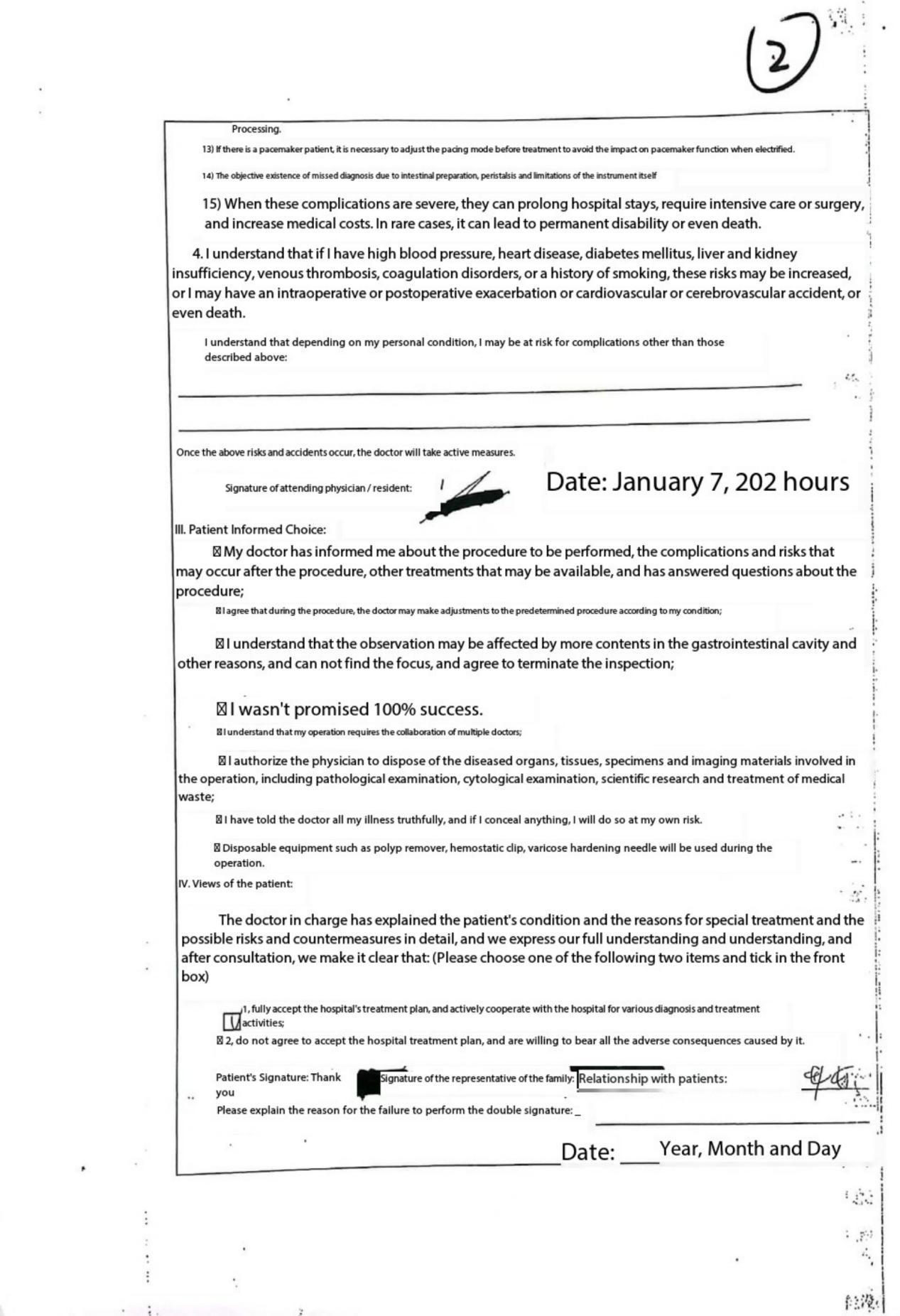
**

**Informed consent of HCC2**

**
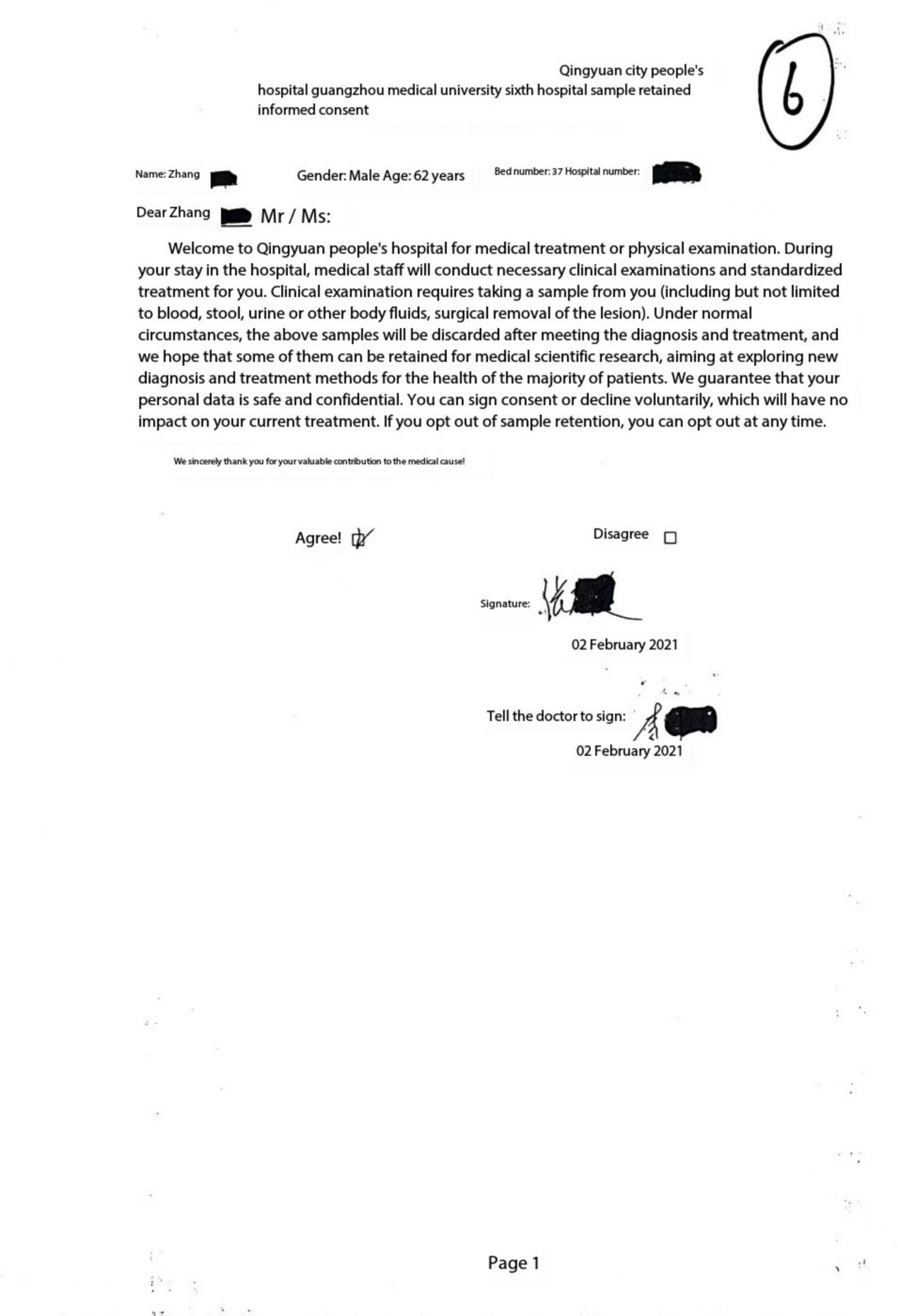
**

**Informed consent of HCC3**

**
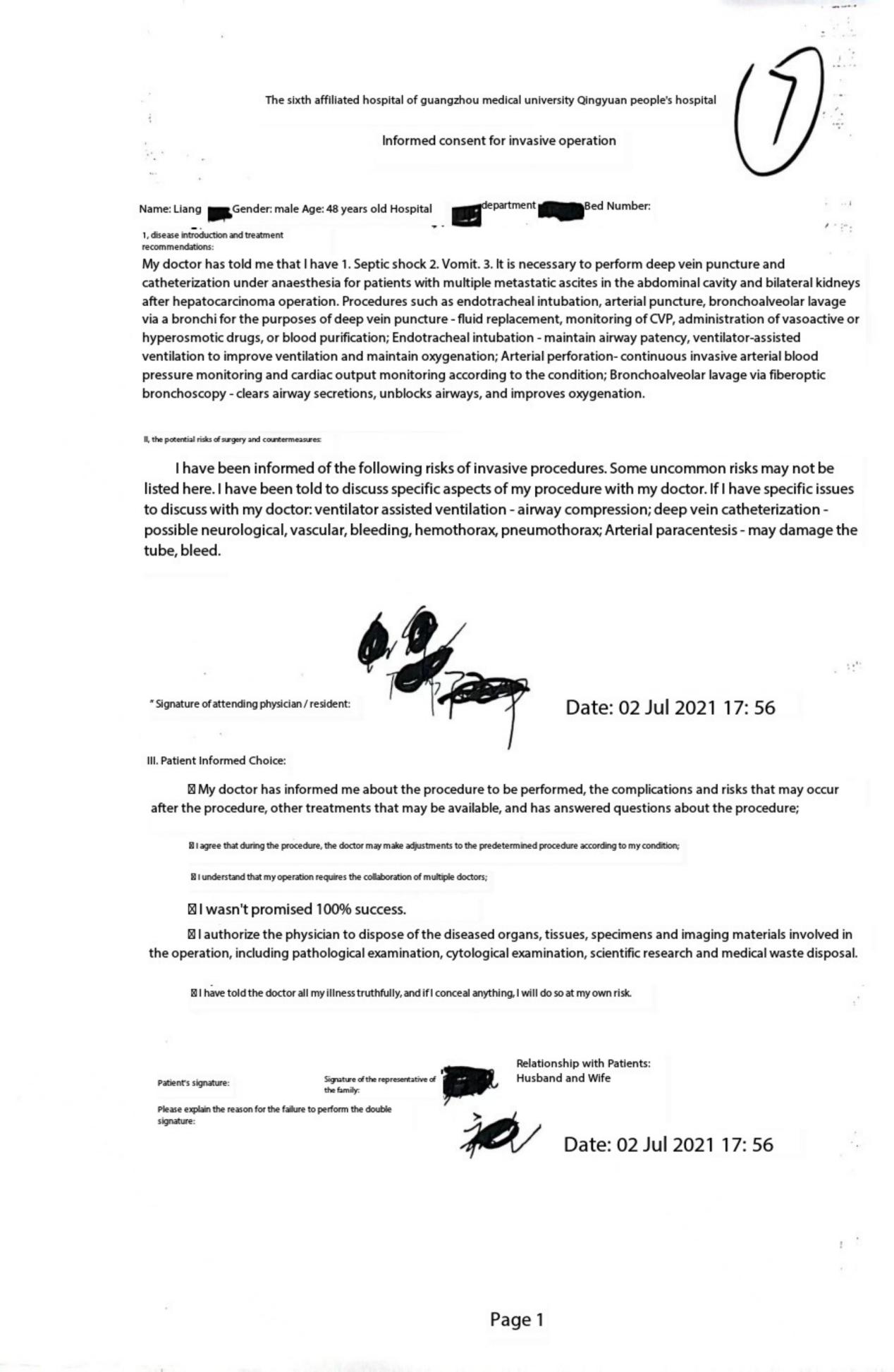

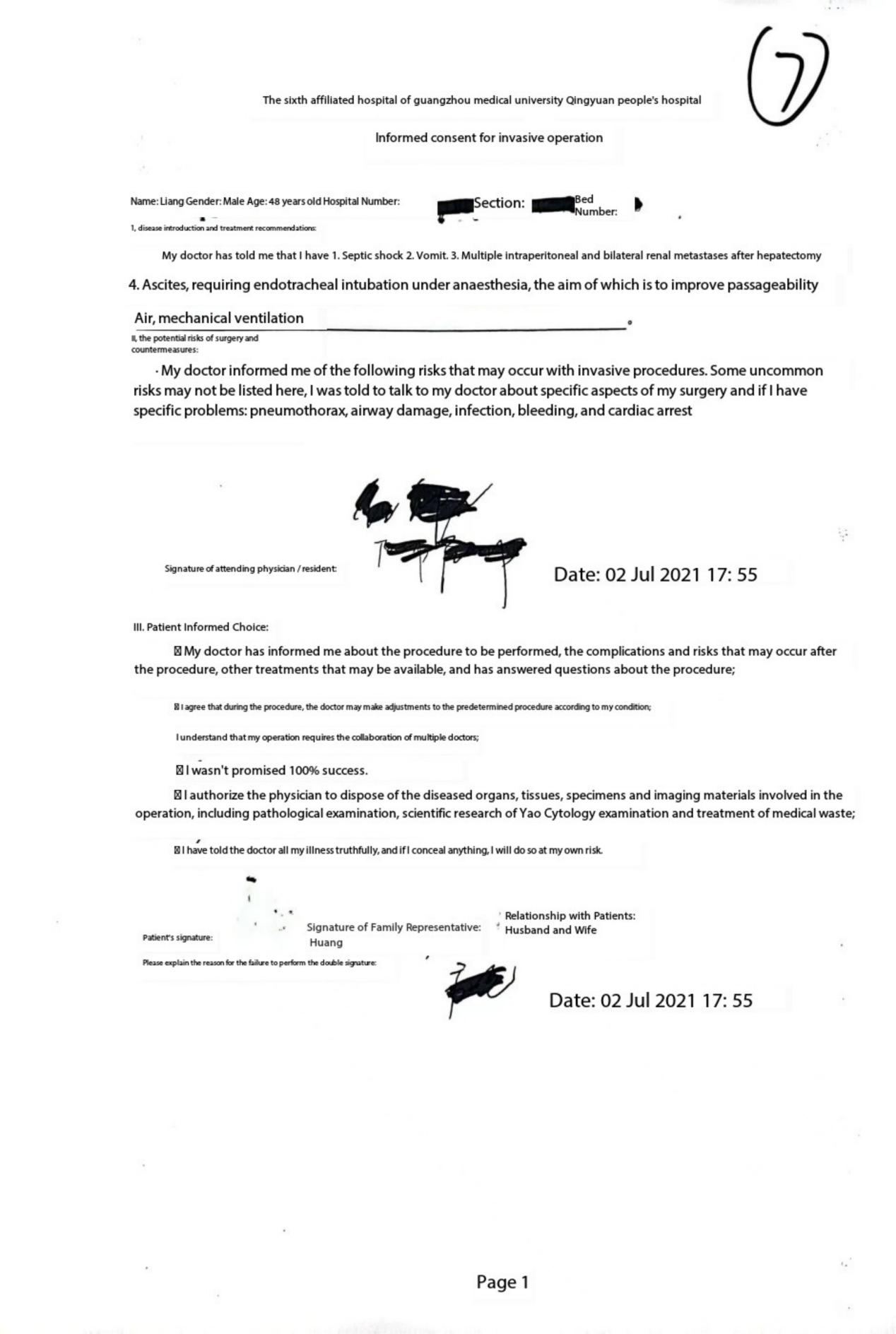

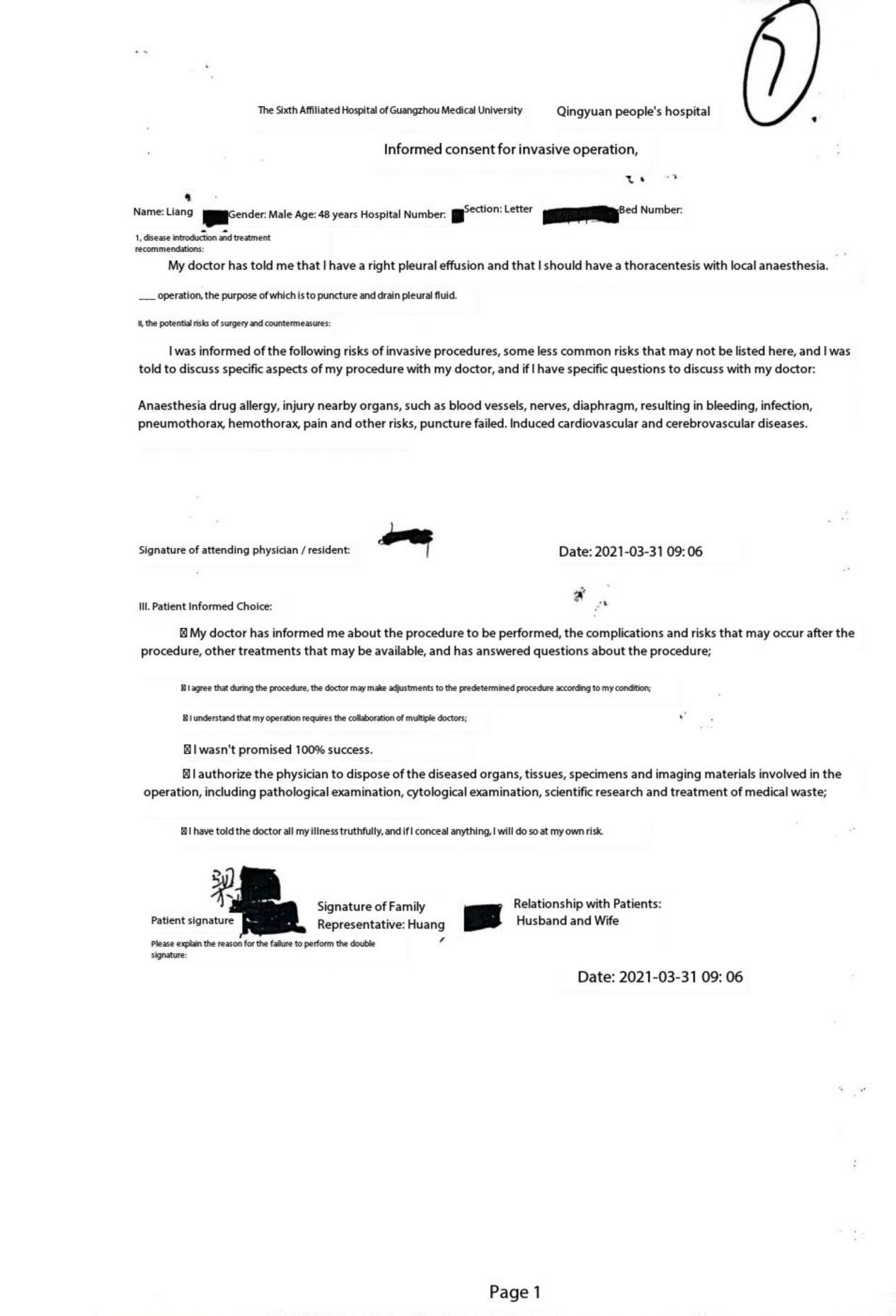
**

**Informed consent of HCC4**

**
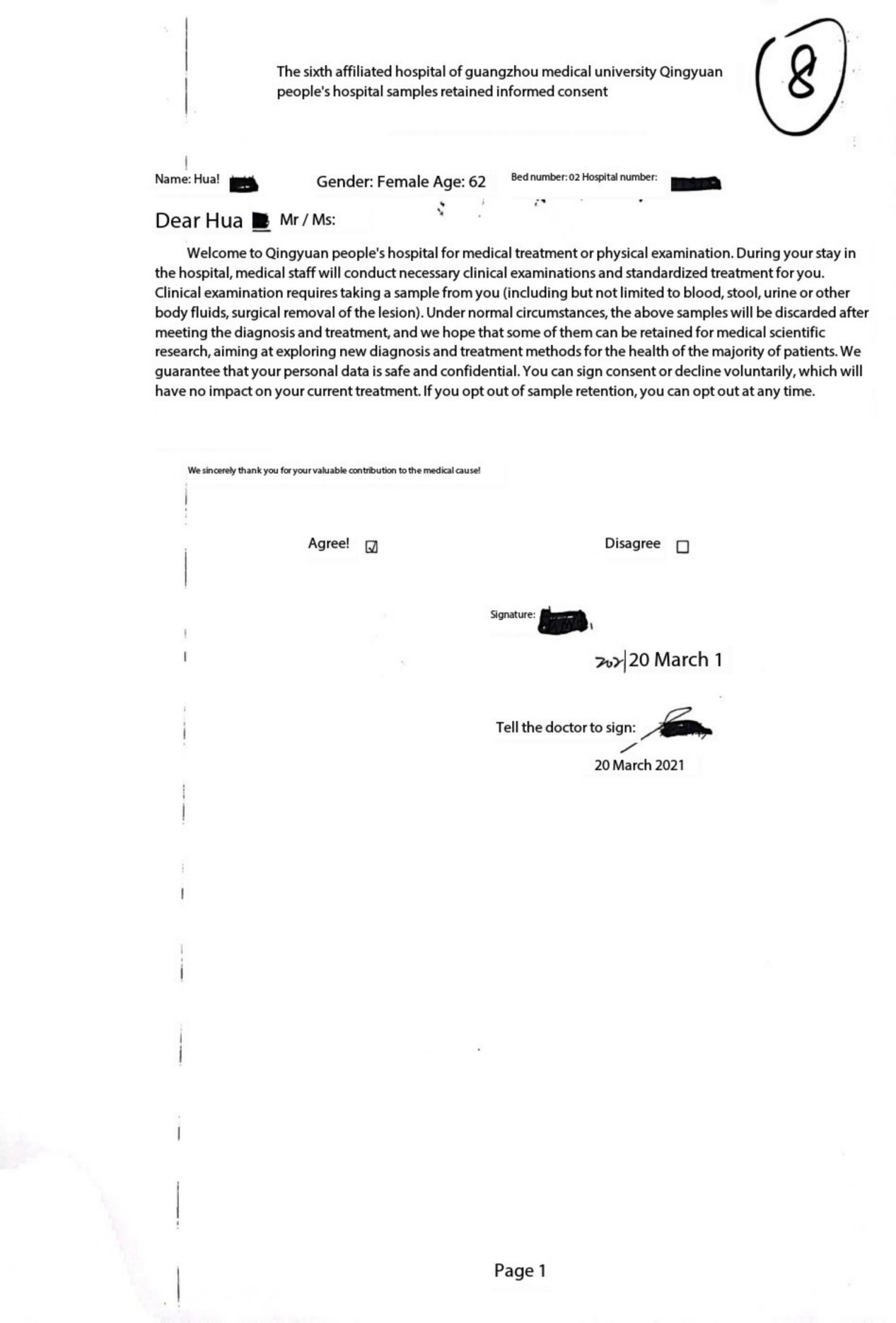
**

**Informed consent of HCC5**

**
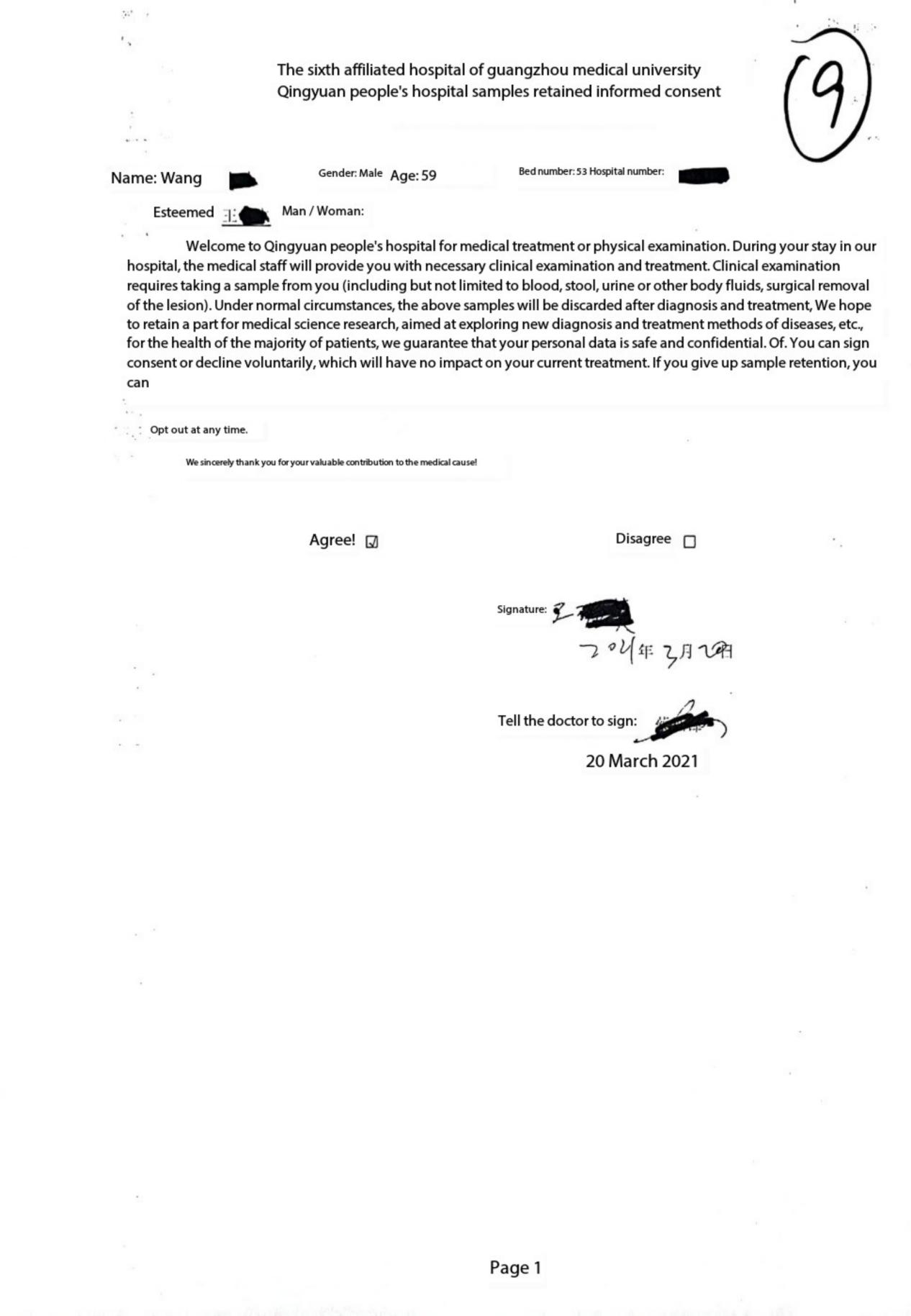
**
