# Supplementary material for: Unraveling the HGF/MET axis in Mallory-Denk body pathogenesis associated with liver fibrosis through single-cell transcriptomics
Source: Signal Transduct Target Ther. 2026 Jun 11;11:227. doi: 10.1038/s41392-026-02722-4 (PMC13254210; doi:10.1038/s41392-026-02722-4)
Supplement: Supplementary file 2 — Western blot original [file 41392_2026_2722_MOESM2_ESM.docx]

**Original Western blot**

**Figure 4i**

**
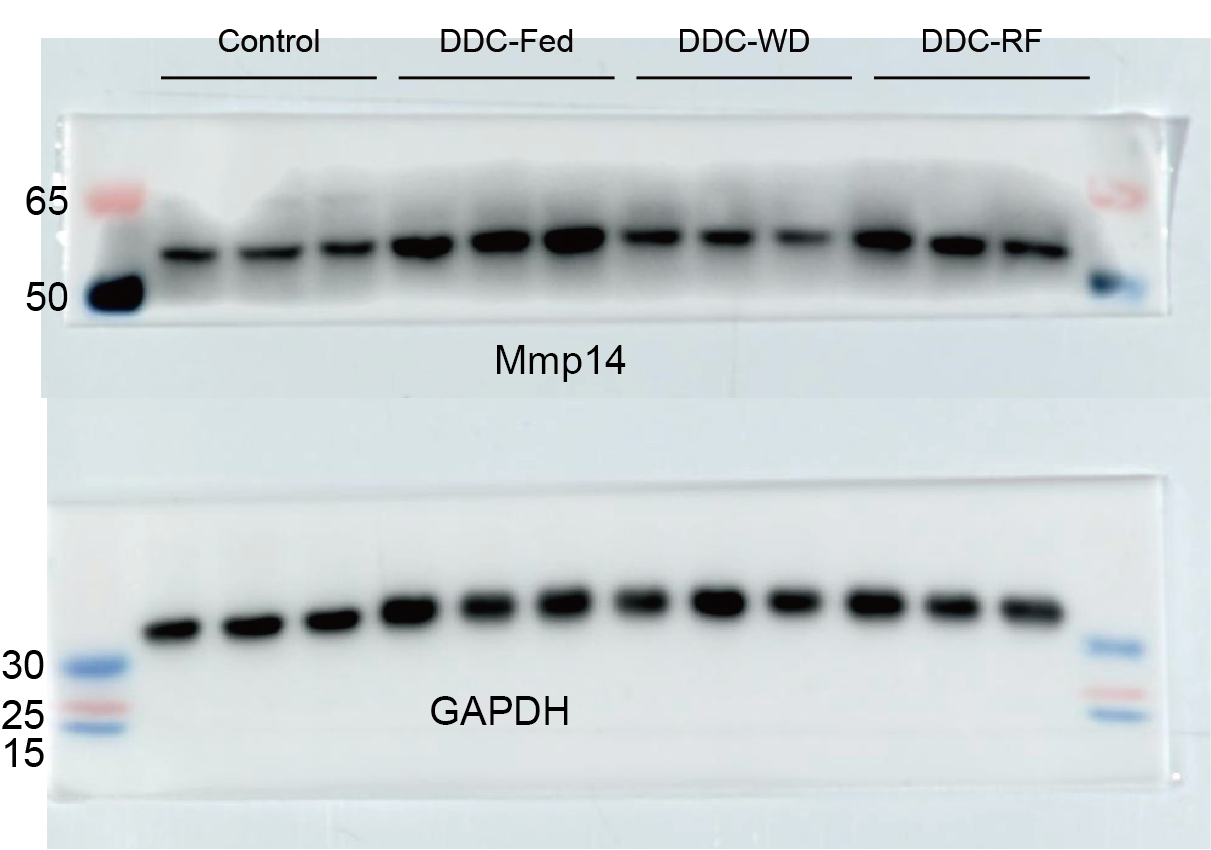
**

**Figure 4j**

**
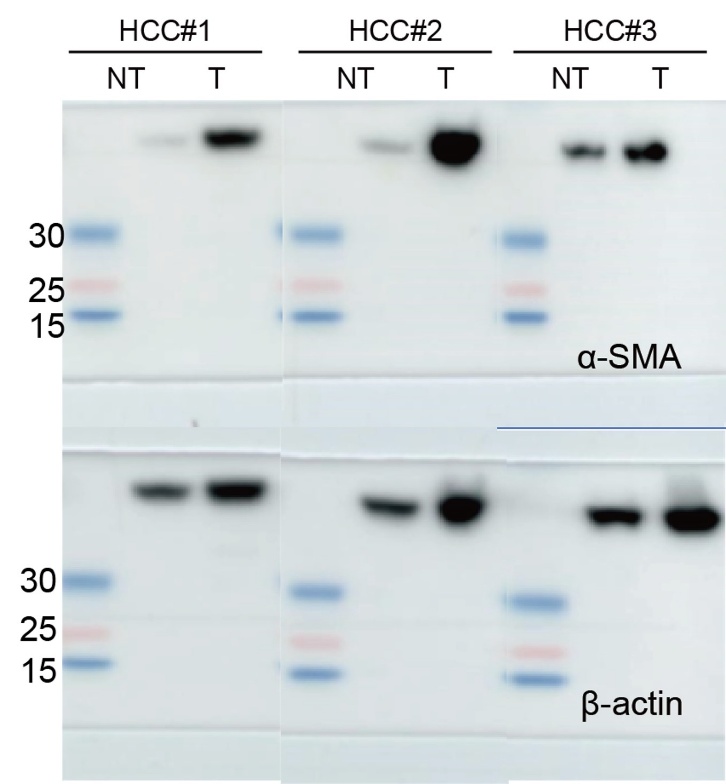

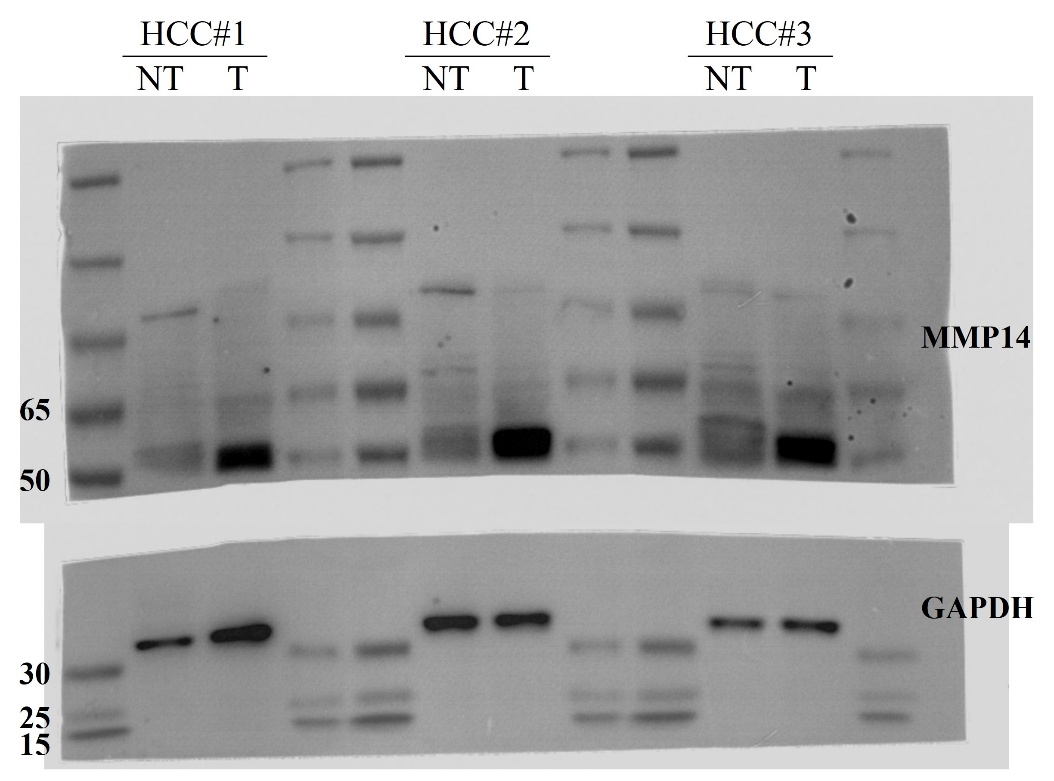
**

**Figure 5j**


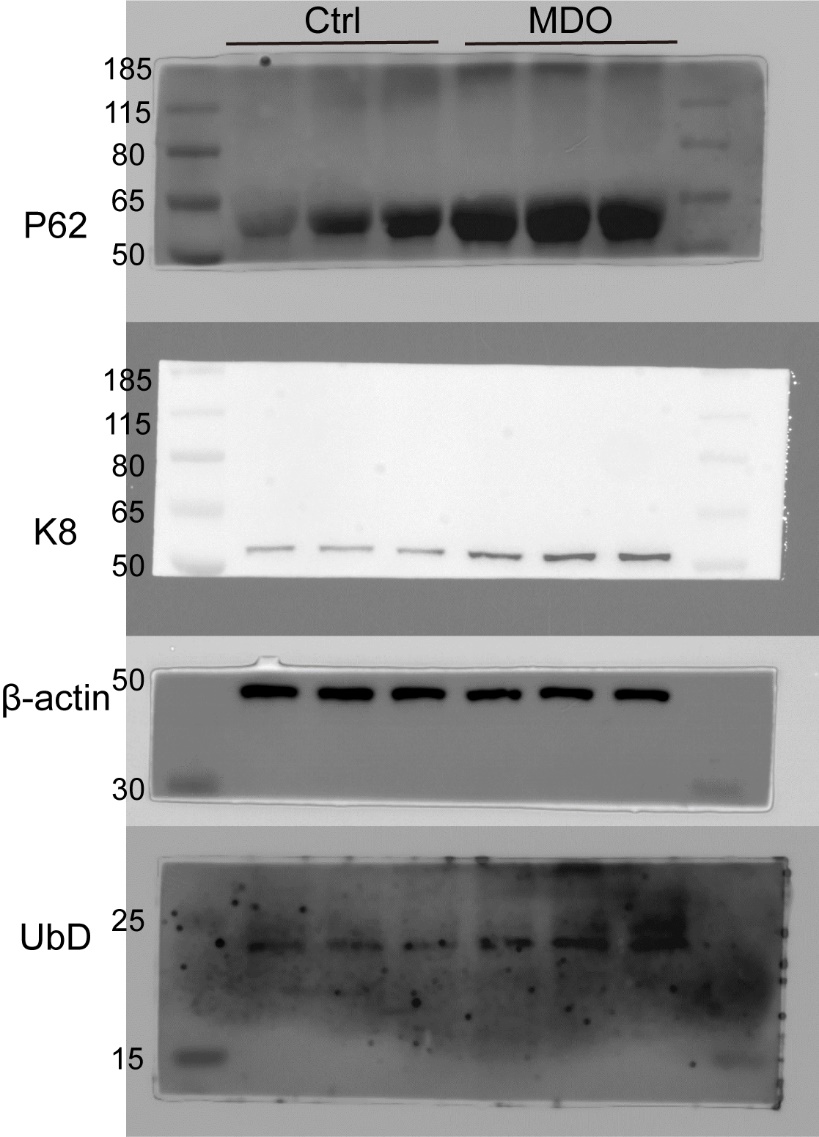


**Figure 6c**


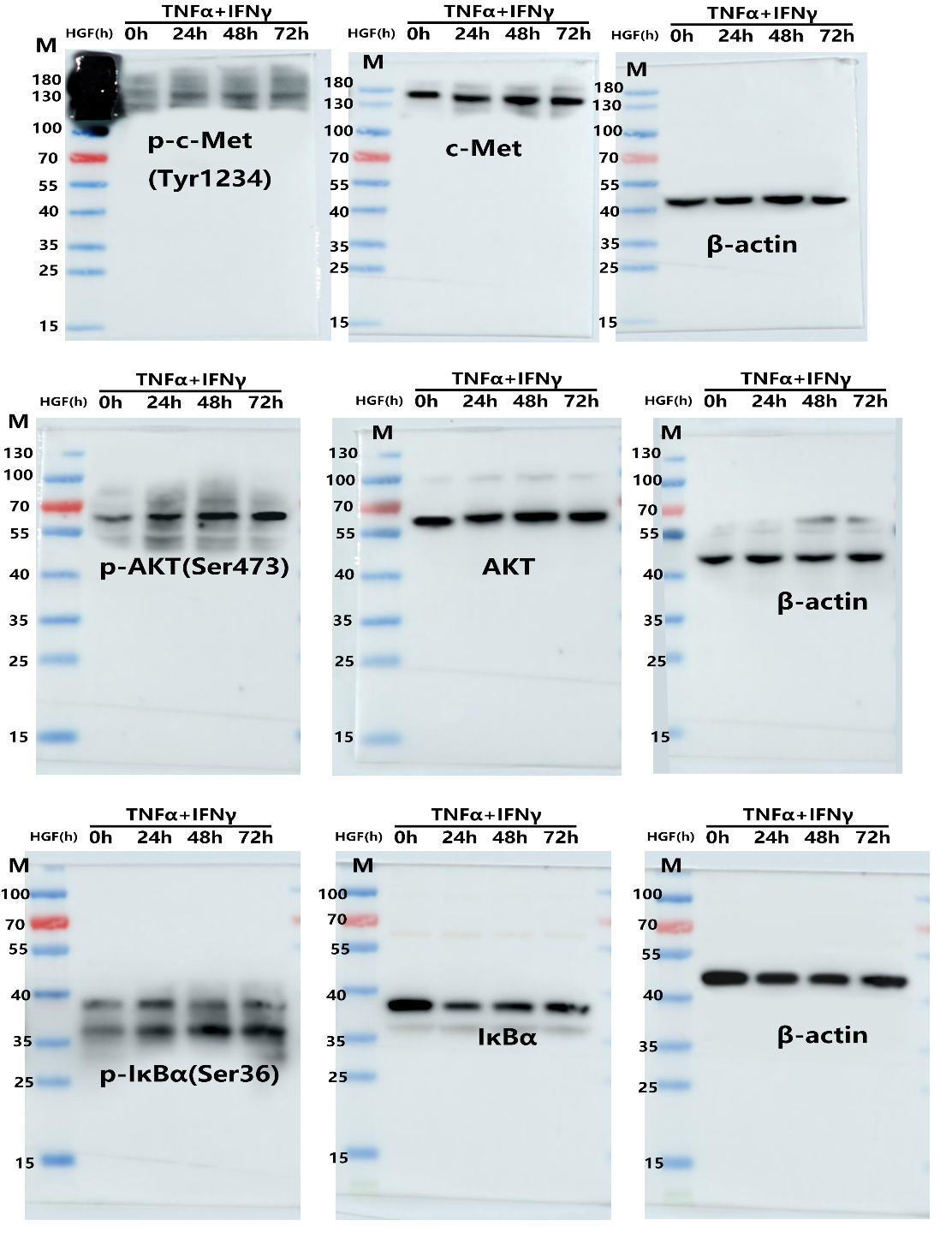


**Figure 6d**


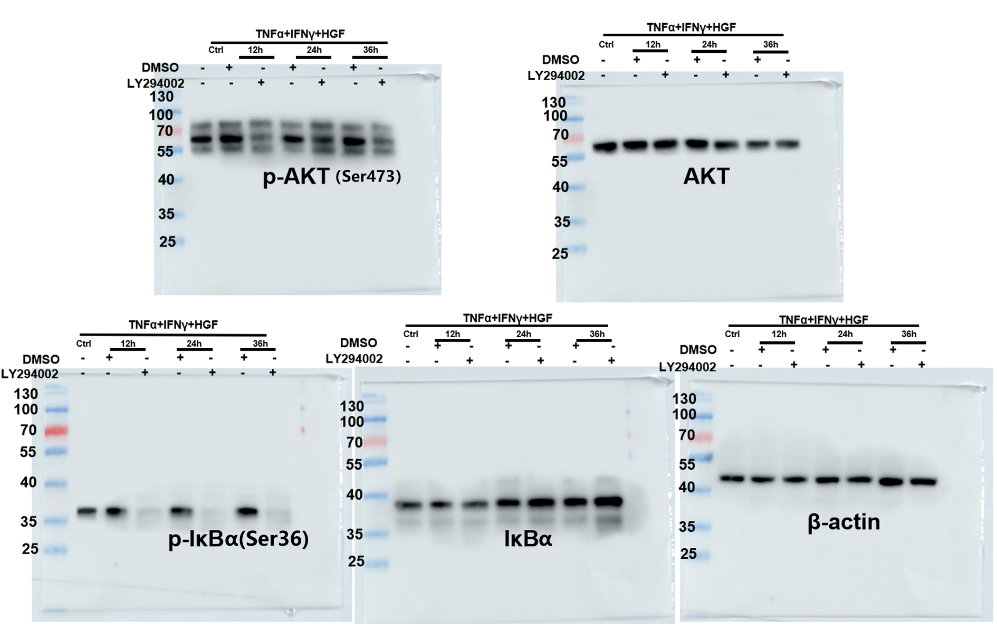


**Figure 6e**


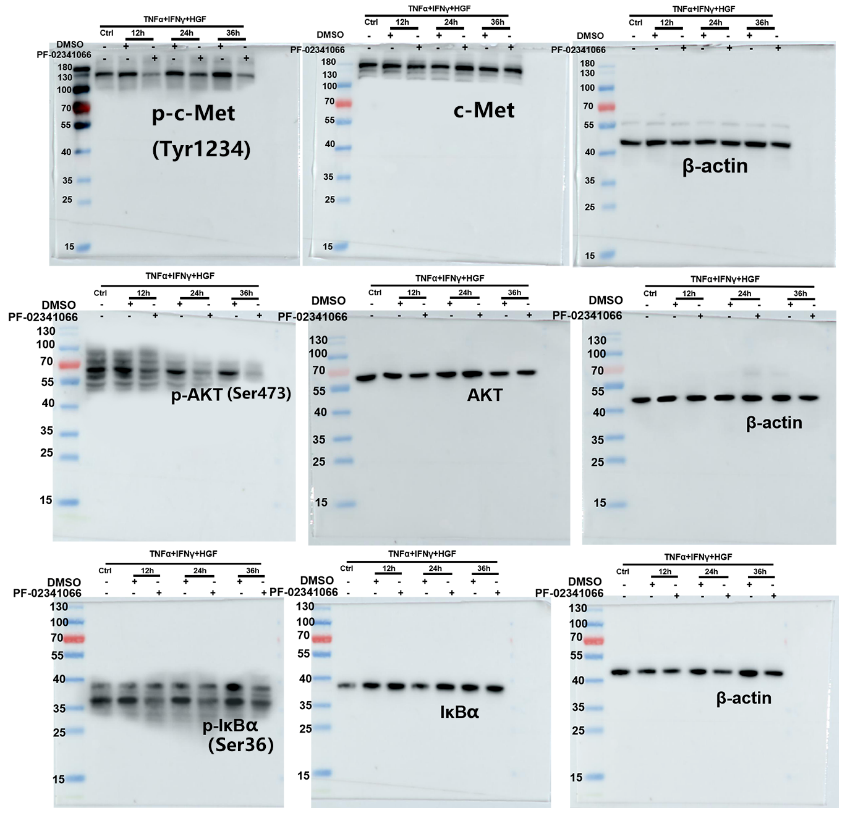


**Figure 6f**


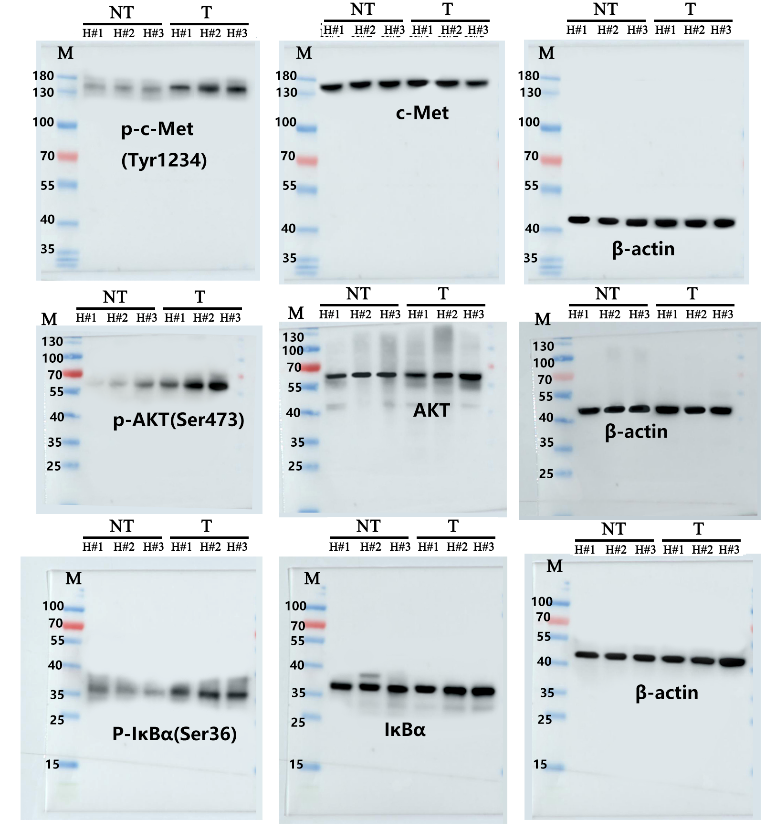


**Figure 7b**


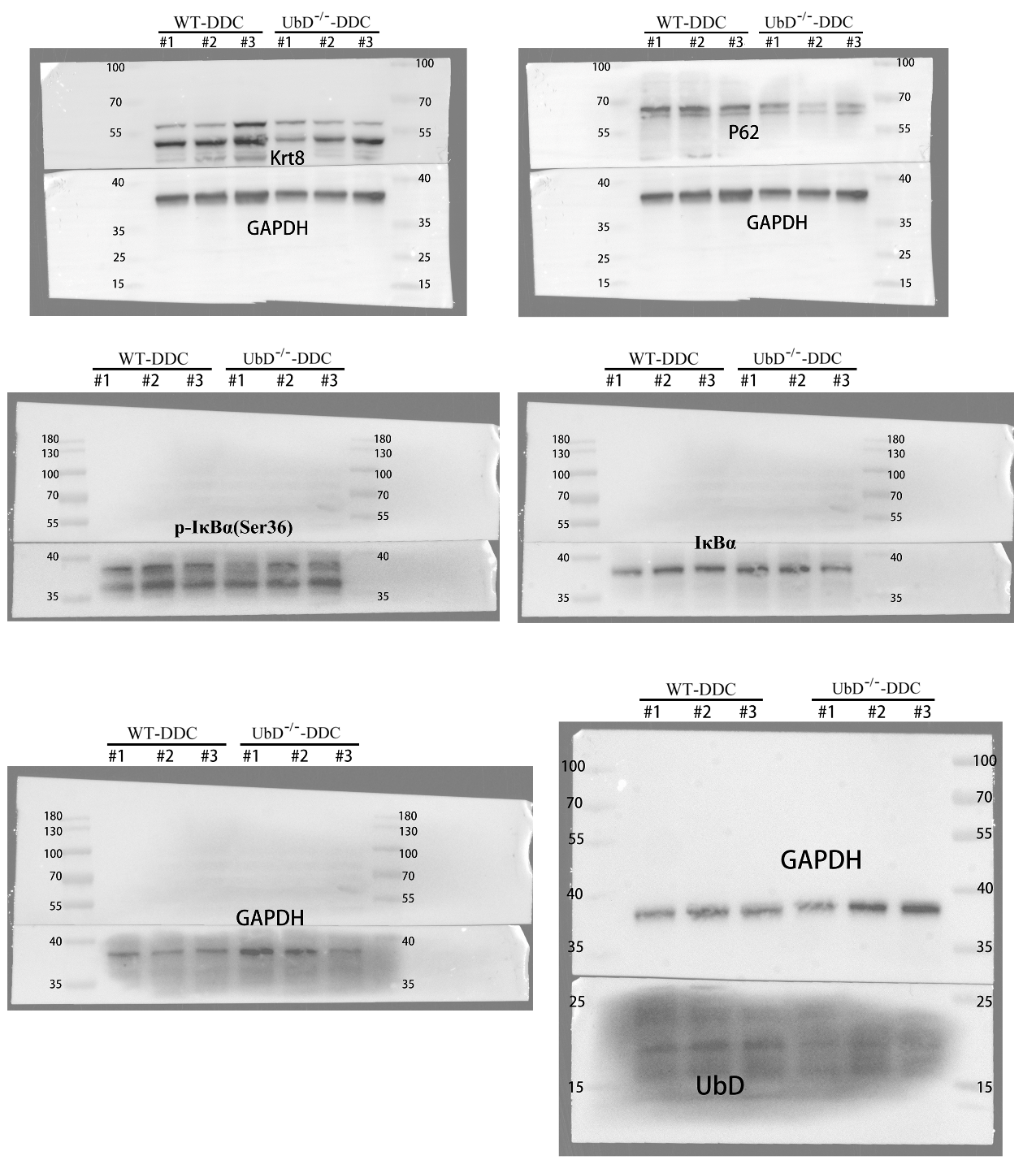


**Figure 7c**


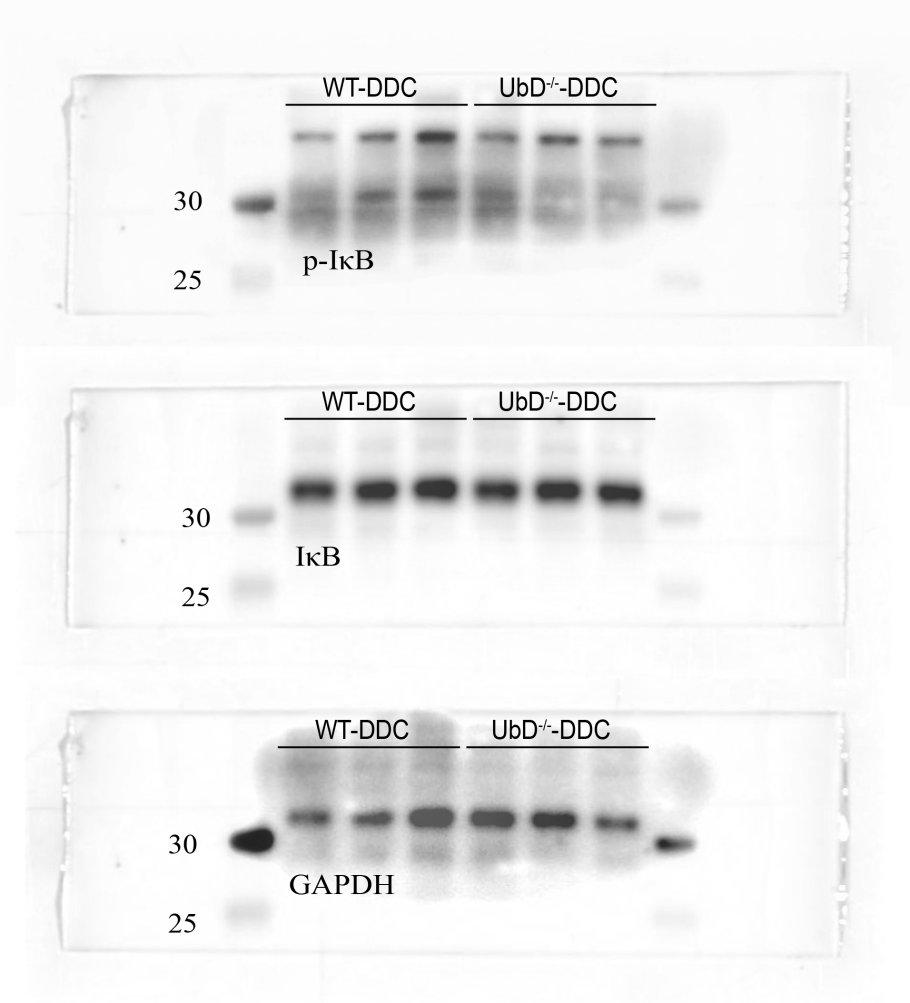


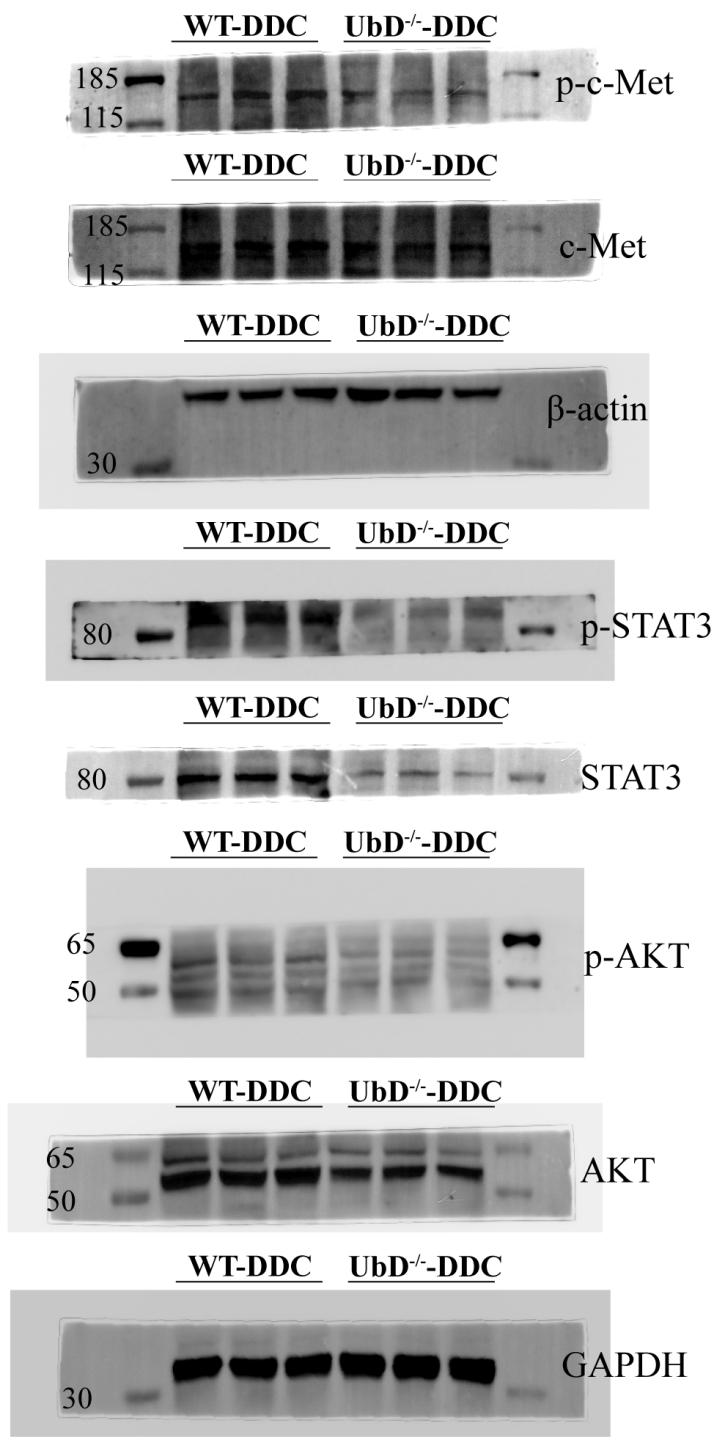


**Figure 7g**


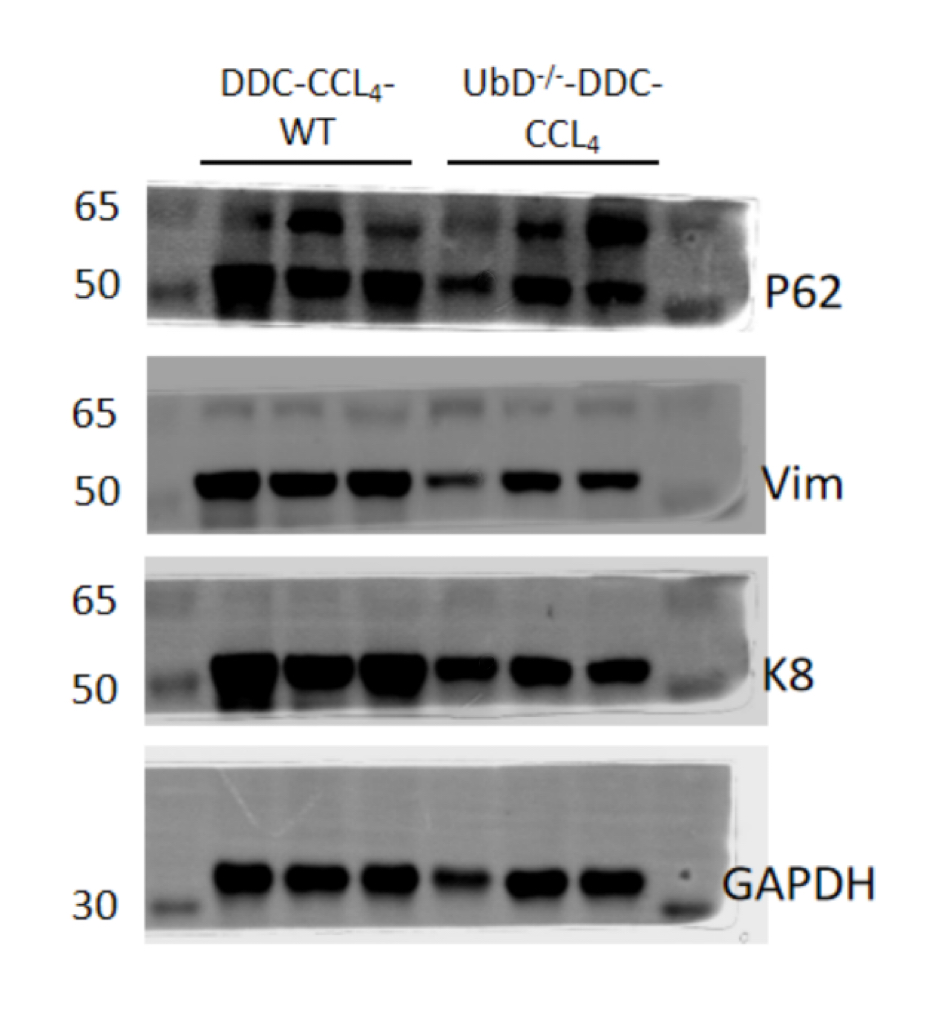


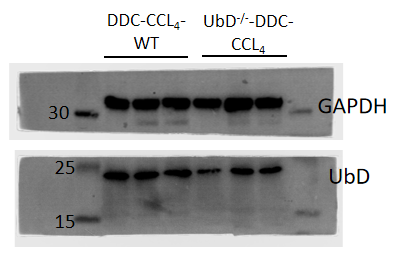


**Figure S18e**


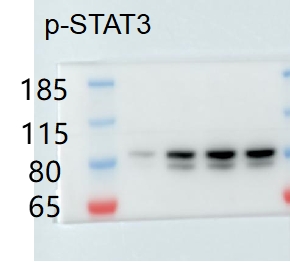

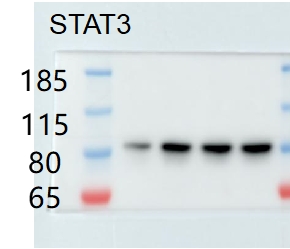

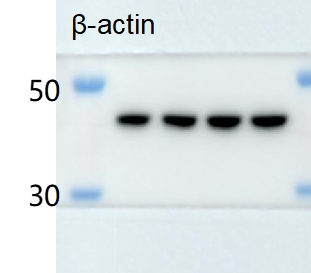

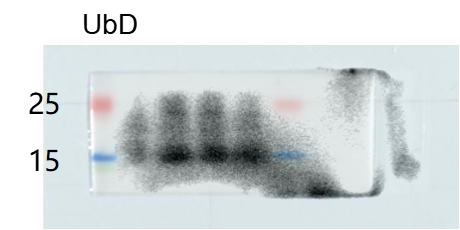

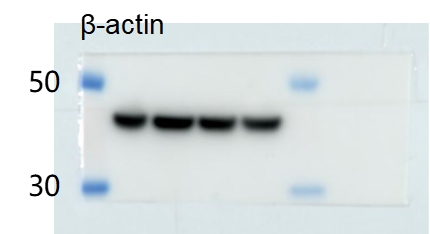


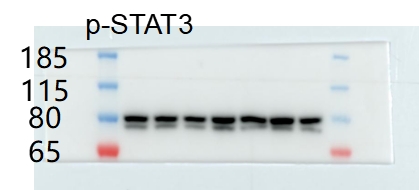

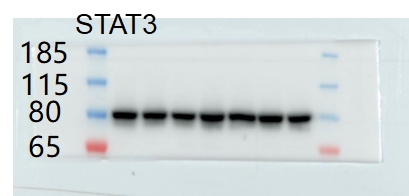

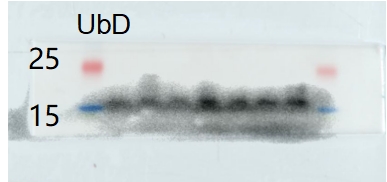

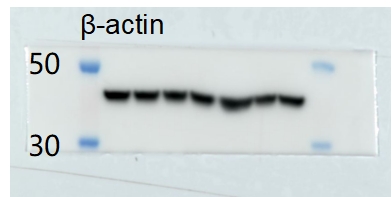


**Figure S20c**


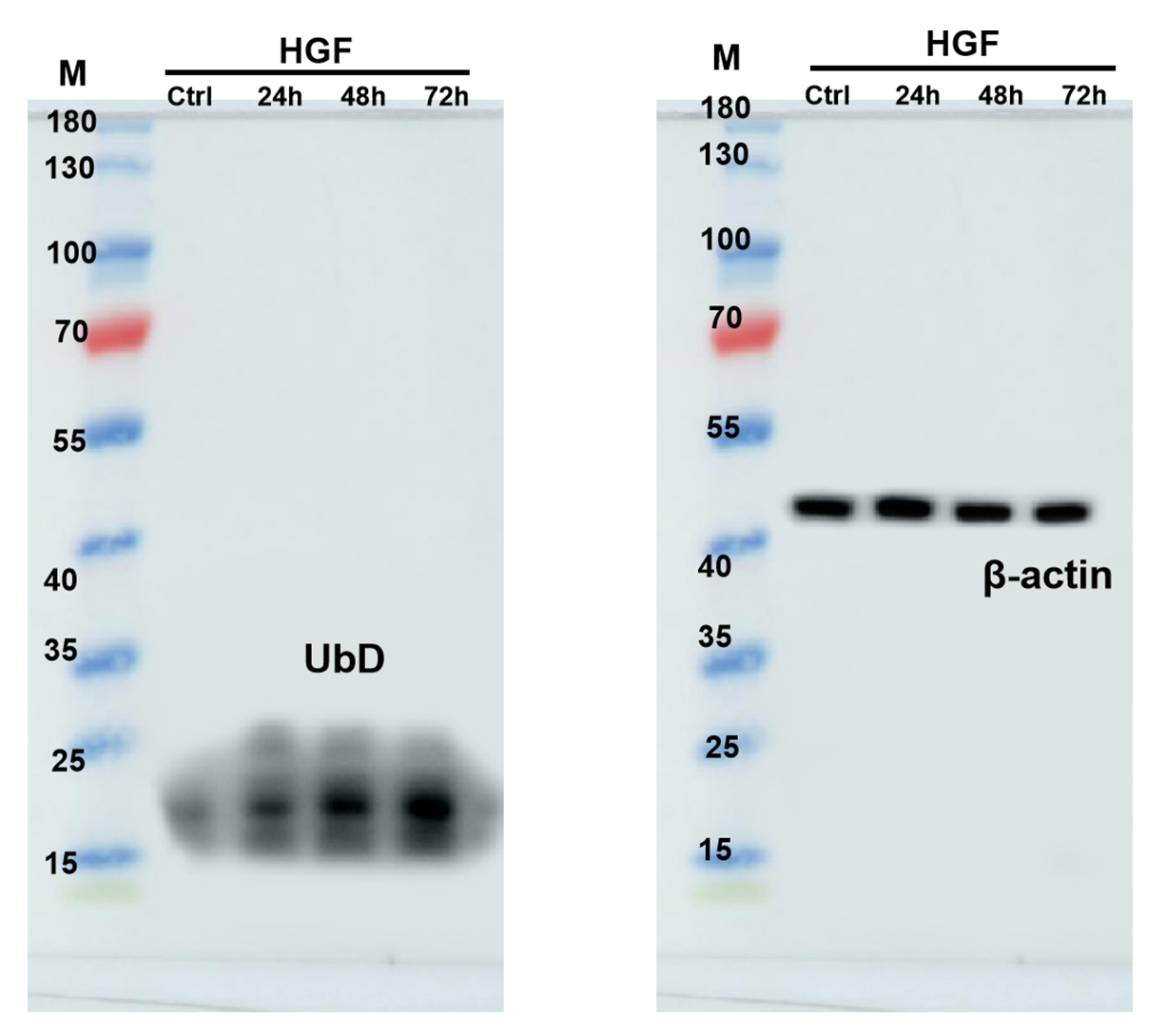


**Figure S20d**

**
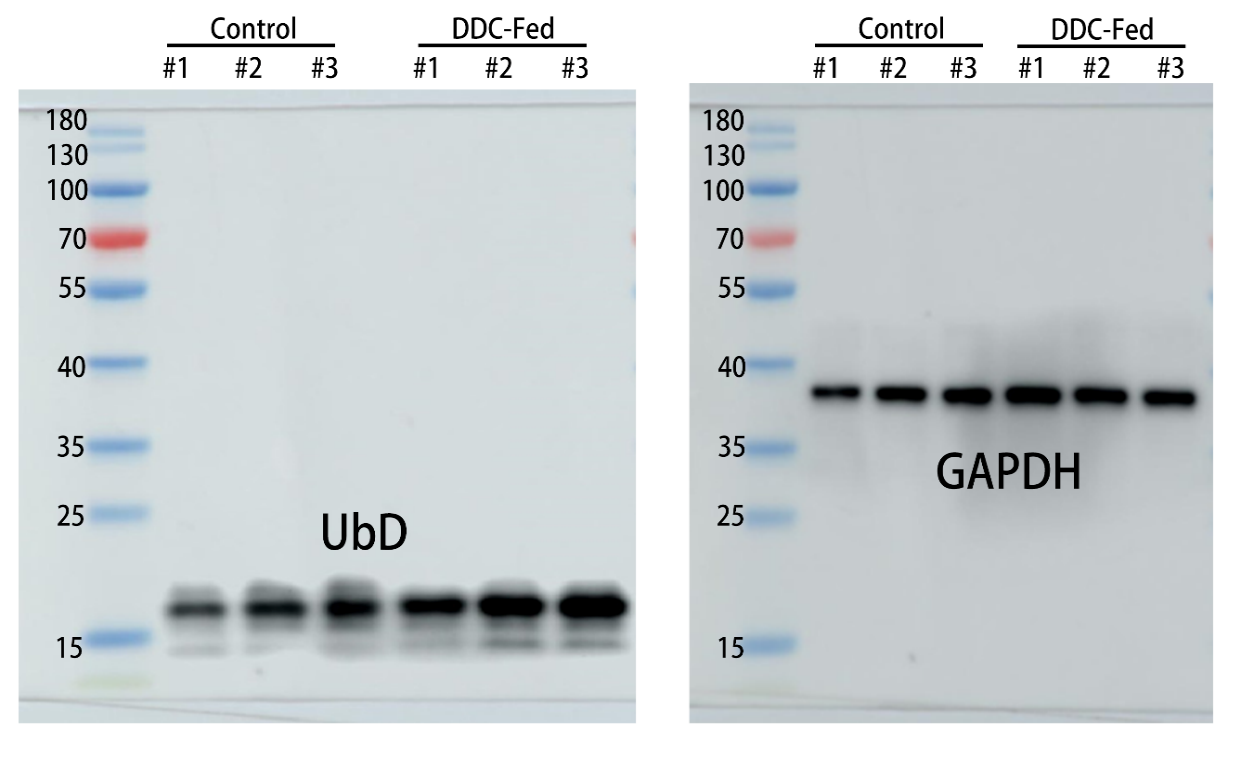
**

**Figure S20e**


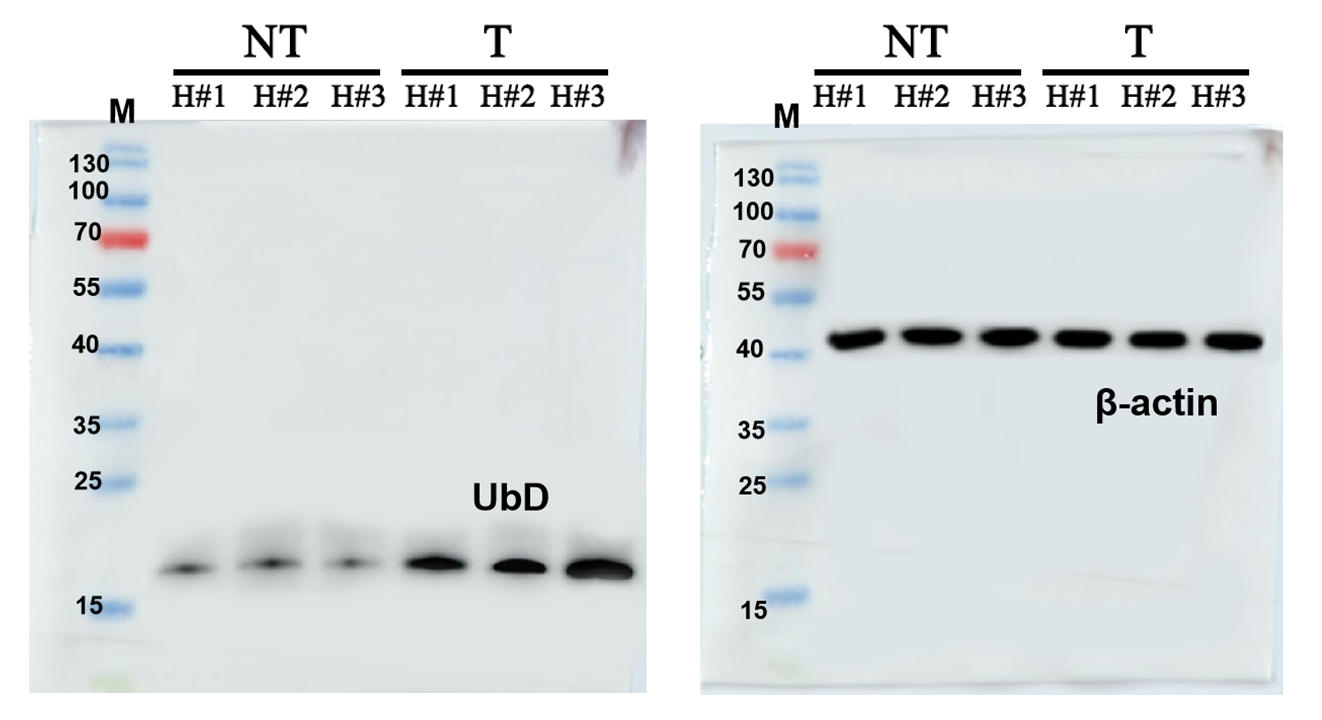


**Figure S20f**

**
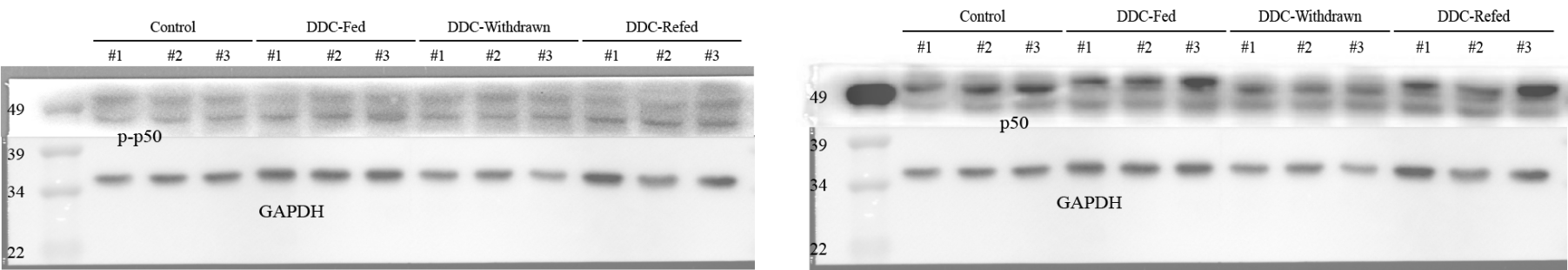
**

**Figure S21b**

**
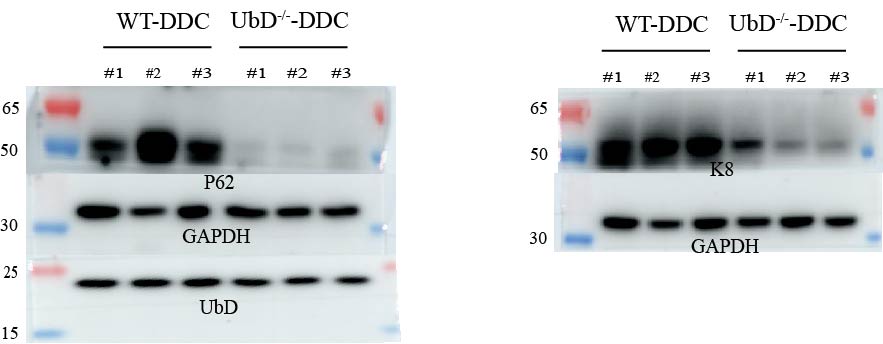
**
